# Fungal-fungal interaction between *Sanghuangporus vaninii* and its endophytic *Fusarium solani* rewires host secondary metabolism to boost bioactive metabolite production

**DOI:** 10.1186/s12934-026-02994-z

**Published:** 2026-04-04

**Authors:** Yanjun Ma, Lanlan Yu, Pengcheng Di, Xuetai Zhu, Xiaoyan Ma, Weibao Kong

**Affiliations:** 1 College of Life Sciences, Northwest Normal University, Lanzhou, China; 2 The First Clinical Medical College, Gansu University of Chinese Medicine, Lanzhou, China

**Keywords:** S*anghuangporus*, Co-culture, Endophytic fungus, Integrated omics, Bioactive metabolites

## Abstract

**Background:**

The medicinal mushroom *Sanghuangporus vaninii* produces valuable bioactive compounds, but yields are low in artificial culture. While co-culture with microbes can elicit production, the regulatory potential of native endophytic fungi - which share an evolutionary history with their host - remains largely unexplored. In this study, we report for the first time a co-culture system between *S. vaninii* and its endophytic fungus *Fusarium solani* MF20 to enhance the production of medicinal metabolites and elucidate the underlying mechanisms.

**Results:**

Co-culture with *F. solani* MF20 dramatically increased the yields of total flavonoids (9.38-fold), terpenoids (3.18-fold), and crude polysaccharides (4.87-fold) in *S. vaninii*. Integrated omics analyses revealed that the endophytic interaction induced global metabolic change in the host. Early signaling events, such as a controlled oxidative stress response, Ca^2+^ influx, extracellular ATP accumulation, and enhanced membrane permeability, were associated with the redirection of cellular resources from primary growth toward chemical defense. Key biosynthetic pathways, such as terpenoid backbone and flavonoid synthesis, were transcriptionally up-regulated, directly corroborated by the massive accumulation of bioactive compounds including the triterpene pachymic acid and complex modified flavonoids. Central carbon metabolism was reshaped, with activation of the pentose phosphate pathway potentially supplying NADPH for biosynthesis.

**Conclusions:**

This work demonstrates that a native endophytic fungus can act as a powerful biotic elicitor to unlock the metabolic potential of its medicinal fungal host. The co-culture strategy activates a stress-mediated defense response that reprograms primary and secondary metabolism, leading to overproduction of pharmaceutically relevant compounds. Beyond providing insights into fungal-fungal symbiotic interactions, this study validates endophyte-host co-culture as an effective and sustainable bioprocess technology for enhancing the production of high-value metabolites from medicinal fungal resources.

**Graphical Abstract:**

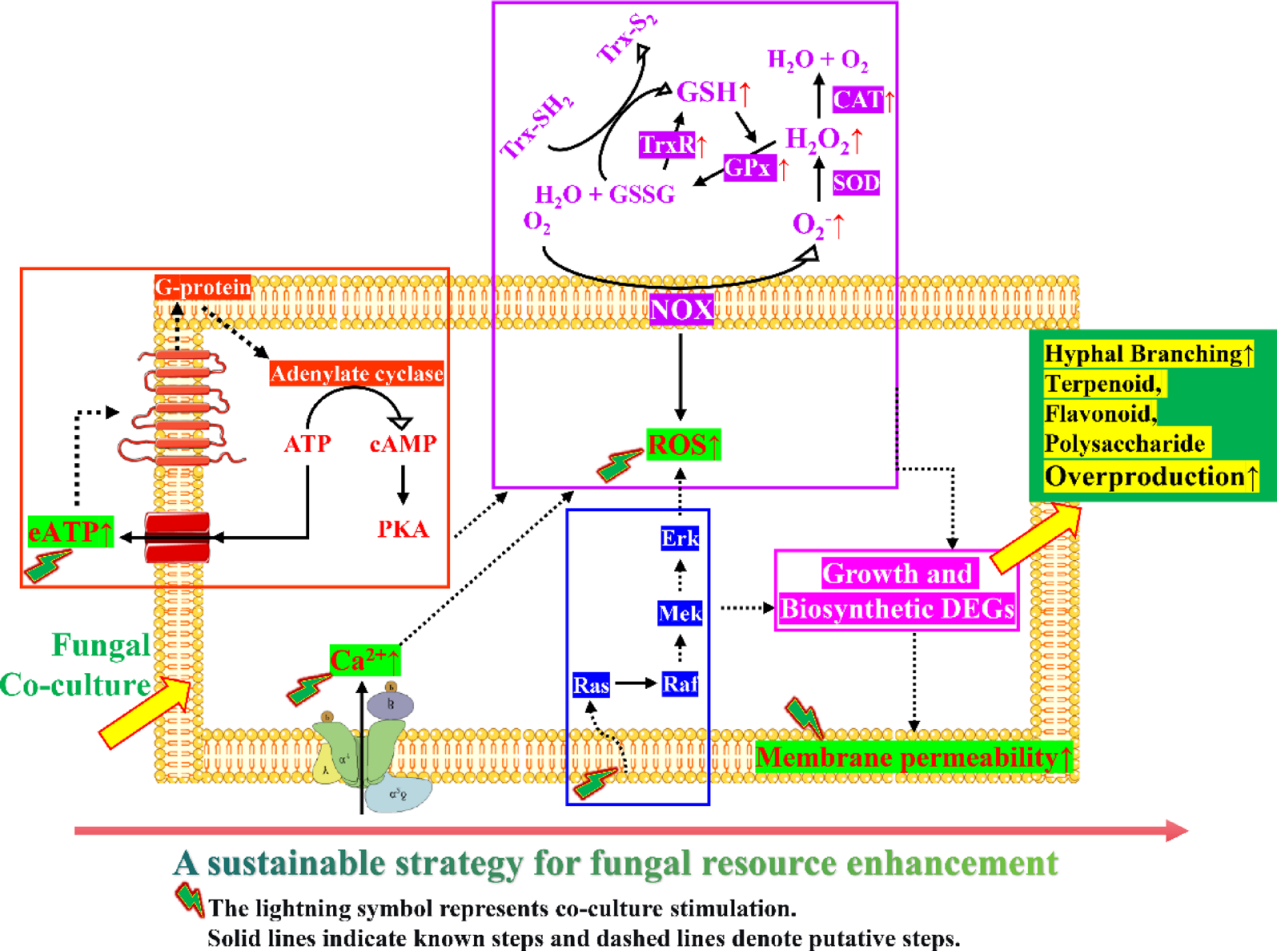

**Supplementary Information:**

The online version contains supplementary material available at 10.1186/s12934-026-02994-z.

## Highlights

- First report of co-culturing *S. vaninii* with its native endophyte *F. solani* as a biotic elicitor.
- Flavonoid, terpenoid, and polysaccharide yields increased by up to 9.38-fold.
- Integrated multi-omics deciphers the molecular mechanism of metabolic reprogramming.
- Defense-related signaling (ROS, Ca^2+^) and membrane remodeling drive productivity gain.
- Establishes a sustainable endophyte-host co-culture strategy for enhanced production of medicinal fungal metabolites.

**Supplementary Information:**

The online version contains supplementary material available at 10.1186/s12934-026-02994-z.

## Introduction

Medicinal-edible macrofungi represent an invaluable reservoir of natural pharmaceuticals [1, 2]. Within this group, the genus *Sanghuangporus* holds a prominent position, traditionally revered as “forest gold” due to its exceptional medicinal properties [3, 4]. Modern studies have substantiated its antitumor [5], antioxidant [6], anti-inflammatory [7], and immunomodulatory effects [8], attributed to its rich content of polysaccharides, terpenoids, and flavonoids. Among approximately 15 reported species [9], *S. vaninii* has emerged as a commercially pivotal cultivar prized for its robust growth and superior bioactivity [10–12]. However, the sustainable exploitation of *Sanghuangporus* is constrained by wild resource scarcity and low, unstable yields of active metabolites in artificial cultivation [13].

Conventional strategies to enhance metabolite production, such as mutagenic breeding [14], fermentation optimization [15, 16], extraction refinement [17], and elicitor addition [18, 19], have achieved moderate success. More recently, the One Strain Many Compounds (OSMAC) strategy, particularly co-cultivation, has shown great promise by activating silent biosynthetic gene clusters through microbial interactions [20]. This strategy has been successfully applied across diverse fungal systems: co-culture of *Aspergillus niger* and *A. oryzae* increased extracellular enzyme [21], while *Funalia floccosa* co-cultured with *Penicillium commune* boosted laccase yield [22]. Notably, an initial study on *S. vaninii* co-cultured with *Pleurotus sapidus* demonstrated increased polysaccharide production and associated metabolic alterations [23, 24]. Despite this progress, current research has predominantly focused on unrelated fungal pairs lacking natural ecological associations [25]. The regulatory influence of native endophytic fungi on their host fungi remains poorly understood. Although Zheng et al. [26] demonstrated that co-culture of endophytic *Fusarium* sp. SF12 with its host *Shiraia* sp. S9 enhanced hypocrellin A production, the underlying mechanisms remain unknown. Thus, investigating the interaction between *S. vaninii* and its native endophytes represents a promising frontier for unlocking its metabolic potential.

Endophytic microorganisms reside within host tissues without causing apparent disease, forming complex symbiotic relationships [27, 28]. While the concept originated from plant-fungus interactions, endophytes are now recognized as integral components of the macrofungal microbiome. Current research on endophytic communities within fungal fruiting bodies has largely emphasized bacterial diversity [29–31], bioactivities [32–34], and their regulatory roles in hosts [35–37]. In contrast, the functional roles of endophytic fungi themselves remain underexplored. Existing reports on endophytic fungi in mushrooms highlight two aspects. First, many endophytic fungi are prolific producers of bioactive metabolites. For instance, an endophytic *Irpex lacteus* CHG05 isolated from *Cordyceps hawkesii* produces cordycepin [38], while a *Porostereum umbrinoalutaceum* KUFC101 from *P. ostreatus* exhibits multi-metal tolerance, produces various hydrolytic enzymes, and promotes plant growth [39]. Second, regarding host interaction, most studies focus on growth and development. Examples include the mediated antagonism between *Grifola umbellata* and its endophyte against *Armillariella mellea* [40], the dual role of yeasts from boletes in interacting with the mycoparasite *Sepedonium chrysospermum* and the host fungus *Paxillus* [41], and the role of truffle-inhabiting fungi in secreting volatile compounds or regulating mycorrhizal synthesis [42–44]. However, direct evidence demonstrating their regulatory impact on host secondary metabolism is exceptionally scarce, with Zheng et al. [26] on hypocrellin A biosynthesis being a notable exception. Our previous work systematically characterized the endophytic fungal community within *S. vaninii* fruiting bodies [45]. Intriguingly, these endophytes were predicted to exhibit diverse trophic modes, including Pathotroph, Saprotroph, and Symbiotroph. This finding led us to hypothesize that these intimately associated endophytic fungi may play previously unrecognized roles in regulating host growth, development, or medicinal metabolite biosynthesis. However, this hypothesis primarily stems from omics-based predictions and remains speculative. Consequently, a significant knowledge gap exists: whether and how native endophytes influence the growth and metabolism of *S. vaninii*, and the underlying molecular mechanisms are entirely elusive.

Building upon this foundation, the present study aimed to isolate and screen endophytic fungi from *S. vaninii* to identify those capable of positively regulating host bioactive compound accumulation. Our integrated strategy involved: (i) preliminary screening of isolates via a plate-based co-culture system [46], (ii) molecular identification of positive strain, and (iii) optimization of co-culture conditions. To decipher the underlying molecular mechanisms, we employed integrated transcriptomic and metabolomic approaches, which have proven powerful for unraveling regulatory networks in medicinal-edible fungi [47–51] and have been successfully applied within *Sanghuangporus* to dissect how external stimuli adjust metabolic pathways [15, 52–57]. We hypothesize that a specific endophytic fungus can regulate the metabolic network of *S. vaninii*, thereby enhancing the biosynthesis of target compounds.

**Fig. 1:**
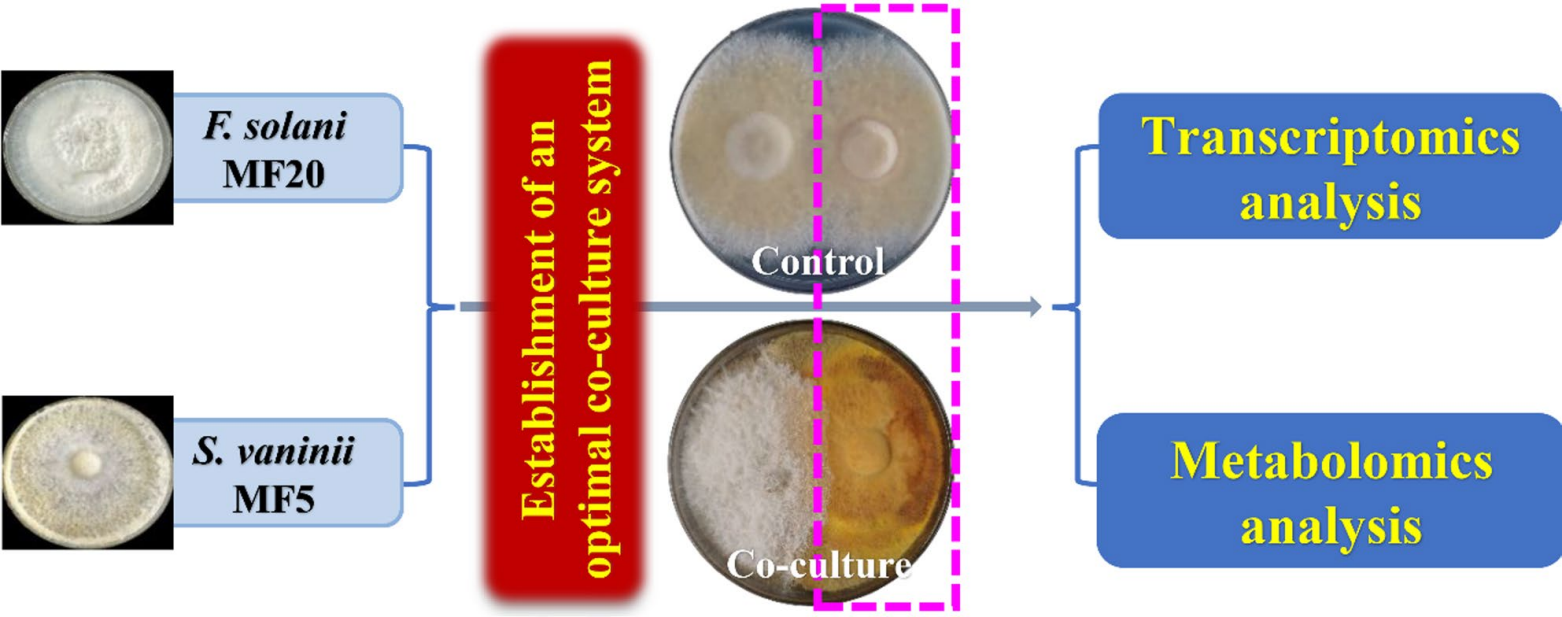
Schematic overview of the experimental design. The study integrates a co-culture system of *S. vaninii* MF5 with its native endophytic fungus *F. solani* MF20, coupled with multi-omics analyses (transcriptomics and metabolomics) to decipher the mechanisms underlying enhanced bioactive metabolite production

To this end, as outlined in Fig. 1, this work presents the first systematic investigation of the regulatory role of a native endophytic fungus (*F. solani* MF20) on the mycelial growth and bioactive metabolite accumulation of its host, *S. vaninii* MF5, while unraveling the underlying mechanisms via integrated multi-omics. Our findings provide novel insights into the unexplored domain of endophyte-host fungal interactions. Beyond its theoretical impact, this study establishes endophyte-host fungal interactions and establish endophyte-host co-culture as a new paradigm for enhancing high-value fungal metabolite production, thereby facilitating more efficient utilization of medicinal-edible fungal resources.

## Materials and methods

### Strains and culture conditions

The collection and processing of *S. vaninii* fruiting bodies, as well as the isolation of endophytic fungi, were performed according to our previously described methods [45]. A total of 37 endophytic fungal strains were isolated from fresh fruiting bodies. Among these, the host strain *S. vaninii* MF5 (GenBank accession number (No.) OR681008) and the endophytic fungus *F. solani* MF20 (accession No. OR672754) were successfully isolated and subsequently deposited at the China Center for Type Culture Collection (CCTCC) under accession No. CCTCC AF 2,023,066 and CCTCC M 20,232,175, respectively. Both strains were maintained on potato dextrose agar (PDA) slants and stored at 4℃. For activation, strains MF5 and MF20 were incubated on PDA plates at 28℃ for 2 days, and then subcultured onto fresh PDA medium. All cultures were incubated upside down at 28℃ in a constant-temperature incubator.

### Co-culture assay and molecular identification of the endophytic fungus

**Fig. 2:**
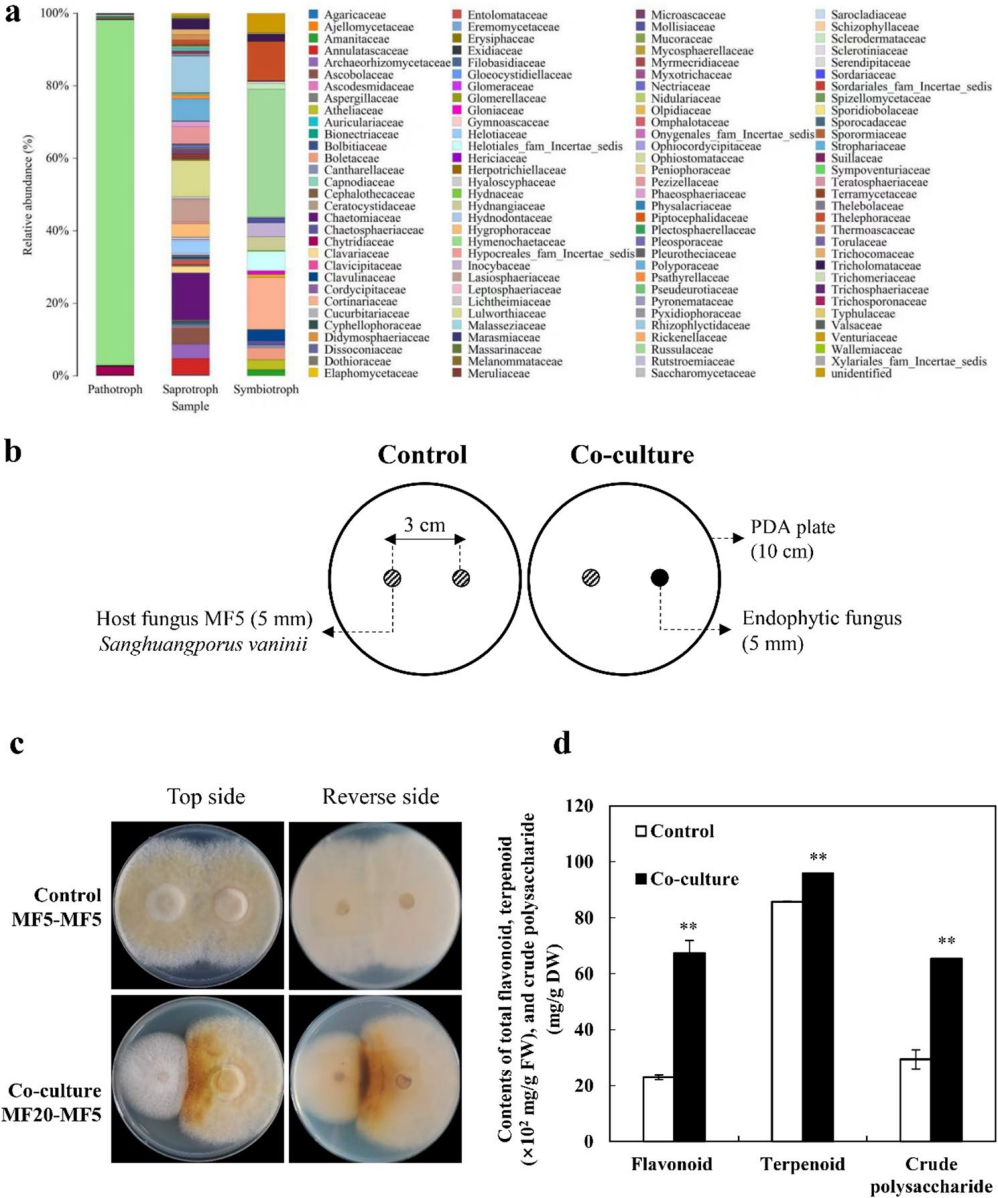
Trophic mode prediction of the endophytic fungal community in *S. vaninii* and the initial stimulatory effect of isolate *F. solani* MF20 on the host. **a** Predicted trophic modes (Pathotroph, Saprotroph, Symbiotroph) of endophytic fungi at the family level within *S. vaninii* fruiting bodies. **b** Schematic of the plate-based dual-culture assay for screening endophytic fungi. **c** Phenotypic alteration of MF5 upon co-culture with endophytic strain MF20. **d** Quantitative analysis of total flavonoids, terpenoids, and crude polysaccharides in MF5 after initial co-culture with MF20. Data are presented as mean ± SD from three independent biological replicates (*n* = 3). Statistical significance was determined by Student’s *t*-test (*p < 0.05, **p < 0.01 vs. monoculture control)

To investigate the influence of the endophytic fungi on the host *S. vaninii* MF5, a plate co-culture assay (Fig. 2b) was established following the method described by Chen et al. [46]. Briefly, activated fungal plugs (0.5 cm in diameter) of strain MF5 were inoculated on both sides of a 10 cm-diameter PDA plate as the control group. For the treatment group, plugs of strain MF5 and the endophytic fungi were inoculated symmetrically on the same type of plate, with a distance of 3 cm between the two plugs. All plates were incubated at 28 °C. The fungal-fungal co-culture assay revealed that endophytic strain MF20 significantly promoted pigment and metabolite accumulation in the host strain MF5. To further optimize the timing of MF20 inoculation, MF20 was introduced into co-culture with strain MF5 at different host growth stages (0, 2, 4, 6, and 8 days). The effects on mycelial growth and metabolite synthesis of the host MF5 were evaluated; mycelial growth was observed by visual inspection of colony expansion, while metabolite synthesis was quantified by content determination. This led to the identification of the optimal inoculation time for enhancing the production of active metabolites in strain MF5.

For molecular identification of strain MF20, genomic DNA was extracted using a Fungal DNA Kit (Omega Bio-tek, Norcross, GA, USA). The ITS rDNA region was amplified with universal fungal primers ITS1 (5’-TCCGTAGGTGAACCTGCGG-3’) and ITS4 (5’-TCCTCCGCTTATTGATATGC-3’). The polymerase chain reaction (PCR) mixture contained: 200 ng of DNA template, 1 µL of dNTPs, 1 µL of each primer, 0.25 µL of Taq DNA polymerase, and ddH_2_O to a final volume of 50 µL. Amplification was performed under the following conditions: initial denaturation at 94 °C for 3 min; 30 cycles of denaturation at 94 °C for 30 s, annealing at 56 °C for 30 s, and extension at 72 °C for 1 min; followed by a final extension at 72 °C for 5 min. The PCR products were stored at 4 °C before being sent for sequencing to Beijing Tsingke Biotech Co., Ltd. (Beijing, China). The obtained sequences were compared using the basic local alignment search too (BLAST, https://blast.ncbi.nlm.nih.gov/Blast.cgi) algorithm on the national center for biotechnology information (NCBI, https://www.ncbi.nlm.nih.gov/genbank/) database. A phylogenetic tree was constructed based on the ITS rDNA sequences using MEGA software (version 7.0.26) [58] and the Phylogeny.fr platform (http://phylogeny.lirmm.fr/phylo_cgi/index.cgi).

### Microscopic analysis of mycelial morphology and cell membrane integrity

To analyze the effect of the endophytic *F. solani* MF20 on the mycelial growth of host *S. vaninii* MF5, a cover slip was aseptically inserted into the PDA plate of MF5 on the 6th day of cultivation. The culture was then co-cultured with MF20 until the 16th day (10 days of co-cultivation following 6 days of mono-culture). The cover slip was removed, and the adhered mycelia were observed and imaged under an optical microscope (Yongxin Optics Co., Ltd., Ningbo, China).

To further evaluate the influence on cell membrane permeability, the membrane-impermeant fluorescent nucleic acid stain SYTOX Green (Biosharp, Hefei, China; Catalog No. BS358a) was employed according to a previously described method with modifications [59]. This dye penetrates only compromised membranes and exhibits a strong fluorescence enhancement (approximately 500-fold) upon binding to nucleic acids, with excitation/emission maxima at 488/538 nm. Mycelial samples from both mono-cultured and co-cultured *S. vaninii* MF5 were treated with 0.5 µM SYTOX Green and incubated for 10 min in the dark. Subsequently, the samples were rinsed three times with distilled water to remove unbound dye. The stained hyphae were mounted on glass slides and visualized using a fluorescence microscope (Yongxin Optics Co., Ltd., Ningbo, China). For quantification of fluorescence intensity, images were captured under identical exposure settings from at least three random microscopic fields per sample. The mean fluorescence intensity of stained hyphal regions was measured using ImageJ software (v1.53a, NIH, Bethesda, MD, USA) by analyzing the mean gray value after background subtraction. The relative fluorescence intensity was calculated by normalizing to the mean value of the axenic control group.

### Extraction and determination of flavonoid, terpenoid and crude polysaccharide

The major bioactive components of *S. vaninii*, including flavonoids, terpenoids, and polysaccharides [9], were extracted and quantified according to previously established methods with appropriate modifications. Flavonoid content was determined using the sodium nitrite-aluminum nitrate colorimetric method [60]. Briefly, fungal samples (MF5 region) were collected from PDA plates, rapidly ground into powder in liquid nitrogen, and 0.2 g of the powder was dissolved in 5 mL of 70% ethanol. The mixture was subjected to ultrasonic extraction (60 °C, 55 kHz) for 30 min, followed by centrifugation at 12,000 rpm for 10 min. A 1 mL aliquot of the supernatant was diluted to 5 mL with 70% ethanol. Then, 0.3 mL of 5% NaNO_2_ solution was added, mixed, and incubated at room temperature for 6 min. Thereafter, 0.3 mL of 10% Al(NO_3_)_3_ solution was added, mixed, and allowed to stand for another 6 min. Finally, 4 mL of 4% NaOH solution was added, and the mixture was incubated for 6 min before being diluted to 10 mL with 60% ethanol. After 12 min of reaction, the absorbance was measured at 510 nm using a UV-2800 spectrophotometer (Unico Instrument Co., Ltd., Shanghai, China). The total flavonoid content was calculated based on a standard curve of rutin and expressed as mg per gram of fresh weight (mg/g FW (fresh weight)) according to the following formula:

$$ \begin{aligned} & {\mathrm{Total}}~{\mathrm{flavonoid}}~{\mathrm{contents}}~\left( {{\mathrm{mg}}/{\mathrm{g}}~{\mathrm{FW}}} \right) \\ & \quad = \left( {m~ \times {\mkern 1mu} {\mkern 1mu} V~ \times {\mkern 1mu} N} \right)/M \times 100 \\ \end{aligned} $$

where *m* is the rutin equivalent concentration (mg/mL) determined from the standard curve, *V* is the total volume of the extract (mL), *N* is the dilution factor, and *M* is the sample mass (g).

Terpenoid content was measured using the vanillin-glacial acetic acid method [61]. The extraction procedure was identical to that used for flavonoids. A 100 µL aliquot of the extract was mixed with 0.4 mL of 5% vanillin-glacial acetic acid solution and 1.6 mL of perchloric acid. The mixture was heated in a 70 °C water bath for 15 min, cooled to room temperature, and then 4 mL of ethyl acetate was added. After 15 min of incubation, the absorbance was measured at 546 nm. The total terpenoid content was calculated based on a standard curve of oleanolic acid and expressed as mg per gram of fresh weight using the same formula as above.

Crude polysaccharide content was quantified using the anthrone-sulfuric acid method [62]. Samples (0.2 g) were dissolved in 5 mL of distilled water and heated in a boiling water bath for 60 min. Then, 4 mL of freshly prepared 0.2% anthrone reagent was added, and the mixture was heated at 100 °C for 15 min. After rapid cooling, the absorbance was measured at 620 nm. The polysaccharide content was calculated based on a glucose standard curve and expressed as mg per gram of fresh weight according to the same formula.

### Transcriptome sequencing and bioinformatic analysis

Based on the optimized co-culture system established above, *S. vaninii* MF5 was co-cultured with the endophytic fungus *F. solani* MF20 for 10 days, starting at the 6th day of MF5 growth. The control group consisted of MF5 in pure culture, while the treatment group comprised MF5 under optimized co-culture conditions with MF20. Samples of *S. vaninii* MF5 were collected, ground in liquid nitrogen, and stored at -80 °C for subsequent use. Total RNA was extracted using TRIzol reagent (Invitrogen, Carlsbad, CA, USA) according to the manufacturer’s instructions. RNA purity and concentration were assessed using a NanoDrop 2000 spectrophotometer (Thermo Fisher Scientific, Wilmington, DE, USA), and RNA integrity was evaluated using an Agilent 2100 Bioanalyzer (Agilent Technologies, Santa Clara, CA, USA). Subsequently, cDNA libraries were constructed using the VAHTS Universal V6 RNA-seq Library Prep Kit (Vazyme Biotech, Nanjing, China; Catalog No. NR612-02). The quality of the libraries was verified using the Agilent 2100 Bioanalyzer, and paired-end sequencing (150 bp) was performed on an Illumina NovaSeq 6000 platform (Illumina, San Diego, CA, USA). All sequencing services were provided by Shanghai OE Biotech Co., Ltd. (Shanghai, China).

Raw sequencing reads were preprocessed using Trimmomatic to remove adapters and low-quality bases [63]. Clean reads were *de novo* assembled into transcripts using Trinity (v2.4.0) with the paired-end method [64]. The longest transcript within each cluster was selected as a unigene. Functional annotation of unigenes was performed by aligning them against several databases using DIAMOND with an e-value threshold of 1e-5 [63, 65]. These databases included: NCBI non-redundant (NR, https://www.ncbi.nlm.nih.gov/), Swiss-Prot (http://www.uniprot.org/), evolutionary genealogy of genes: Non-supervised Orthologous Groups (eggNOG, http://eggnog.embl.de/), and euKaryotic Orthologous Groups (KOG, https://ftp.ncbi.nih.gov/pub/COG/KOG/kyva)-yihe). Kyoto Encyclopedia of Genes and Genomes (KEGG) pathway annotation was carried out using the KEGG database (http://www.genome.jp/kegg/) [66]. Gene Ontology (GO) terms were assigned based on mapping from Swiss-Prot annotations.

Following annotation, clean reads from each sample were aligned to the unigene sequences using Bowtie2 (v2.4.2) [67]. Expression levels of unigenes were quantified as fragments per kilobase of transcript per million mapped fragments (FPKM) values using eXpress (v1.5.1) [68]. Differential expression analysis was performed with DESeq2 [69], applying a negative binomial test. Significantly differentially expressed genes (DEGs) were identified using a threshold of adjusted *p*-value < 0.05 and |Fold Change (FC)| > 2. Hierarchical clustering of DEGs was visualized using R language (v3.2.0). Functional enrichment analysis of GO terms and KEGG pathways was conducted based on the hypergeometric distribution, implemented in R language.

### Quantitative real-time PCR (qRT-PCR) analysis

Total RNA extraction and cDNA synthesis were conducted as described in the previous Sect.  "Transcriptome sequencing and bioinformatic analysis". Gene-specific primers for the target genes and the reference gene (18 S ribosomal RNA, 18 S rRNA [70] were designed using Primer3Plus (https://www.primer3plus.com/) and synthesized by Beijing Tsingke Biotech Co., Ltd. (Beijing, China). All primer sequences are listed in Table S1 (Additional file 3).

qRT-PCR was performed in a 20 µL reaction mixture containing 2 µL of cDNA template (500 ng), 10 µL of 2 × FastReal qPCR PreMix (SYBR Green), 0.4 µL of each forward and reverse primer (10 µM), 0.4 µL of ROX Reference Dye, and 6.8 µL of nuclease-free ddH_2_O. The reactions were carried out on a QuantStudio 1 Plus Real-Time PCR System (Thermo Fisher Scientific, Waltham, MA, USA) under the following cycling conditions: initial denaturation at 95 °C for 3 min; 40 cycles of 95 °C for 10 s, 60 °C for 10 s, and 72 °C for 20 s. Melting curve analysis was performed to confirm amplification specificity. The relative expression levels of the target genes were calculated using the 2^⁻ΔΔCt^ method [71], with normalization to the internal control gene 18 S rRNA.

### Metabolomic profiling by LC-MS and GC-MS

To investigate the impact of the endophytic *F. solani* MF20 on the metabolome of *S. vaninii* MF5, liquid chromatography-mass spectrometry (LC-MS) and gas chromatography-mass spectrometry (GC-MS) analyses were performed using a sample preparation method adapted from a report by Qi et al. [72] and Guo et al. [73]. A 60 mg sample was homogenized with two steel beads in 600 µL of methanol-water (7: 3, v/v) containing 4 µg/mL L-2-chlorophenylalanine using a grinder (60 Hz, 2 min) after pre-cooling at -40 °C for 2 min. The mixture was subjected to ultrasonic extraction in an ice-water bath for 30 min, kept at -40 °C overnight, and then centrifuged at 12,000 rpm for 10 min at 4 °C. A 150 µL aliquot of the supernatant was filtered through a 0.22 μm organic membrane and stored at -80 °C until LC-MS analysis. For GC-MS, the supernatant was transferred to a glass vial, dried under vacuum, and derivatized with 80 µL of methoxyamine hydrochloride (15 mg/mL in pyridine) at 37 °C for 60 min. Then, 50 µL of BSTFA and 20 µL of n-hexane containing 10 internal standards (C8-C24 fatty acids in chloroform) were added, and the mixture was incubated at 70 °C for 60 min. After cooling to room temperature for 30 min, samples were analyzed by GC-MS.

LC-MS analysis was conducted using an ACQUITY UPLC I-Class Plus system coupled to a Q-Exactive mass spectrometer (Waters Corporation, Milford, USA). Separation was performed on an ACQUITY UPLC HSS T3 column (100 mm × 2.1 mm, 1.8 μm) with a gradient of 0.1% formic acid in water (A) and acetonitrile (B) at a flow rate of 0.35 mL/min and column temperature of 45 °C. The gradient program was: 0–2 min, 5% B; 2–4 min, 5–50% B; 4–8 min, 50% B; 8–10 min, 50–80% B; 10–14 min, 80–100% B; 14–15 min, 100% B; 15.1–16 min, 5% B. The injection volume was 3 µL. Mass spectrometry was performed in both positive and negative ion modes with the following parameters: spray voltage, ± 3800 V; sheath gas flow, 35 Arb; auxiliary gas flow, 8 Arb; capillary temperature, 320 °C; auxiliary gas heater temperature, 350 °C; full MS resolution, 70,000; scan range, m/z 100–1200.

GC-MS analysis was carried out using an Agilent 7890B gas chromatograph coupled to an Agilent 5977 A mass spectrometer (Agilent Technologies, USA). Separation was achieved on a DB-5MS capillary column (30 m × 0.25 mm × 0.25 μm) with helium as carrier gas at 1.0 mL/min. The injector temperature was 260 °C, and 1 µL sample was injected in splitless mode. The oven temperature program was: 60 °C for 0.5 min; increased to 125 °C at 8 °C/min; to 210 °C at 8 °C/min; to 270 °C at 15 °C/min; to 305 °C at 20 °C/min; held at 305 °C for 5 min. Mass detection was performed in electron impact mode at 70 eV, with ion source temperature of 230 °C, quadrupole temperature of 150 °C, and scan range of m/z 50–500.

For data processing and statistical analysis, raw LC-MS data were processed using Progenesis QI v3.0 (Nonlinear Dynamics, UK) for peak detection, alignment, and normalization. Metabolite identification was performed using Progenesis QI v3.0 (Nonlinear Dynamics, UK) based on accurate mass and MS/MS fragmentation patterns against multiple databases, including HMDB, LipidMaps, METLIN, and an in-house database [74]. The identification criteria were as follows: (i) mass accuracy within 5 ppm (Mass error); (ii) MS/MS spectral matching score ≥ 30 out of 100 (Fragmentation score); (iii) overall identification score ≥ 36 out of 60, which integrates mass accuracy (20 points), MS/MS fragment matching (20 points), and isotopic distribution matching (20 points). Compounds with scores below these thresholds were discarded. For metabolites with MS/MS spectral matching scores ≥ 30 but without authentic standards, identification was considered putative. Metabolites with level “***" (identified from the in-house database), level “**” (matched to the KEGG database of the mapped species), or level “*” (matched to human/animal/plant databases) were retained based on the confidence level assigned by the software. Key metadata, including retention time, m/z, adduct ions, molecular formula, and KEGG IDs, were recorded for each identified metabolite. Multivariate statistical analysis (PCA, OPLS-DA) and univariate analysis (*t*-test, fold change) were performed in R language (v4.3.1) [75]. Metabolites with VIP > 1, *p* < 0.05, and |FC| > 1 were considered significantly altered [76, 77]. Pathway enrichment analysis was conducted based on KEGG using MetaboAnalyst 6.0 [78]. Integration with transcriptomic data was performed using Spearman correlation in R language, with visualization in matplotlib (v3.8.0) and NetworkX (v3.1).

### Detection of intracellular Ca^2+^ in hyphae

To assess the influence of the endophytic *F. solani* MF20 on calcium accumulation in *S. vaninii* MF5, the fluorescent calcium indicator Fluo-3 AM (Beyotime Biotechnology, Shanghai, China; Catalog No. S1056) was employed according to a previously described method with modifications [79]. Fluo-3 AM is a cell-permeable acetoxymethyl ester that is hydrolyzed by intracellular esterases to Fluo-3, which is retained within the cells and exhibits strong fluorescence upon binding to Ca^2+^, with excitation/emission maxima at 506/526 nm. Mycelial samples from both mono-cultured and co-cultured *S. vaninii* MF5 were incubated with 5 µM Fluo-3 AM at 37 °C for 1 h in the dark. Subsequently, the samples were rinsed three times with phosphate-buffered saline (PBS) to remove excess dye. The stained hyphae were mounted on glass slides and visualized under a fluorescence microscope (Yongxin Optics Co., Ltd., Ningbo, China).

### Measurement of ATP content

To investigate the effect of *F. solani* MF20 on the ATP content of *S. vaninii* MF5, fresh mycelial samples were collected after 10 days of co-culture. The ATP concentration was measured using an ATP Assay Kit (Beyotime Biotechnology, Shanghai, China; Catalog No. S0026) according to the manufacturer’s instructions and a previously described method [80]. After the reaction, the ATP content was quantified using a Spark™ multimode microplate reader (Tecan, Männedorf, Switzerland).

### Assessment of redox status

To analyze the effect of the endophytic *F. solani* MF20 on reactive oxygen species (ROS) burst in *S. vaninii* MF5, the intracellular ROS level was detected using a ROS Assay Kit (Beyotime Biotechnology, Shanghai, China; Catalog No. S0119). Fresh mycelial samples were collected after 10 days of co-culture and incubated with 10 µM DCFH-DA fluorescent probe [81] at 37 °C for 1 h. The samples were then rinsed three times with distilled water to remove unbound dye. The stained hyphae were mounted and observed under a fluorescence microscope (Yongxin Optics Co., Ltd., Ningbo, China) with excitation/emission wavelengths of 485/528 nm. Hydrogen peroxide (H_2_O_2_) content was measured using the potassium iodide method [82]. Fresh mycelia (0.1 g) were homogenized in liquid nitrogen and extracted with 1.5 mL of 0.1% (w/v) trichloroacetic acid (TCA). The homogenate was centrifuged at 12,000 rpm for 15 min at 4 °C. Then, 0.5 mL of the supernatant was mixed with 0.5 mL of 10 mM PBS (pH 7.0) and 1 mL of potassium iodide (KI) solution. After incubation at 28 °C for 1 h, the absorbance was measured at 390 nm using a UV-2800 spectrophotometer (Unico Instrument Co., Ltd., Shanghai, China). Superoxide anion (O_2_^−^ ) content was determined by the hydroxylamine hydrochloride oxidation method [83]. Samples were processed as described above. Briefly, 0.1 g of mycelia was extracted with 1 mL of PBS and centrifuged at 5000 ×*g* for 15 min at 4 °C. Then, 0.5 mL of the supernatant was mixed with 0.5 mL of PBS and 1 mL of hydroxylamine hydrochloride, followed by incubation at 25 °C for 1 h. Thereafter, 1 mL of sulfanilic acid and 1 mL of α-naphthylamine were added, and the mixture was incubated at 25 °C for 20 min. The absorbance was measured at 530 nm.

The activities of antioxidant enzymes and the content of reduced glutathione (GSH) were measured using commercial assay kits according to the manufacturers’ instructions. The extracted samples were reacted with respective reagents from the following kits: Catalase (CAT) Activity Assay Kit (Addison Biotechnology Co., Ltd., China; ADS-W-KY002), Peroxidase (POD) Activity Assay Kit (Solarbio, Beijing, China; BC0095), Thioredoxin Reductase (TrxR) Activity Assay Kit (Solarbio, Beijing, China; BC1105), Glutathione Peroxidase (GSH-Px) Assay Kit (Beyotime Biotechnology, Shanghai, China; S0056), and Reduced Glutathione (GSH) Content Assay Kit (Addison Biotechnology Co., Ltd., China; ADS-W-G001). The absorbance was measured using a Spark™ multimode microplate reader (Tecan, Männedorf, Switzerland). Enzyme activities and GSH content were calculated based on standard curves.

### Statistics analysis

All experiments were performed with three independent biological replicates, except for omics sequencing experiments, which included both control and treatment groups with six biological replicates per group. For metabolite quantification (flavonoids, terpenoids, and polysaccharides), three independent biological replicates were used, each measured in triplicate technical replicates. For fluorescence intensity quantification (SYTOX Green, Fluo-3 AM, DCFH-DA), at least three random microscopic fields were analyzed per sample from three independent biological replicates. Data were analyzed using SPSS statistical software (version 26.0) and subjected to *t*-test or one-way analysis of variance (ANOVA) followed by post-hoc tests (Least Significant Difference (LSD) or Duncan’s multiple range test) where appropriate. Differences were considered statistically significant at *p* < 0.05. Graph generation and further statistical analyses were performed using GraphPad Prism (version 9.4.1) and Microsoft Excel. Data are presented as mean ± standard deviation (SD).

## Results and discussion

### Growth and metabolite production of *S. vaninii* in solid-state fermentation

The colony of *S. vaninii* MF5 exhibited rapid growth during the first 6 days following inoculation, accompanied by the production of abundant pale-yellow aerial mycelia. Pigment accumulation initiated around day 10. By day 14, the mycelia had fully colonized the plate, with continued expansion of aerial mycelia and a gradual color shift to light yellowish-brown (Fig. S1a; Additional file 1).

The synthesis of terpenoids (0.14–0.18 mg/g FW) and flavonoids (0.04–0.05 mg/g FW) remained at low levels within the first 8 days of cultivation. However, their production increased markedly between days 12 and 16, reaching 0.88 mg/g FW and 0.54 mg/g FW, respectively, before declining and stabilizing by day 18 (Fig. S1b; Additional file 1). The accumulation dynamics of these two major classes of secondary metabolites showed a highly synchronized pattern, both peaking between days 12 and 16. This trend aligns with the classic physiological shift from primary growth to secondary metabolism in fungal life cycles, where the depletion of readily available nutrients often triggers the biosynthesis of specialized metabolites [84, 85]. In contrast, polysaccharide content displayed a distinct biphasic accumulation pattern (Fig. S1c; Additional file 1). It reached a first peak of 56.89 mg/g DW (dry weight) on day 8, subsequently declined, and then showed a secondary increase to 28.33 mg/g DW on day 16. This biphasic pattern likely reflects complex and highly regulated carbon allocation within the fungal metabolic network. The initial peak may be closely associated with rapid mycelial biomass expansion and cell wall construction, where polysaccharides are synthesized as key structural components and carbon reserves [86]. The secondary rise could indicate a phase of cell wall remodeling or reinforcement, potentially in preparation for differentiation or environmental stress responses [87]. The subsequent decline suggests that these polysaccharides are not static end-products but are dynamically metabolized - possibly serving as carbon and energy sources for other pathways (e.g., secondary metabolism) or being further modified into specialized cell wall structures [88]. This dynamic highlight the multifunctional role of polysaccharide metabolism throughout the fungal lifecycle. Therefore, the 16-day time point, representing the peak accumulation phase for these bioactive compounds, was selected for subsequent experimental sampling to maximize the yield of target metabolites.

### Enhanced metabolite production in *S. vaninii* through co-culture with *F. solani*

Building upon the predicted trophic modes (e.g., Pathotroph, Saprotroph, Symbiotroph) of endophytes in *S. vaninii* at the family level (Fig. 2a) [45], we isolated 37 endophytic fungal strains from surface-sterilized fruiting bodies (Fig. S2a; Additional file 1). To assess their potential to influence the host, a dual-culture screening system was established between *S. vaninii* MF5 and each isolate (Fig. 2b). Co-culture with strain MF20 induced a distinct phenotypic shift in the host, characterized by reduced aerial hyphal density and a pronounced darkening of colony pigmentation to a deep yellow-brown, especially at the interaction zone (Fig. 2c). This altered morphology, particularly the enhanced pigmentation, is a classic indicator of a fungal stress response, often signifying the activation of chemical defense mechanisms and a metabolic reallocation from growth to secondary metabolism [89]. Metabolite analysis confirmed this physiological shift. Co-culture with MF20 significantly boosted the accumulation of key bioactive compounds in *S. vaninii* MF5: total flavonoid content increased 2.93-fold to 0.67 mg/g FW, and crude polysaccharide content rose 2.23-fold to 65.40 mg/g DW, while terpenoid content showed a modest increase to 0.96 mg/g FW (Fig. 2d). The marked enhancement of flavonoids and polysaccharides identifies MF20 as a potent biotic elicitor. This aligns with the OSMAC principle, wherein co-culture serves as an effective strategy to perturb metabolic homeostasis and activate biosynthetic potential [20]. Notably, our strategy employs a native endophyte rather than an ecologically unrelated fungus. While co-culture between taxonomically distant fungi (e.g., *Aspergillus* spp., *Funalia* with *Penicillium*) can stimulate metabolite production [21, 22, 25], the interaction between *S. vaninii* MF5 and its resident endophyte MF20 likely engages a pre-evolved, specific molecular dialogue. This native elicitor approach may trigger a more efficient and targeted host response, explaining the strong metabolite induction observed.

rescence, indicating significantly enhancedmembrane permeability, (Fig. 7a, right panel). Quantitative analysis co

The endophytic strain MF20 was molecularly identified by sequencing the ITS rDNA region (GenBank No. OR672754). Phylogenetic analysis revealed 99% similarity to *F. solani* (Fig. S2b; Additional file 1), leading to its designation as *F. solani* MF20. The capacity of MF20 to act as an effective elicitor is consistent with the recognized metabolic versatility of the *Fusarium* genus, which harbors a rich repertoire of biosynthetic gene clusters and is known for prolific secondary metabolite production [90, 91]. Critically, the functional role of *Fusarium* in cross-kingdom interactions is highly context-dependent. For instance, *Fusarium* mycotoxins can be degraded by specific metabolites from *Trichoderma* in co-culture [92], while conversely, a *Fusarium* sp. SF12 isolated from *Shiraia* can enhance the production of the host’s own photosensitizer [93]. Our study delineates a novel and applicable mutualistic paradigm by demonstrating that the native endophyte *F. solani* MF20 interacts with its host *S. vaninii* not as a pathogen but as a potent and beneficial elicitor, significantly boosting the production of pharmaceutically relevant metabolites.

### Inoculation timing is critical for maximizing metabolite yields

**Fig. 3:**
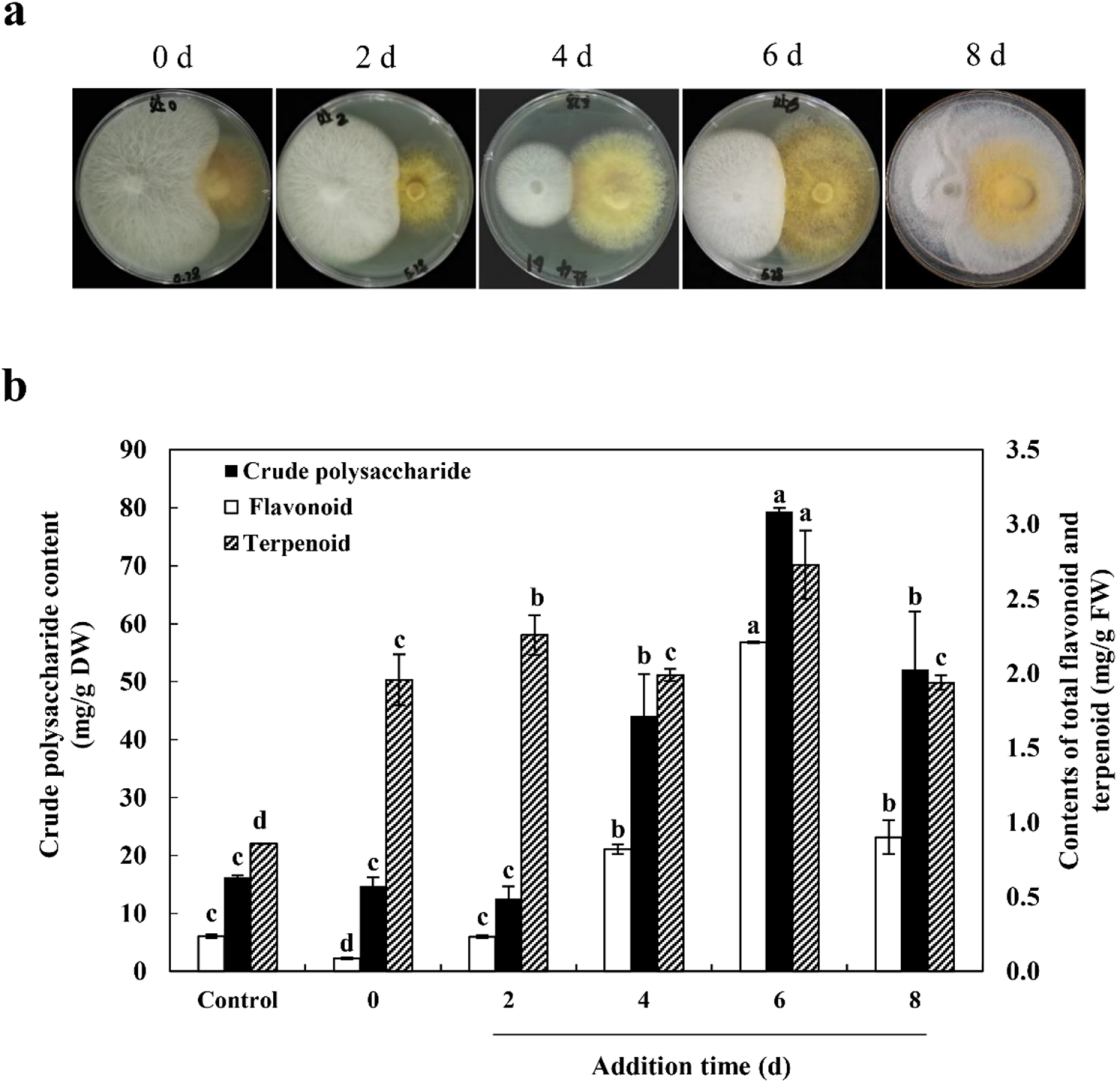
Optimization of the co-culture system: inoculation timing is critical for metabolite enhancement. **a** Growth dynamics of *S. vaninii* MF5, assessed by visual observation of colony expansion, when *F. solani* MF20 was introduced at different time points (0, 2, 4, 6, 8 days) during host fermentation. **b** Production of total flavonoids, terpenoids, and crude polysaccharides in MF5 mycelia sampled from the host colony region after 10 days of co-culture. Data are presented as mean ± SD from three independent biological replicates (*n* = 3). Different *letters* above bars indicate significant differences (*p* < 0.05) as determined by one-way ANOVA followed by Duncan’s multiple range test

To optimize the co-culture process, *F. solani* MF20 was inoculated at different time points during the solid-state fermentation of *S. vaninii* MF5. Phenotypic observation revealed a clear optimal window (Fig. 3a). Due to its faster growth rate, inoculation of MF20 within the first 5 days led to competitive inhibition of the host, reducing MF5 colony expansion. Conversely, inoculation after day 8 resulted in the suppression of MF20 by the established host culture. Consequently, day 6 was identified as the optimal inoculation time point. Metabolite analysis (Fig. 3b) further supported this observation. When MF20 was introduced during the early stages (days 0–2), the flavonoid content and crude polysaccharide content were lower than those of the axenic control. Terpenoid content was also generally lower than that of the control. As anticipated from the phenotypic observations (Fig. 3a), inoculation with MF20 on day 6 - followed by 10 days of co-culture (16 days total fermentation) - significantly enhanced metabolite production. The flavonoid content reached 2.21 mg/g FW, 9.38 times that of the control; crude polysaccharides increased to 79.34 mg/g DW, 4.87 times the control level; and terpenoids peaked at 2.73 mg/g FW, 3.18 times higher than the control. Later inoculation (after day 8) resulted in reduced terpenoid (1.94 mg/g FW) and crude polysaccharide (52.11 mg/g DW) content, while flavonoid levels (2.21 mg/g FW) showed no significant difference from the control.

The identification of this precise optimal window is not merely about yield maximization; it also reveals the critical ecological dynamics governing inter-fungal interactions. Inoculation too early (e.g., day 2) likely triggers intense nutrient and spatial competition before the host establishes robust mycelial growth (as indicated by colony expansion), leading to growth inhibition rather than productive metabolic elicitation [94]. Inoculation too late (e.g., after day 8) occurs when the host mycelium is mature and its metabolic network has lower plasticity, rendering it less responsive to biotic signals [95]. Therefore, day 6 represents a key ecological “sweet spot”: the host is sufficiently established to dominate its niche, yet its physiology remains in a highly flexible, resource-rich state, poised to redirect resources from growth to defense-associated secondary metabolism upon sensing a biotic challenge. Similar timing-dependent effects have been noted in other systems, such as bacterial-fungal co-culture for perylenequinone drug production of *Shiraia* [96].

### Transcriptomic regulation of *S. vaninii* by the endophytic *F. solani*

To elucidate the molecular mechanisms by which *F. solani* MF20 influences its host, a comparative transcriptomic analysis was conducted between axenic *S. vaninii* MF5 (control, CK) and co-culture under optimized conditions (experimental group, EG). Twelve cDNA libraries were sequenced, generating 86.16 Gb of high-quality clean data (Table S2; Additional file 2). *De novo* assembly produced 25,373 unigenes with an average length of 1,470.87 bp, which were functionally annotated across multiple databases (Table S3; Additional file 2). The data have been deposited in the NCBI GEO under accession No. GSE272365. PCA revealed a clear separation between control (CK) and co-culture (EG) groups along PC1, which explained 94.75% of the variance, indicating a profound and reproducible global reprogramming of the host transcriptome induced by co-culture (EG) (Fig. S3a; Additional file 1). A total of 11,892 DEGs were identified (|log_2_FC| ≥ 1, *p* < 0.05), with 9,558 up-regulated and 2,334 down-regulated genes in the co-culture (EG) group (Fig. S3b; Additional file 1) (Table S4; Additional file 3). The sheer scale of this transcriptional shift, dominated by up-regulation, signifies that the host mounted a systemic, transcriptome-wide response to the endophyte, far beyond the activation of isolated pathways [97]. The reliability of the RNA-seq data was validated by qRT-PCR on 42 randomly selected genes from enriched pathways (Fig. S4; Additional file 1).

#### GO enrichment analysis reveals a shift to stress-adaptive physiology

GO enrichment analysis highlighted significant changes across biological processes (BP), molecular functions (MF), and cellular components (CC) (Fig. S5; Additional file 1) (Table S5; Additional file 3). In the BP category, up-regulated DEGs were highly enriched in translation (GO:0006412), protein folding (GO:0006457), and the proteasomal catabolic process (GO:0010498) (Fig. S5a; Additional file 1). This concerted up-regulation of protein synthesis, folding, and degradation machineries indicates a massive turnover and synthesis of new proteins, a hallmark of cellular adaptation to stress and metabolic rewiring [98, 99]. The enrichment of response to oxidative stress (GO:0006979) and arginine biosynthesis (GO:0006526) further confirms the activation of classic defense and signaling programs, where arginine may serve as a precursor for stress-related molecules like polyamines or nitric oxide [100, 101]. Conversely, down-regulated BP terms included transmembrane transport (GO:0055085), DNA replication (GO:0006260), and DNA repair (GO:0006281). This suggests a strategic reallocation of cellular resources away from growth and homeostasis maintenance toward defense and specialized metabolism [102]. In the MF category (Fig. S5b; Additional file 1), the up-regulation of ribosome-related and metal ion binding terms (GO:0046872) supports the need for enhanced protein synthesis capacity [103]. The down-regulation of heme/iron binding and monooxygenase activity may indicate a strategic redirection of secondary metabolic fluxes [104, 105]. Enrichment in CC category (Fig. S5c; Additional file 1) reinforced this picture, with up-regulation of ribosomal terms and down-regulation of plasma membrane-associated functions, consistent with a shift in focus from nutrient acquisition to internal biosynthesis and adaptation [106].

#### KEGG pathway analysis unveils coordinated metabolic rewiring

**Fig. 4:**
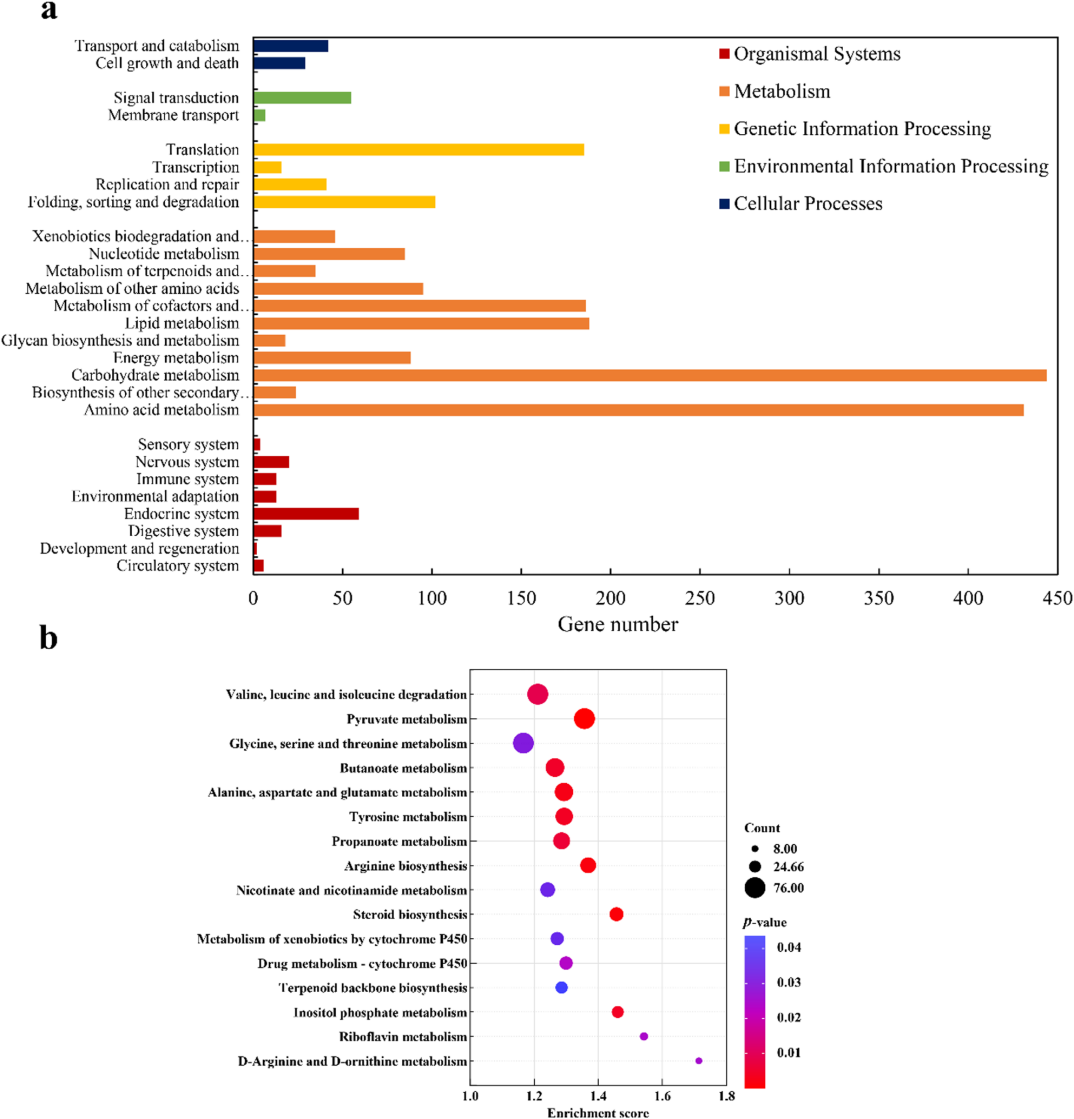
Functional enrichment analysis of the transcriptomic response in *S. vaninii* MF5 induced by co-culture with *F. solani* MF20. **a** Classification and gene count of DEGs in Level 2 KEGG pathways. **b** Bubble plot of significantly enriched KEGG pathways. DEGs were defined as |log_2_FC| ≥ 1 and adjusted *p* < 0.05 (DESeq2). Enrichment analysis was performed based on the hypergeometric distribution. CK, axenic control (*n* = 6 biological replicates); EG, co-culture group (*n* = 6 biological replicates)

KEGG enrichment analysis provided a systems-level view of the metabolic reorganization (Table S6; Additional file 3) (Fig. 4). At the classification level, DEGs were predominantly associated with Metabolism (e.g., carbohydrate, amino acid) and Genetic Information Processing (Fig. 4a). Pathway-based analysis revealed the most significant enrichment in central carbon and amino acid catabolic pathways, including pyruvate metabolism (ko00620), valine, leucine and isoleucine degradation (ko00280), and glycine, serine and threonine metabolism (ko00260) (Fig. 4b). This widespread mobilization of central carbon and nitrogen metabolism likely meets the increased energy (ATP) and precursor demands of the host’s adaptive response [107]. Critically, several pathways directly linked to secondary metabolite biosynthesis were significantly up-regulated. These included terpenoid backbone biosynthesis (ko00900), steroid biosynthesis (ko00100), and drug metabolism - cytochrome P450 (ko00982). The induction of these pathways provides a direct molecular explanation for the observed boost in terpenoid and flavonoid production, signifying the activation of the host’s chemical defense machinery [108, 109]. The co-enrichment of nicotinate and nicotinamide metabolism (ko00760) points to an increased demand for redox cofactors (NAD/NADH) to support this biosynthetic burst [110], while arginine biosynthesis (ko00220) and inositol phosphate metabolism (ko00562) underscore the involvement of specialized nitrogen metabolism and signaling networks in this interplay [111]. The mild enrichment observed in the riboflavin metabolism pathway (ko00740) further suggests an increased demand for flavin cofactors (e.g., FAD, FMN), which are essential for numerous oxidoreductases involved in secondary metabolism and stress response [112, 113].

#### Identification of co-culture specific genes

**Fig. 5:**
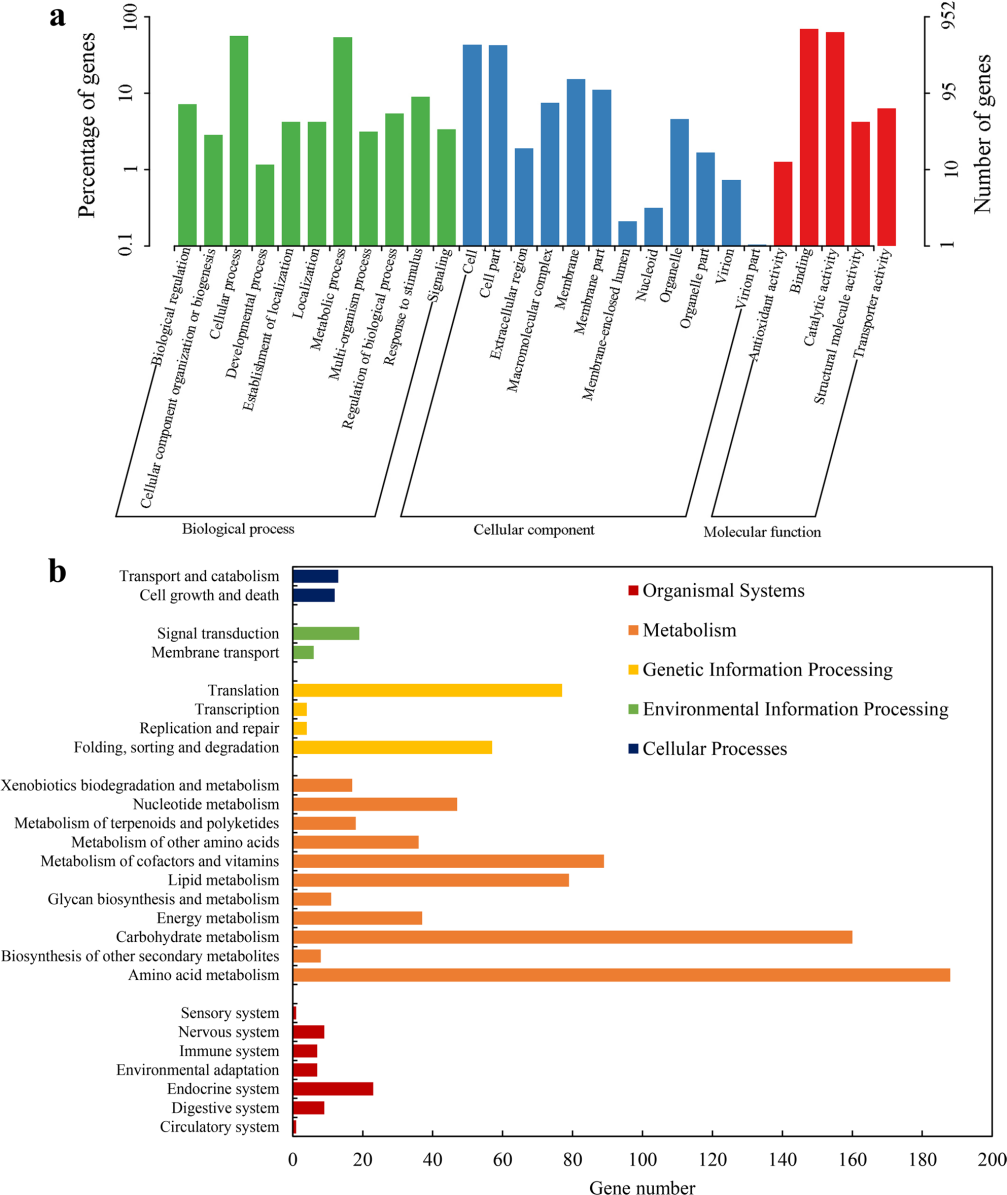
Functional profiling of genes exclusively activated in *S. vaninii* MF5 during co-culture with *F. solani* MF20. **a** GO enrichment analysis of genes with infinite fold-change (expressed only in co-culture). **b** KEGG pathway enrichment analysis of the co-culture-specific genes. Exclusive genes were defined as those detected in all six replicates of the co-culture group (EG) but not in any replicate of the control group (CK). Enrichment analysis was performed using the hypergeometric distribution with a significance threshold of adjusted *p* < 0.05

A distinct subset of genes was exclusively expressed (infinite-fold change, Inf) in the co-culture system (Table S7; Additional file 3). GO analysis of these genes showed enrichment for cellular process, metabolic process, response to stimulus, catalytic activity, and membrane components (Fig. 5a). This profile suggests the *de novo* activation of a genetic program for processing interaction-specific signals and executing related metabolic functions [114, 115]. KEGG analysis of these exclusive genes showed the highest representation in amino acid and carbohydrate metabolism, and metabolism of cofactors and vitamins (Fig. 5b). This aligns with the overall metabolic rewiring but suggests these genes may support the synthesis of unique interaction metabolites not required in axenic culture [116]. Their enrichment in signal transduction and membrane transport pathways hints that they may encode key components for perceiving the endophytic signal and initiating the early host response [115]. The activation of a suite of co-culture- specific genes advances our understanding from a global reprogramming to the activation of a specific symbiotic response program. These genes represent a potential dedicated toolkit for this interaction, underscoring the distinctive intensity of genetic reprogramming possible in co-evolved endophyte-host systems [117].

Conversely, 21 genes were silenced in co-culture and expressed only in the axenic control (Table S7; Additional file 3). These included genes encoding a helicase, an RNA-binding protein, a component implicated in exocytosis, a Sel1 domain repeat-containing protein, and a fungal-specific transcription factor. The silencing of these genes in co-culture may indicate a strategic down-regulation of certain host-specific regulatory and maintenance networks to accommodate or respond to the symbiotic partner: the exocytosis-related gene hints at a modulation of secretory pathways during interaction [118], while the Sel1 domain protein, involved in protein quality control [119], suggests adjustments to cellular homeostasis. The silencing of host-specific transcription factor further indicates a strategic down-regulation of certain host-specific regulatory and maintenance networks to accommodate or respond to the symbiotic partner [120–122].

#### Transcriptomic analysis of F. solani-mediated growth regulation in S. vanini

**Fig. 6:**
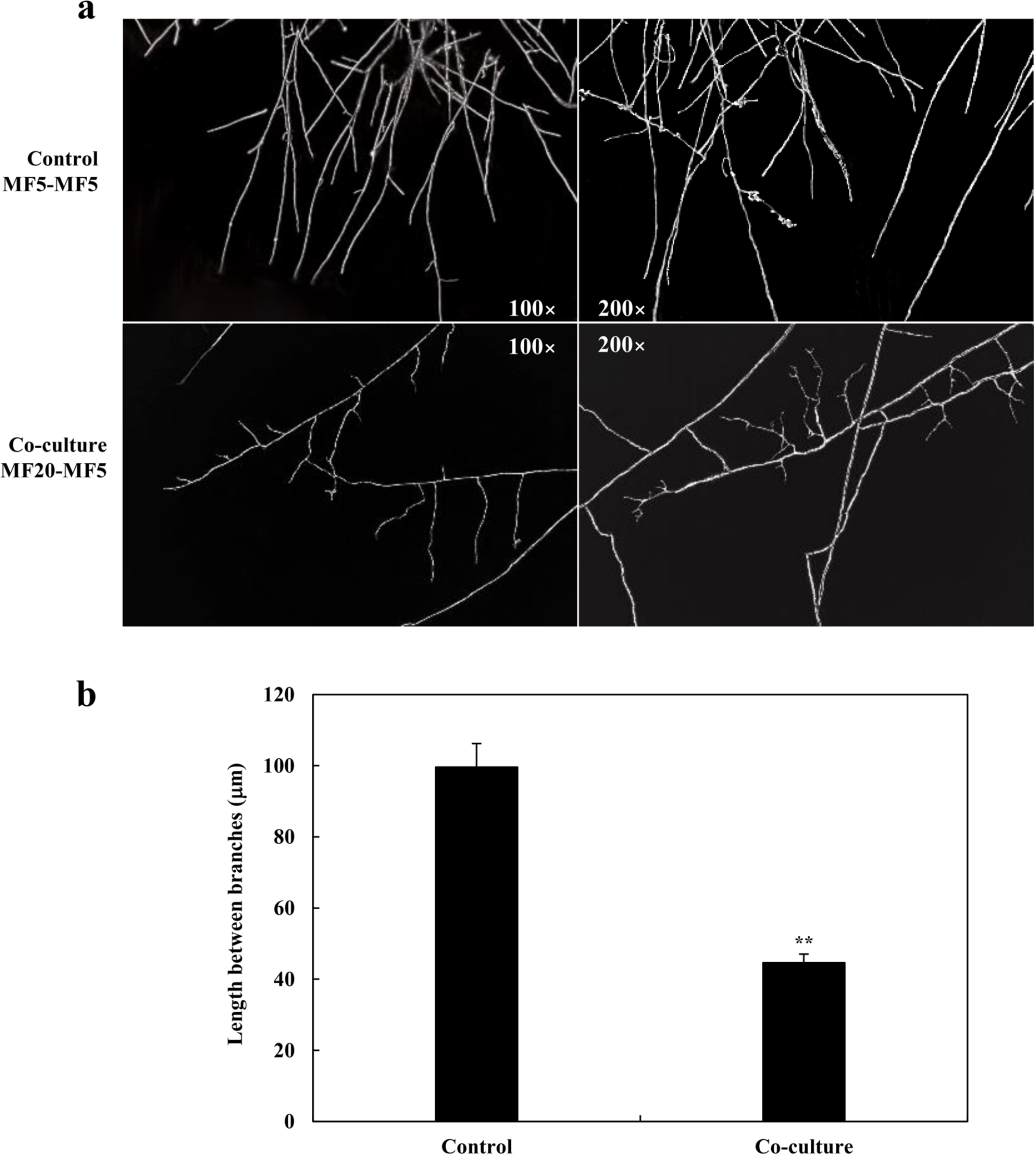
Co-culture with *F. solani* MF20 alters hyphal morphology of *S. vaninii* MF5. **a** Representative light microscopy images of MF5 hyphae after 10 days of monoculture or co-culture with MF20. **b** Quantification of the average inter-branch distance. For each sample, at least 30 hyphal segments from three random microscopic fields were measured using ImageJ software. Data are presented as mean ± SD from three independent biological replicates (*n* = 3). Statistical significance was determined by Student’s *t*-test (**p < 0.01 vs. monoculture control)

To investigate how the endophyte *F. solani* MF20 influences host development, we first examined hyphal morphology. Co-culture significantly increased hyphal branching in *S. vaninii* MF5 (Fig. 6a), reducing the average inter-branch distance from 99.61 μm to 44.63 μm (Fig. 6b). This morphological shift was underpinned by the pronounced up-regulation of genes governing hyphal growth and morphogenesis (Table S8; Additional file 2). Key upregulated regulators included multiple Rho-type small GTPases and serine/threonine protein kinases, which are central to cytoskeletal reorganization and polarity establishment in fungi [73, 123–125]. Notably, several of these kinases were exclusively expressed (Inf, Table S8; Additional file 2) in co-culture. Furthermore, genes encoding cell wall-modifying enzymes such as glycosyl hydrolases and chitinases [126, 127], as well as regulators of gene expression like a histone acetyltransferase [128] and ribosomal protein S6 kinase [129], were also up-regulated, collectively promoting a transcriptional and physiological state conducive to enhanced branching and growth. The increase in hyphal branching has direct physiological implications. By expanding the biomass surface area, the host likely enhances its capacity for nutrient uptake and metabolite exchange, potentially contributing to the observed boost in biomass and compound production [130].

Beyond morphology, GO enrichment analysis indicated that co-culture significantly affected processes and components related to the plasma membrane (e.g., GO:0005886, GO:0016021) and fatty acid metabolism (GO:0006631) in the host (Fig. S5; Additional file 1). Transcriptomic data revealed a marked shift in lipid metabolism, characterized by the strong up-regulation (575.78-fold) of a Δ12-fatty-acid desaturase, a key enzyme in unsaturated fatty acid (UFA) synthesis, alongside the down-regulation of genes involved in fatty acid degradation. Since UFAs increase membrane fluidity, this change in lipid composition likely alters the physical properties of the host membrane [131]. Consistent with this, we observed differential expression of a broad suite of genes encoding membrane transport proteins, including ABC transporters, amino acid permeases, and major facilitator superfamily (MFS) permeases (Table S9; Additional file 2). This comprehensive reprogramming of the transportome, together with altered membrane lipid composition, suggests a targeted modulation of membrane permeability. In the context of this controlled, mutualistic interaction, increased permeability may function not as detrimental damage but as a biological process accelerator. It could facilitate the more efficient exchange of interspecies signals, nutrient precursors, and final metabolic products across the symbiotic interface [132]. Critically, enhanced efflux of target compounds like terpenoids and flavonoids could alleviate end-product feedback inhibition, allowing their biosynthetic pathways to operate at a continuously high flux [133]. This adaptive model is supported by similar observations where modulated membrane permeability promotes metabolite synthesis in fungi [70].

**Fig. 7:**
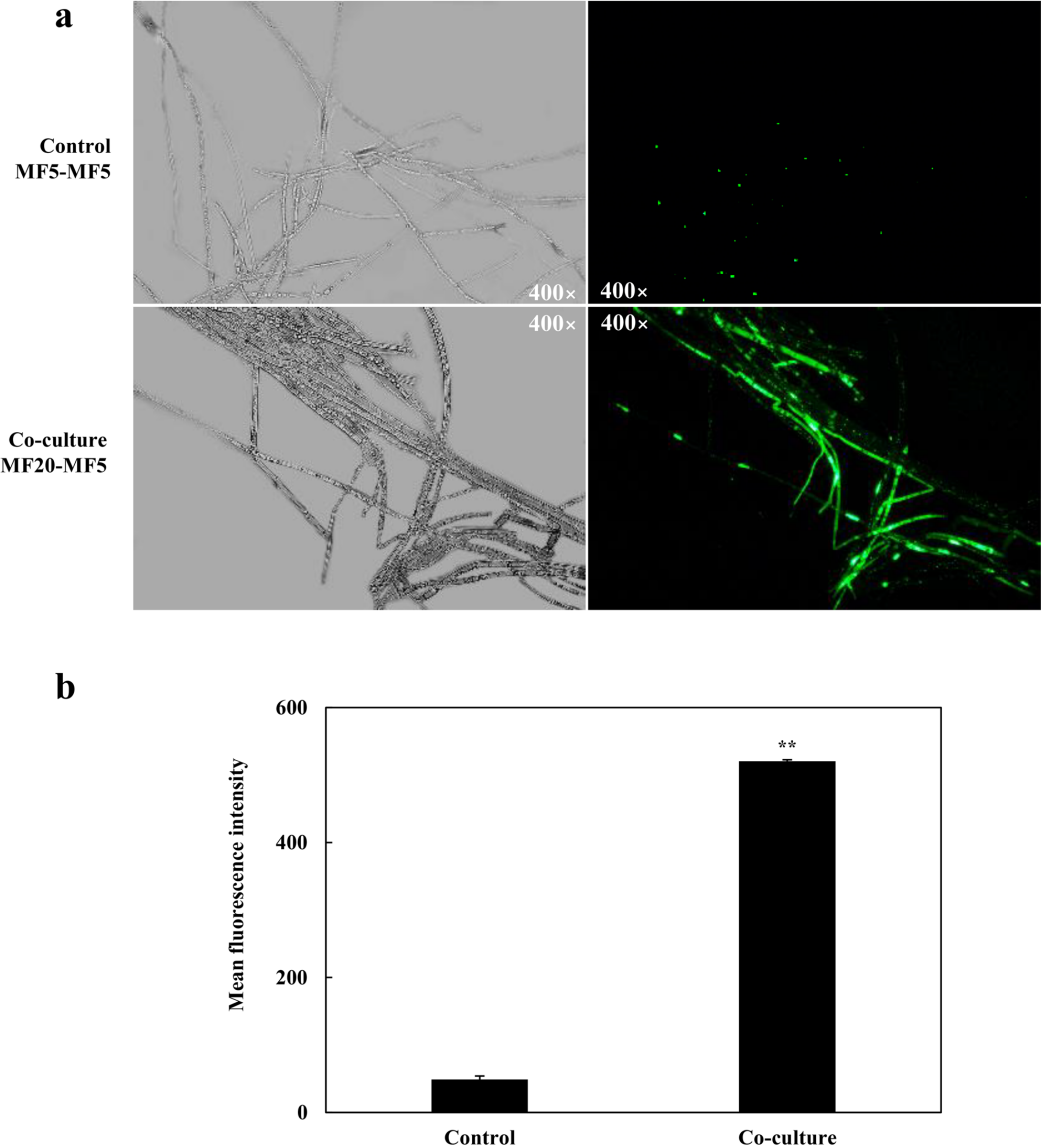
Co-culture with *F. solani* MF20 increases membrane permeability in *S. vaninii* MF5. **a** Representative fluorescence microscopy images of MF5 hyphae stained with SYTOX Green after 10 days of monoculture or co-culture. Mycelia were incubated with 0.5 µM SYTOX Green for 10 min in the dark. **b** Quantitative analysis of SYTOX Green fluorescence intensity. Images were captured under identical exposure settings from at least three random microscopic fields per sample. Fluorescence intensity was measured using ImageJ software by analyzing the mean gray value of stained hyphal regions after background subtraction. Data are presented as mean ± SD from three independent biological replicates (*n* = 3). Statistical significance was determined by Student’s *t*-test (**p < 0.01 vs. monoculture control). Hence, to functionally assess membrane integrity, we employed SYTOX Green staining. While hyphal density appeared similar between groups, (Fig. 7a, left panel), hyphae from co-culture exhibited intense fluorescence, indicating significantly enhanced membrane permeability, (Fig. 7a, right panel). Quantitative analysis confirmed a 368.83-fold increase in fluorescence intensity compared to the axenic control, (Fig. 7b). This result validates the transcriptomic predictions and supports the interpretation that the altered membrane state is a regulated, adaptive response within the symbiotic partnership, likely facilitating the enhanced metabolic exchange and production observed [70]

### Transcriptomic analysis of stress responses in *S. vaninii* induced by *F. solani*

#### Altered ion transport and energy metabolism

**Fig. 8:**
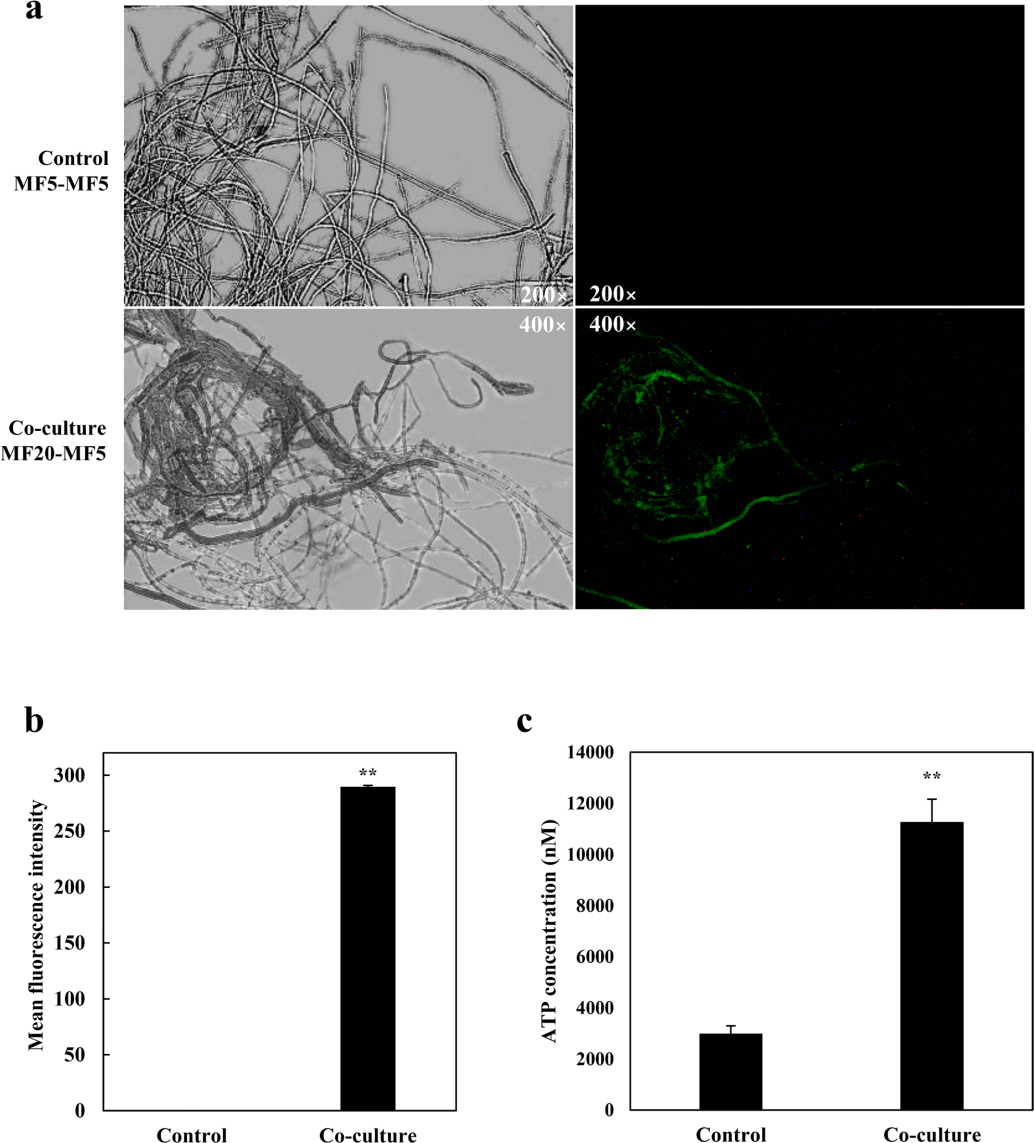
Co-culture with *F. solani* MF20 triggers Ca^2+^ influx and ATP accumulation in *S. vaninii* MF5. **a** Representative fluorescence microscopy images of MF5 hyphae stained with Fluo-3 AM after 10 days of monoculture or co-culture. Mycelia were incubated with 5 µM Fluo-3 AM at 37 °C for 1 h in the dark. **b** Quantification of Fluo-3 AM fluorescence intensity, reflecting intracellular Ca^2+^ levels. Images were captured under identical exposure settings from at least three random microscopic fields per sample. Fluorescence intensity was measured using ImageJ software. **c** ATP content measured using an ATP Assay Kit. Data are presented as mean ± SD from three independent biological replicates (*n* = 3). Statistical significance was determined by Student’s *t*-test (**p < 0.01 vs. monoculture control)

Exposure to *F. solani* MF20 triggered significant transcriptional changes in *S. vaninii* MF5 related to ion transport and energy metabolism. GO enrichment analysis showed significant up-regulation of terms including proton-transporting ATP synthase activity (GO:0045261), ATP synthesis coupled proton transport (GO:0015986), calcium-transporting ATPase activity (GO:0005388), and calcium ion transmembrane transport (GO:0070588) (Fig. S5; Additional file 1). This was supported by the up-regulation of specific genes encoding F_0_F_1_-type ATP synthases, calcium-transporting ATPases, calcium-dependent protein kinases, and sodium/calcium exchangers (Table S10; Additional file 2). Functional assays confirmed these molecular changes. Intracellular Ca^2+^ levels, measured via Fluo-3 AM staining, were 289.79-fold higher in co-cultured hyphae compared to the axenic control (Fig. 8a, b). Concurrently, cellular ATP levels increased 3.77-fold in the co-culture system (Fig. 8c).

These coordinated changes represent a classic, early signaling cascade. The rapid influx of Ca^2+^, a ubiquitous secondary messenger in fungi, signifies the host’s recognition of the endophytic partner [134]. The role of Ca^2+^ in transducing external cues into metabolic reprogramming is well-established in medicinal fungi; for instance, it is a critical regulator of ganoderic acid biosynthesis in *Ganoderma lucidum* under various stresses [135, 136]. The concurrent surge in ATP provides the immediate energy currency required to fuel subsequent energy-intensive adaptations, such as cytoskeletal remodeling and enhanced biosynthesis [80, 137]. The up-regulation of proton-pumping ATPases and Ca^2+^/H^+^ exchangers further indicates a homeostatic effort to fine-tune membrane potential and modulate the amplitude/duration of the Ca^2+^ signal [138, 139]. Thus, the triad of Ca^2+^ signaling, ATP synthesis, and proton pump activation forms a precise early-response module, priming the host for large-scale morphological and metabolic reprogramming.

#### Activation of oxidative stress and defense responses

**Fig. 9:**
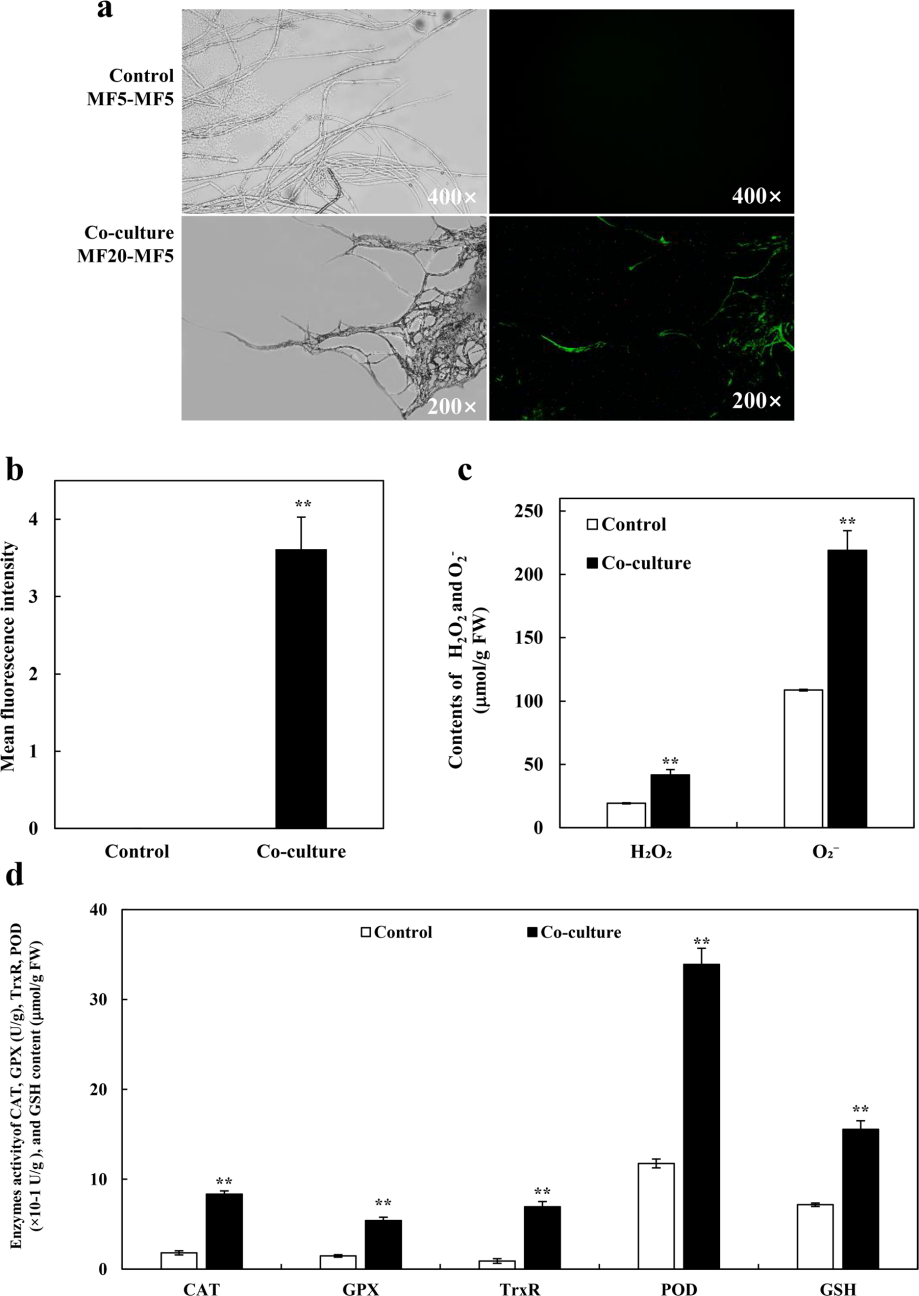
Co-culture with *F. solani* MF20 induces a controlled oxidative stress response in *S. vaninii* MF5. **a** Representative fluorescence microscopy images of MF5 hyphae stained with DCFH-DA after 10 days of monoculture or co-culture. Mycelia were incubated with 10 µM DCFH-DA at 37 °C for 1 h. **b** Quantification of DCFH-DA fluorescence intensity. Images were captured under identical exposure settings from at least three random microscopic fields per sample. Fluorescence intensity was measured using ImageJ software. **c** H_2_O_2_ and O_2_^−^. contents measured by spectrophotometric methods. **d** Activities of antioxidant enzymes (CAT, GSH-Px, TrxR, POD) and content of reduced glutathione (GSH) measured using commercial assay kits. Data are presented as mean ± SD from three independent biological replicates (*n* = 3). Statistical significance was determined by Student’s *t*-test (*p < 0.05, **p < 0.01 vs. monoculture control)

Co-culture with MF20 also induced a robust oxidative stress response. Transcriptomic data revealed significant enrichment of GO terms such as oxidoreductase activity (GO:0016491), hydrogen peroxide catabolic process (GO:0042744), and response to oxidative stress (GO:0006979) (Fig. S5; Additional file 1). Key up-regulated genes included those for glutathione peroxidase, catalase-peroxidase, and alkyl hydroperoxide reductase (Table S11; Additional file 2). The activation of intracellular signal transduction pathways (GO:0035556) was evidenced by the up-regulation of genes encoding mitogen-activated protein kinase kinase kinase, serine/threonine protein kinases, and cAMP-dependent protein kinase. Physiological measurements validated this transcriptional shift. DCFH-DA staining showed a 3.61-fold increase in general ROS levels in co-cultured hyphae (Fig. 9a, b). Specific assays confirmed that H_2_O_2_ and O_2_^−^ levels increased by 2.17- and 2.01-fold, respectively (Fig. 9c). Crucially, antioxidant defenses were coordinately enhanced: activities of CAT, TrxR, and POD increased by 4.61-, 7.66-, and 2.88-fold, respectively (Fig. 9d). Reduced GSH content and GSH-Px activity were also significantly elevated.

The synchronized burst of ROS, along with the up-regulation of antioxidant machinery, defines a state of controlled oxidative stress. Rather than indicating indiscriminate damage, this response represents a finely-tuned redox signaling event. In fungal systems, such regulated ROS bursts are known to be associated with the reallocation of cellular resources from growth to the biosynthesis of defense-associated secondary metabolites. This principle is clearly demonstrated in other fungi: in *F. graminearum*, ROS generation is integral to the induction of trichothecene mycotoxin synthesis [140]; and in *S. bambusicola*, a deliberate ROS burst is a critical signal for upregulating photosensitizer hypocrellin A biosynthesis [37]. The parallel activation of key signaling kinases (e.g., MAPK, PKA-cAMP-PKA) strongly suggests that the ROS generated here may function as signaling molecules involved in transducing the elicitation signal [134, 141]. Therefore, we interpret endophyte-induced oxidative stress as a signal that may trigger the host’s shift from primary growth to defense mode, ultimately contributing to enhanced synthesis of target bioactive compounds.

### Metabolomic analysis reveals *F. solani*-induced alterations in *S. vaninii*

To comprehensively assess the metabolic impact of the endophyte *F. solani* MF20, we performed untargeted metabolomic profiling of *S. vaninii* MF5 using both LC-MS and GC-MS platforms. The data have been deposited in the GSA database under accession No. OMIX007469.

#### Global metabolic reprogramming and pathway alterations

**Fig. 10:**
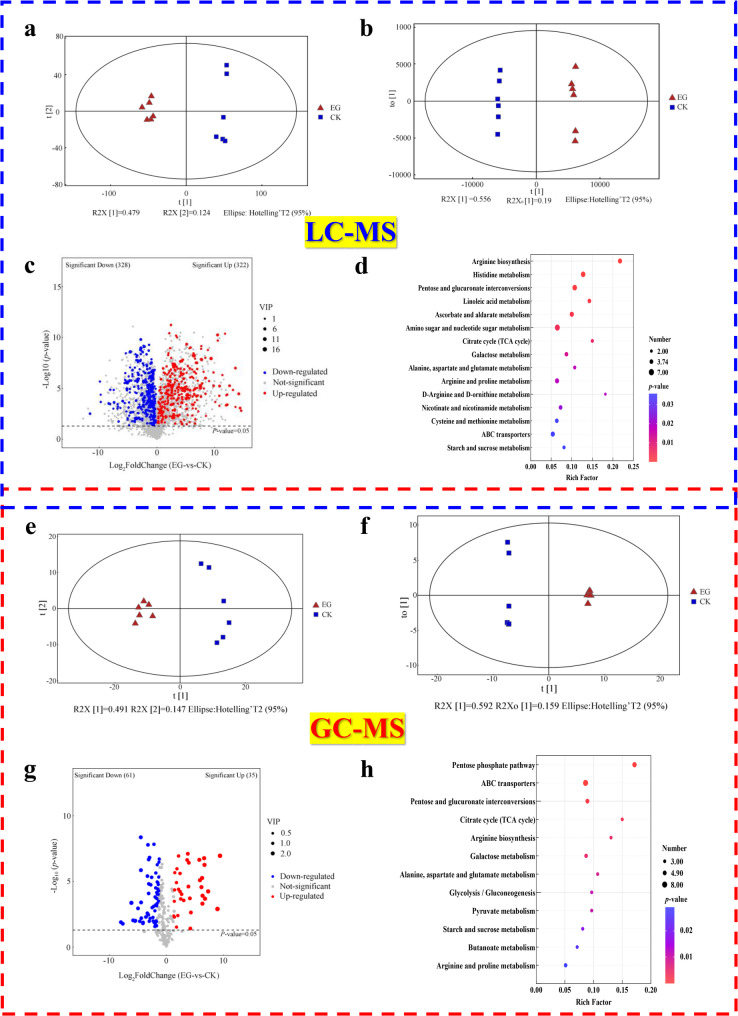
Global metabolomic reprogramming in *S. vaninii* MF5 induced by co-culture with *F. solani* MF20. (**a-d**) LC-MS analysis: **a** PCA score plot; **b** OPLS-DA score plot; **c** Volcano plot of differential metabolites; **d** KEGG pathway enrichment analysis of altered metabolites. (**e-h**) GC-MS analysis: **e** PCA score plot; **f** OPLS-DA score plot; **g** Volcano plot of differential metabolites; **h** KEGG pathway enrichment analysis of altered metabolites. Differential metabolites were defined as VIP > 1 (from OPLS-DA), *p* < 0.05 (Student’s *t*-test), and |FC| > 1. Pathway enrichment was performed using MetaboAnalyst 6.0. CK, axenic control (*n* = 6 biological replicates); EG, co-culture group (*n* = 6 biological replicates)

PCA data from both LC-MS and GC-MS platforms revealed a clear separation between the axenic control (CK) and co-culture (EG) groups (Fig. 10a, e), with quality control samples clustering tightly (Fig. S6a, d; Additional file 1), confirming data reproducibility. This distinct separation demonstrates that the extensive transcriptional reprogramming induced by co-culture successfully translated into a substantial reconstruction of the host’s metabolic landscape, serving as the functional endpoint of the elicited response. Orthogonal partial least squares-discriminant analysis (OPLS-DA) yielded robust models (LC-MS: R2X = 0.802, R2Y = 0.999, Q2 = 0.989; GC-MS: R2X = 0.801, R2Y = 0.999, Q2 = 0.986) with clear group separation (LC-MS: Fig. 10 c; GC-MS: Fig. 10f, g). This identified 650 (322 up, 328 down) and 96 (35 up, 61 down) significantly altered metabolites (VIP > 1, *p* < 0.05) in the LC-MS and GC-MS datasets, respectively (Tables S12, S13; Additional file 3). The overall distribution and major classes of these metabolites are shown in Fig. S6b, c, e, f (Additional file 1).

Integrated KEGG pathway enrichment analysis of the altered metabolites from both platforms revealed a coordinated rewiring of central metabolism (Fig. 10d, h) (Table S14; Additional file 3):

(i) *Enhanced Provision of Reducing Power and Precursors* Significant enrichment of the pentose phosphate pathway (PPP) and related pathways (e.g., pentose and glucuronate interconversions) was a consistent finding. This points to an increased flux towards generating NADPH, which is crucial for both combating oxidative stress and fueling the reductive biosynthesis of secondary metabolites such as flavonoids and terpenoids [142].
(ii) *Reprogramming of Nitrogen Metabolism* Pathways such as arginine biosynthesis and histidine metabolism were significantly altered (identified primarily via LC-MS). This shift suggests a reallocation of nitrogen resources towards specialized compounds; for example, arginine serves as a precursor for polyamines involved in stress tolerance [143].
(iii) *Activation of Lipid-Derived Signaling* Enrichment of linoleic acid metabolism indicates the potential involvement of oxylipins, lipid-based signaling molecules known to mediate fungal defense responses [144].
(iv) *Energy Metabolism and Transport Adjustments* Enrichment of the citrate cycle (TCA cycle) and ABC transporters reflects an adaptation to meet heightened energy demands and to facilitate the export of newly synthesized compounds, respectively,

ensuring the efficient operation of the induced metabolic state [145].

#### Key induced metabolites and their putative functions

We identified the top 30 most significantly up-regulated metabolites from the integrated datasets (Log_2_FC range: 33.10-37.29). Their diverse structures and putative functions underscore a broad activation of host chemical defenses (Table S15; Additional file 2):

(i) *Flavonoids and Phenolic Compounds with Enhanced Modifications* Several glycosylated or acylated flavonoids were strongly induced, including myricetin 3-sambubioside, Luteolin 3’-Methyl Ether 7-Glucuronosyl-(1->2)-Glucuronide, and isoorientin 2’’-*O*-gallate. Such modifications often enhance solubility and stability, facilitating storage and deployment as chemical defenses [146, 147, 148]. The dramatic increase of 6alpha-hydroxyphaseollin, a classic phytoalexin with antimicrobial activity, directly indicates activation of pathogen defense pathways [149].
(ii) *Lipids and Signaling Molecules* Up-regulation of phospholipids like PA(20:1(11Z)/18:1(12Z)-2OH(9,10)) and PPA(16:0/18:1(9Z)) suggests active remodeling of membrane lipids and potential alterations in lipid-based signaling [150, 151]. The increase in 20-F4t-Neurop, a fatty acid derivative, may also be linked to membrane function or signaling [152].
(iii) *Metabolites with Known Pharmacological Analogues* Notably, several induced metabolites are structural analogues of human drugs, including capmatinib (a MET kinase inhibitor [153]), firocoxib (a COX-2 inhibitor [154]), and oxacillin (a β-lactam antibiotic [155]). While their ecological role in fungi is unclear, their induction hints at the profound chemical diversification triggered by the interaction. The significant up-regulation of pachymic acid, a triterpenoid with documented anti-inflammatory and anti-tumor activities, highlights the potential enhancement of the host’s medicinal properties [156].
(iv) *Other Specialized Metabolites* The list also includes compounds like protocatechuic acid 3-*O*-sulfate (an antioxidant derivative [157]), N-(2-hydroxyethyl)ethylenediaminetriacetic acid (a chelator that may aid in metal ion homeostasis under stress [158]), and distemonanthin (reported to possess antibacterial and antioxidant properties [159]), further illustrating the complexity of the metabolic response.
(v) * Induction of Metabolites Structurally Analogous to Known Bioactive Compounds*: The co-culture also triggered the accumulation of several metabolites (Table S15; Additional file 2) that are structural analogues of molecules with established bioactivities in other systems. These include capmatinib (a MET kinase inhibitor [153]), firocoxib (a COX-2 inhibitor [154]), oxacillin (a β-lactam antibiotic [155]), as well as compounds resembling neuroactive or pharmacologically active agents such as befloxatone [160], ipsapirone [161], levonantradol [162], and the anthelmintic agent luxabendazole [163]. It is crucial to emphasize that the biological roles of these specific analogues in the fungal system remain entirely speculative and are likely distinct from their human pharmacological contexts. However, their concerted and significant upregulation serves as a strong chemical signature indicating that the symbiotic interaction has profoundly tapped into the host’s biosynthetic repertoire, pushing its secondary metabolism towards the production of a wide array of structurally complex and nitrogen/sulfur-rich heterocyclic scaffolds.

Hence, this underscores the metabolic plasticity and chemical diversification potential of *S. vaninii* in response to ecological stimuli.

### Integrated transcriptomic and metabolomic analysis reveals *F. solani*-induced reprogramming of bioactive metabolism in *S. vaninii*

To elucidate the molecular mechanisms underlying the biosynthesis of bioactive metabolites in *S. vaninii* MF5 in response to *F. solani* MF20, we performed an integrated analysis of transcriptomic and metabolomic data. This approach directly links upstream transcriptional regulation to downstream metabolite accumulation, establishing a causal evidence chain from gene expression to functional metabolic outcomes.

#### Comprehensive rewiring of Terpenoid biosynthesis

**Fig. 11:**
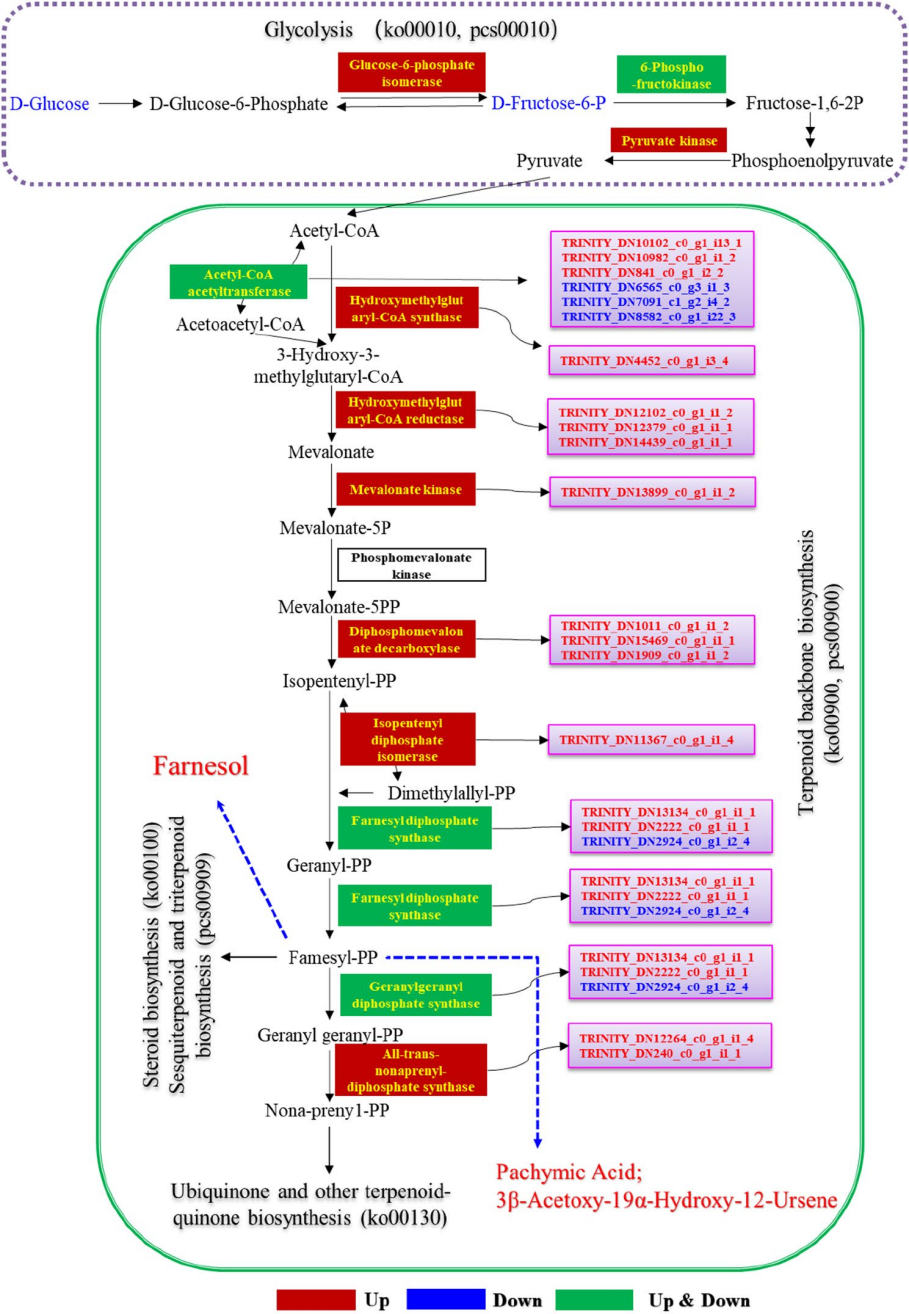
Schematic of the MVA pathway for terpenoid backbone biosynthesis in *S. vaninii* MF5 induced by *F. solani* MF20. Key enzyme genes (arrows) and significantly altered terpenoid metabolites (bars) are shown. Red and blue denote up- and down-regulation, respectively. Schematic of the MVA pathway for terpenoid backbone biosynthesis in *S. vaninii* MF5 based on KEGG annotation. Key enzyme genes and significantly altered terpenoid metabolites are shown. Red and blue denote up- and down-regulation, respectively (|log_2_FC| ≥ 1, adjusted *p* < 0.05 for genes; VIP > 1, *p* < 0.05, |FC| > 1 for metabolites). Transcriptomic data were obtained from RNA-seq (*n* = 6 per group). Metabolite data were obtained from LC-MS and GC-MS analyses (*n* = 6 per group)

Although plants typically employ both the mevalonate (MVA) and methylerythritol phosphate (MEP) pathways for terpenoid synthesis [164], no MEP pathway-related genes were annotated in *S. vaninii*. KEGG enrichment analysis revealed significant alterations in terpenoid backbone biosynthesis (ko00900), steroid biosynthesis (ko00100), and ubiquinone and other terpenoid-quinone biosynthesis (ko00130). Key genes in the MVA pathway were up-regulated, including acetyl-CoA acetyltransferase (*AACT*), hydroxymethylglutaryl-CoA synthase (*HMGS*), hydroxymethylglutaryl-CoA reductase (*HMGR*), mevalonate kinase (*MVK*), diphosphomevalonate decarboxylase (*MVD*), isopentenyl diphosphate isomerase (*IDI*), farnesyl diphosphate synthase (*FPS*), geranylgeranyl diphosphate synthase (*GGPS*), and all-trans-nonaprenyl-diphosphate synthase (*NPPS*) (Fig. 11) (Table S16; Additional file 2).

Metabolomic profiling identified 30 terpenoid compounds, of which 25 were significantly up-regulated and 5 were down-regulated. These included 19 sesquiterpenes, 3 monoterpenes, 5 diterpenes, and 3 triterpenes (Table S17; Additional file 2) (Fig. 11). This broad-spectrum upregulation indicates a global enhancement of the host’s terpenoid biosynthetic capacity, shifting its metabolic state toward a high-yield mode for secondary metabolism. A pivotal signal was the explosive up-regulation (688.65-fold) of farnesol, a sesquiterpene alcohol derived from farnesyl diphosphate. Farnesol acts not only as a precursor for longer-chain terpenes but also as a crucial quorum-sensing and defensive hormone in fungi [165], marking the onset of a heightened defense state in *S. vaninii*. The rapid chemical defense was evidenced by the significant up-regulation of multiple bioactive sesquiterpenes and diterpenes with known antimicrobial properties, such as curdione (20.01-fold) [166], trichoderonin (8.58-fold) [167], and calamendiol (52.36-fold) [168]. The induction of trichoderonin, an antifungal antibiotic analog from *Trichoderma*, suggests *S. vaninii* may employ similar chemical strategies to balance the symbiotic relationship [169]. Furthermore, various oxidized sesquiterpene derivatives (e.g., 5-hydroxyprocurcumenol, 38.91-fold; dehydronootkatone, 186.41-fold) were produced, with oxidation often enhancing bioactivity and solubility, creating a diversified chemical arsenal [170, 171]. The most striking changes were the extreme up-regulation of two triterpenes: pachymic acid (3.16 × 10^10^-fold) and 3β-acetoxy-19α-hydroxy-12-ursene (8.57 × 10^7^-fold). This suggests a shift from immediate defense to a high-cost strategic investment, possibly to stabilize the mutualistic relationship by providing valuable resources or signals to the endophyte. Concurrently, the down-regulation of specific diterpenes like 15-hydroxydehydroabietic acid and ecabet may reflect metabolic flux redistribution favoring induced branches.

Correlation analysis between terpenoid backbone synthesis genes and metabolites revealed strong associations (Fig. S8a; Additional file 1). Most up-regulated genes showed positive correlations with induced terpenoids. A broader network analysis identified the top 100 gene-metabolite relationship pairs (|correlation| > 0.99; Table S18; Additional file 3). Up-regulated triterpenes like pachymic acid showed positive correlations with genes involved in nitrogen metabolism (glutamate synthase), protein folding (peptidylprolyl isomerase), antioxidant synthesis (glutathione synthase), and signaling, while correlating negatively with down-regulated genes such as an AgaK1 protein kinase homolog (Fig. S8b; Additional file 1). These correlations solidify the causal link from transcriptional upregulation to terpenoid accumulation. These strong correlations mechanistically confirm that the transcriptional reprogramming triggered by symbiotic induction is the direct driver of the widespread accumulation of terpenoid metabolites. This finding aligns with recent studies on the metabolic plasticity of *Sanghuangporus* fungi. For instance, Guo et al. (2025) [73] reported that unsaturated fatty acids could specifically induce terpenoid synthesis in *S. lonicericola* via signaling pathways such as MAPK. The advancement of our study lies in revealing that a complex biotic interaction factor (symbiotic fungus) can trigger a more global response: it not only activates the complete synthetic chain from the MVA pathway to multiple classes of terpenes but also initiates a cascade from farnesol signal amplification to the strategic investment in defensive triterpenes. This provides a new perspective for understanding how inter-fungal interactions drive the master switch of secondary metabolism.

#### Systemic modulation of flavonoid biosynthesis

Metabolomic analysis identified 48 significantly altered flavonoid compounds (5 precursors, 43 flavonoids), with 18 up-regulated and 30 down-regulated (Table S19; Additional file 2), indicating a systemic impact on the phenylpropanoid-flavonoid network. The most prominent feature was the extreme accumulation of several modified flavonoids, many among the top 30 induced metabolites. These included isoorientin 2”-*O*-gallate (~ 1.07 × 10^11^-fold), 6alpha-hydroxyphaseollin (~ 7.26 × 10^10^-fold), myricetin 3-sambubioside (~ 6.82 × 10^10^-fold), and distemonanthin (~ 1.15 × 10^10^-fold). This highlights the induction of complex modifications like glycosylation, acylation, and methylation, which alter solubility, stability, and bioactivity [172, 173]. The up-regulation of 6alpha-hydroxyphaseollin, a classic antimicrobial phytoalexin [149], directly indicates pathogen defense pathway activation. The presence of compounds like petunidin 3-(6’’-acetylglucoside) (203.79-fold) suggests activation of pigment or stress-response pathways [174].

**Fig. 12:**
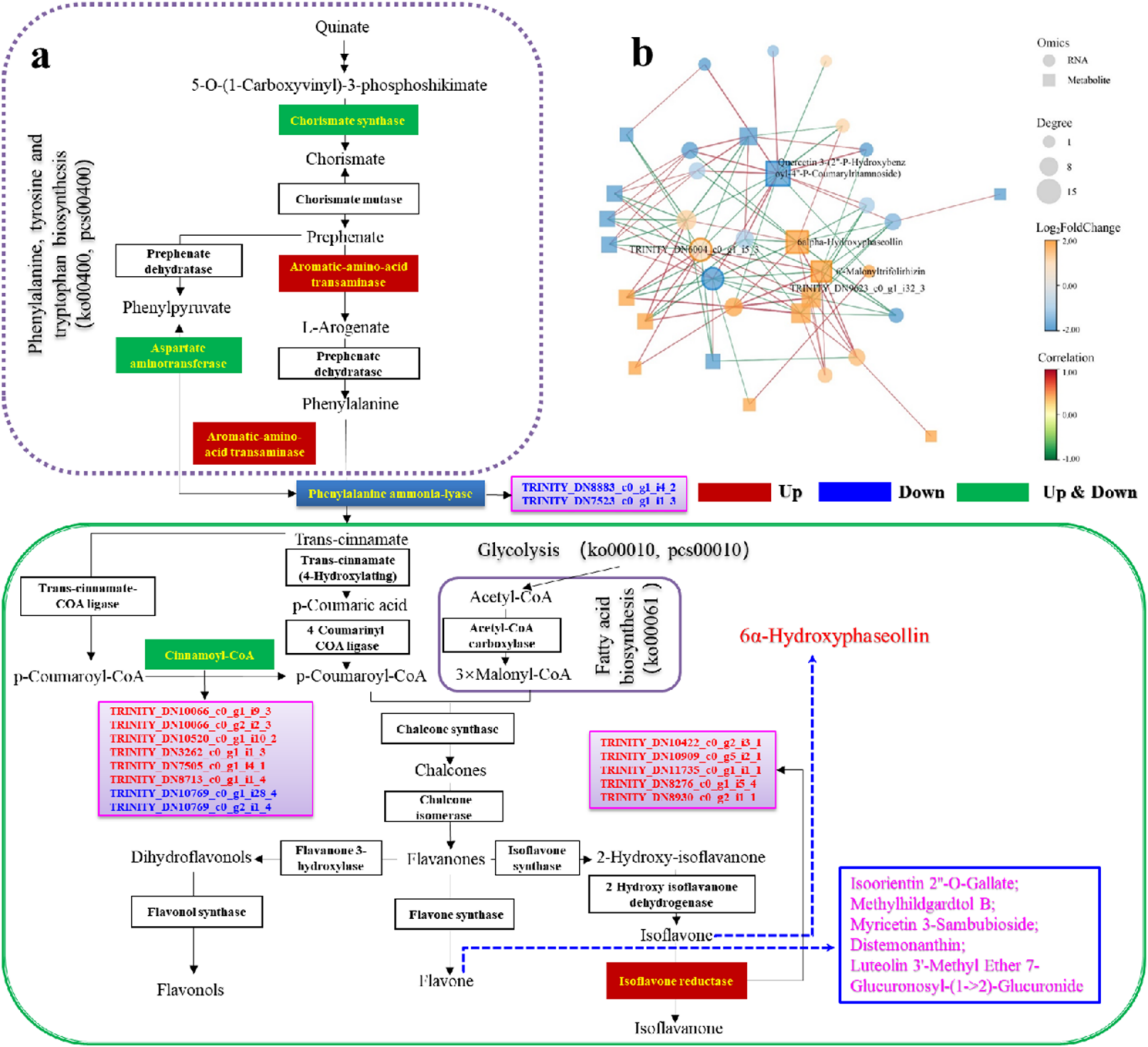
*F. solani* MF20 modulates flavonoid metabolism in *S. vaninii* MF5. **a** Putative flavonoid biosynthesis pathway in *Sanghuangporus* showing key genes and significantly altered flavonoid metabolites. Red and blue denote up- and down-regulation, respectively (|log_2_FC| ≥ 1, adjusted *p* < 0.05 for genes; VIP > 1, *p* < 0.05, |FC| > 1 for metabolites). Transcriptomic and metabolomic data were obtained from six biological replicates per group. **b** Correlation network analysis between significantly altered flavonoids and all DEGs. Node color and size represent log_2_FC and connectivity, respectively. Edge color indicates Spearman correlation coefficient (red: positive; green: negative)

Notably, several typical plant flavonoid biosynthesis genes (e.g., chalcone synthase) were not detected. However, we identified key genes including phenylalanine ammonia-lyase (*PAL*), isoflavone reductase (*IFR*), and cinnamoyl-CoA reductase (*CCR*) as participants in flavonoid synthesis in *S. vaninii* (Fig. 12) (Table S20; Additional file 2), consistent with findings in related fungi [117]. Correlation analysis showed that *CCR* and *IFR* genes positively correlated with up-regulated flavonoids, while down-regulated *PAL* genes correlated negatively with them (Fig. S9; Additional file 1). A comprehensive correlation analysis between all DEGs and flavonoid metabolites revealed that up-regulated flavonoids positively correlated with genes involved in metal ion binding and transcriptional regulation, and negatively correlated with genes encoding fungal hydrophobins and metal transporters (Fig. S10a; Additional file 1) (Table S21; Additional file 3). The above correlation network indicates that flavonoid accumulation is associated with the coordinated activation of a gene module involving broad processes such as metal binding and transcriptional regulation. The extreme chemical diversity produced under symbiotic pressure is remarkable. It is worth noting that *S. vaninii* in this study lacks typical core plant flavonoid synthesis genes (e.g., *CHS*), relying only on a few key genes such as *PAL* and *IFR*. This is consistent with the simplified yet efficient non-canonical synthesis pathway observed by Liu et al. (2022) [175] and Wang et al. (2022) [176] in *S. baumii*. The unique discovery of our study is that on this seemingly simplified” genetic basis, symbiotic stress has stimulated an extremely powerful post-translational modification capacity in the host, producing flavonoid derivatives with unprecedented structural complexity and abundance. This suggests that in fungal flavonoid synthesis, the mobilization of substrate flux and modification enzyme systems may be a more critical limiting step and regulatory target than pathway completeness.

#### Remodeling of polysaccharide metabolism and central carbon flux

**Fig. 13:**
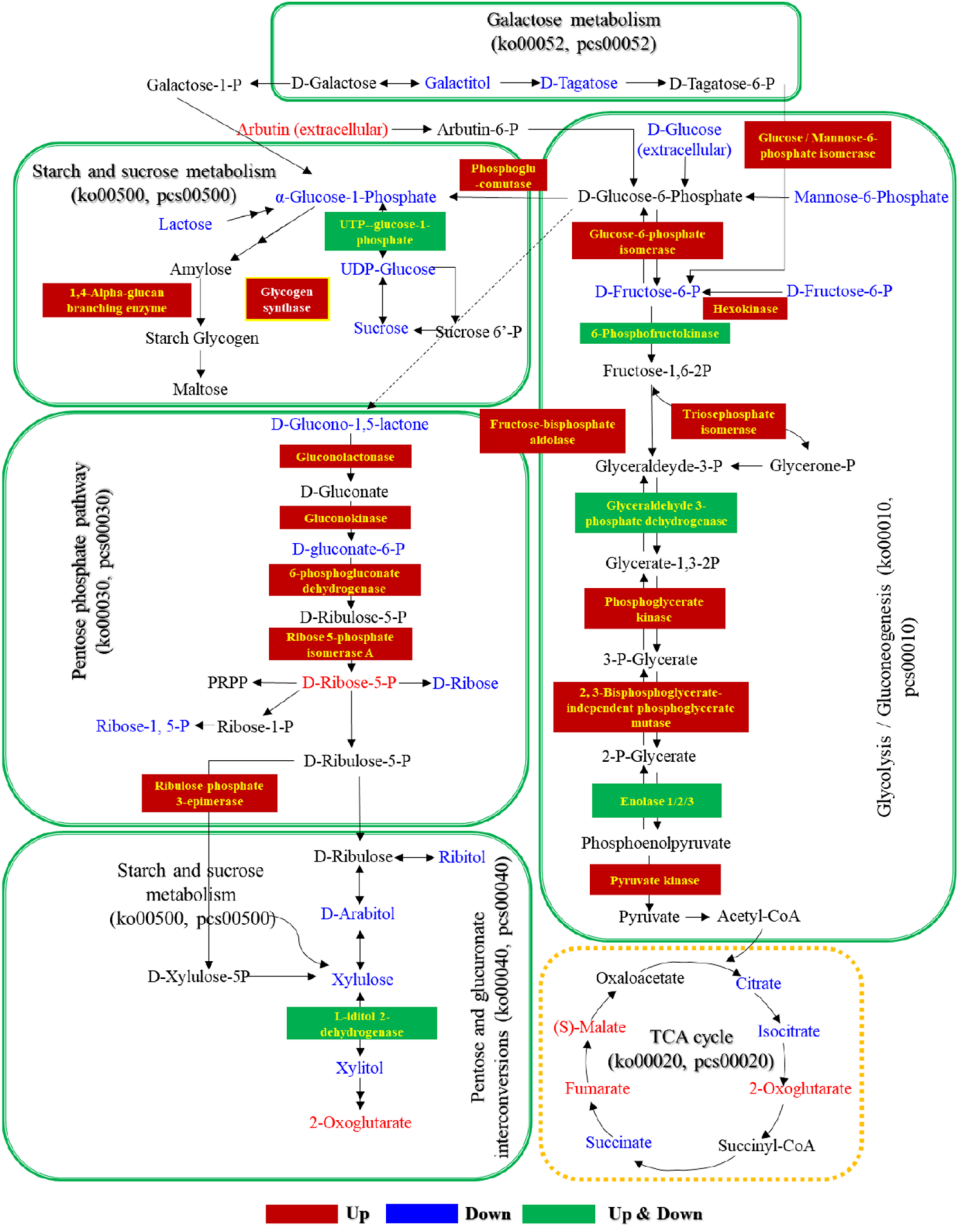
Schematic of polysaccharide and central carbon metabolism pathways of *S. vaninii* MF5 induced by *F. solani* MF20 based on KEGG annotation, highlighting DEGs (red: up-regulated; blue: down-regulated; |log_2_FC| ≥ 1, adjusted *p* < 0.05) and altered sugar-related metabolites (VIP > 1, *p* < 0.05, |FC| > 1). Transcriptomic and metabolomic data were obtained from six biological replicates per group

Integrated analysis revealed significant enrichment of 235 DEGs (Table S22; Additional file 3) and associated metabolites in central carbon metabolism pathways, including galactose metabolism, pentose phosphate pathway (PPP), starch and sucrose metabolism, and the TCA cycle (Fig. 13). Among 96 DEGs related to polysaccharide synthesis, key enzymes like hexokinase, phosphoglucomutase, and UDP-glucose pyrophosphorylase were predominantly up-regulated.

Metabolomic profiling identified 31 sugar-related metabolites, with 26 significantly down-regulated, including key sugars like *D*-glucose and sucrose (Table S23; Additional file 2). This suggests a potential overall inhibition of carbohydrate storage or a redirection of carbon flux. Intermediates of the PPP, glycolysis, and TCA cycle were also mostly down-regulated, indicating a reshaping of central carbon metabolism. In contrast, oxoglutaric acid (α-ketoglutarate) was dramatically up-regulated (> 55-fold). As a key TCA cycle intermediate and a central molecule in nitrogen assimilation [177], its accumulation reflects a reprogramming of energy metabolism and amino acid synthesis under metabolic stress. The up-regulation of arbutin, a phenolic glycoside, may be linked to activated defense or antioxidant pathways [178].

Correlation analysis showed that up-regulated compounds like oxoglutaric acid positively correlated with stress-response genes (e.g., *Hsp40 co-chaperone Jid1*), while down-regulated sugars negatively correlated with genes involved in lyase activity and protein synthesis (Fig. S10b; Additional file 1) (Table S24; Additional file 3). This aligns with the transcriptomic enrichment, confirming that the endophyte impacts precursor supply and carbon skeleton flow for polysaccharide metabolism. These results collectively indicate that the endophytic fungus affects the precursor supply and carbon skeleton flow for polysaccharide synthesis in the host, leading to profound remodeling of central carbon metabolism. This resource reallocation model is essentially consistent with the findings of Li et al. (2023) [179] and Wang et al. (2025) [180], who used multi-omics techniques to elucidate the high-yield polysaccharide mechanism in *S. sanghuang*. The difference and depth of our study lie in revealing a more complex metabolic trade-off: the aforementioned research focused on maximizing the synthesis flux of a single target product (polysaccharide), whereas our study demonstrates the global reconstruction of the host’s carbon metabolic network to support this “systemic chemical innovation” under the background of simultaneous strong induction of multiple secondary metabolites such as terpenoids and flavonoids, possibly at the cost of consuming primary storage substances (e.g., free sugars).

#### Other significant correlations validation and broader correlation network

The expression patterns of selected key genes related to the discussed bioactive compounds were validated by qRT-PCR, confirming consistency with the transcriptomic data (Fig. S7; Additional file 1).

**Fig. 14:**
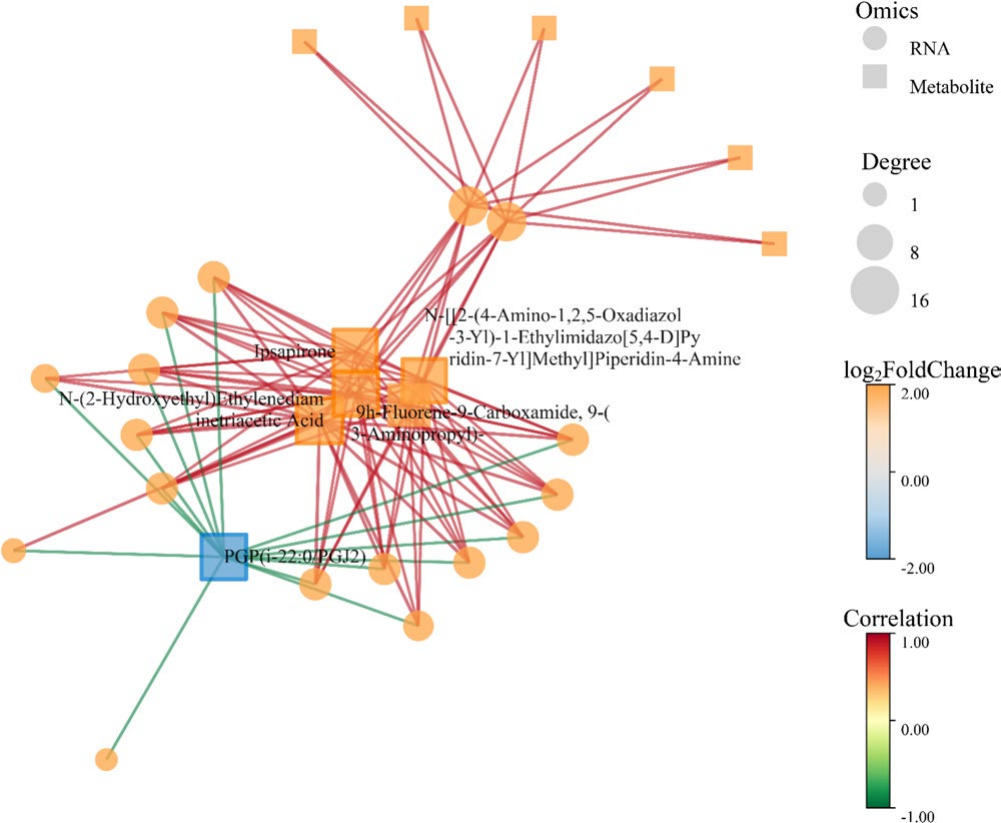
Correlation network analysis of other highly induced metabolites in *S. vaninii* MF5 under co-culture conditions. Network showing strong correlations (|Spearman’s ρ| > 0.99, *p* < 0.001) between other significantly up-regulated metabolites (VIP > 1, *p* < 0.05, |FC| > 1; e.g., structural analogues of capmatinib, firocoxib) and a suite of up-regulated genes (|log_2_FC| ≥ 1, adjusted *p* < 0.05). Node shape indicates data type (square: metabolite; circle: gene). Node shape indicates data type (square: metabolite; circle: gene). Node color and size represent fold-change and connectivity, respectively. Edge color indicates correlation coefficient (red: positive; green: negative). Top 5 hub nodes are labeled. Data were integrated from transcriptomic (RNA-seq, *n* = 6 per group) and metabolomic (LC-MS and GC-MS, *n* = 6 per group) analyses

Beyond the specific pathways, a broad correlation network analysis highlighted significant positive correlations between many other highly up-regulated metabolites (Log_2_FC > 32.77, including several top-30 metabolites like capmatinib and firocoxib) and a suite of up-regulated genes encoding proteins with diverse functions (e.g., transmembrane proteins, transcriptional regulators, acetyltransferases) (Fig. 14) (Table S25; Additional file 3). This pattern underscores that the endophytic stimulus triggers a systemic metabolic reprogramming. The host appears to activate a comprehensive network to redirect resources from primary growth metabolism toward synthesizing a wide array of complex, specialized secondary metabolites, representing a holistic defense and adaptation strategy.

## Conclusion

This study successfully established a novel biotic elicitation strategy by co-cultivating the medicinal *S. vaninii* with its native endophyte *F. solani* MF20. This approach dramatically enhanced the production of pharmaceutically relevant bioactive metabolites (flavonoids, terpenoids, and polysaccharides) with yields increased by up to 9.38-fold. Integrated transcriptomic and metabolomic analyses were employed to decipher the underlying regulatory mechanisms, and the results revealed that the endophytic interaction induced a biotic elicitation - mediated controlled stress in the host, initiating specific early signaling events including a NOX-mediated reactive oxygen species (ROS) burst, extracellular ATP (eATP) accumulation, and Ca^2+^ influx. This signaling cascade further was associated with a global metabolic reprogramming in *S. vaninii*, which involved the redirection of cellular resources from primary growth toward chemical defense. Key biosynthetic pathways, particularly those for terpenoid and flavonoid backbones, were transcriptionally up-regulated, which was directly corroborated by the massive accumulation of diverse and modified bioactive compounds (e.g., pachymic acid and complex flavonoid glycosides). Concurrently, central carbon metabolism was reshaped, and the activated pentose phosphate pathway potentially supplied essential reducing power (NADPH) for biosynthesis. These findings provide the first comprehensive molecular evidence for *Sanghuangporus* that a native endophyte can act as a powerful ecological trigger to unlock the metabolic potential of its host fungus. Beyond advancing our understanding of fungal-fungal symbiotic communication, this work validates endophyte co-culture as a sustainable and green bio-process technology, which offers a viable and efficient alternative to conventional chemical elicitation or genetic modification for the enhanced and sustainable bioproduction of high-value compounds from medicinal fungal resources, with significant implications for industrial biotechnology and green bioresource utilization of edible-medicinal fungi.

A comprehensive metabolomic analysis of *F. solani* MF20, including its volatile and non‑volatile metabolite profiles and their regulatory effects on *S. vaninii*, is currently underway and will be reported separately. This ongoing work will provide deeper insights into the molecular dialogue between these two fungi and further elucidate the elicitor‑active compounds secreted by the endophyte.

## Supplementary Information

Below is the link to the electronic supplementary material.

Supplementary Material 1. (See in Supplementary *PPT*). Fig. S1. Time-course dynamics of mycelial growth and metabolite accumulation in *S. vaninii* MF5 during solid-state fermentation. (a) Morphological development of *S. vaninii* MF5 colonies on PDA plates over a 16-day period. (b) Accumulation profiles of total flavonoids and terpenoids in *S. vaninii* MF5 mycelia during fermentation. (c) Accumulation profile of crude polysaccharides in *S. vaninii* MF5 mycelia during fermentation. Fig. S2. Isolation, identification of endophytic fungi and their initial effects on the host. (a) Representative images of endophytic fungal strains isolated from surface-sterilized fruiting bodies of *S. vaninii*. (b) Phylogenetic tree of the endophytic strain MF20 based on ITS rDNA sequence analysis, showing its close relationship to *F. solani*. (c) Phenotypic changes and quantitative analysis of major metabolites in *S. vaninii* MF5 during initial plate co-culture with endophytic isolate MF20. Fig. S3. Transcriptomic sequencing analysis of *S. vaninii* MF5 in response to co-culture with *F. solani* MF20. (a) PCA score plot of RNA-seq samples from the axenic control (CK) and co-culture (EG) groups. (b) Volcano plot displaying DEGs in *S. vaninii* MF5 induced by co-culture with *F. solani* MF20. Fig. S4. qRT-PCR validation of RNA-seq data. (a) Validation of gene expression levels for ten randomly selected genes from KEGG and GO enrichment analyses. *CYP450* (Cytochrome P450); *TrpH* (Tryptophan halogenase); *TDH* (Tartrate dehydrogenase); *ArgC* (N-acetyl-gamma-glutamyl-phosphate reductase); *RecQ* (ATP-dependent DNA helicase RecQ); *AACT* (Acetyl-CoA acetyltransferase); *PckA* (Phosphoenolpyruvate carboxykinase (ATP)); *ADH1* (Alcohol dehydrogenase); *PRDX1* (Putative peroxiredoxin). (b) Validation of gene expression levels related to development, calcium signaling, ATP metabolism, and antioxidant enzymes. *GH16* (Glycosyl hydrolases family 16); *S6K* (Ribosomal protein S6 kinase); *STK* (Serine/threonine protein kinase); *HSP20* (Heat shock protein 20); *PMFS* (Major facilitator superfamily permease); *ABC1* (ABC transporter ATP-binding/permease protein); *ACS* (Acyl-CoA synthetase); *GCDH* (Glutaryl-CoA dehydrogenase); *NOX-1/2* (NADPH oxidase); *CAT-POD* (Catalase-Peroxidase); *GPX* (Glutathione peroxidase); *AHR* (Alkyl hydroperoxide reductase); *POD* (Peroxidase); *PMSOR* (Peptide-methionine (R)-S-oxide reductase); *CAT* (Catalase); *Rho type* (Ras-related small GTPase); *ABC transporter-3/4* (ABC transporter); *PKA* (cAMP-dependent protein kinase); *Cdc42 Rac* (P21 protein (Cdc42 Rac)-activated kinase); *Calcium Proton-1/2* (Calcium/proton exchanger); *Ppase* (ATP-dependent serine protease); *MAPKK* (Mitogen-activated protein kinase kinase kinase); *Ca*^*2+*^*-ATPase* (Ca^2+^ transporting ATPase). Fig. S5. GO enrichment analysis of DEGs in *S. vaninii* MF5 induced by co-culture with *F. solani* MF20. Enriched GO terms across Biological Process (BP), Molecular Function (MF), and Cellular Component (CC) categories are shown. Fig. S6. Untargeted metabolomic profiling of *S. vaninii* MF5 in response to co-culture with *F. solani* MF20 using dual-platform (LC-MS and GC-MS) analysis. (a-c) Results from LC-MS analysis: (a) PCA score plot; (b) Volcano plot of differential metabolites (DMs); (c) Classification and distribution of identified metabolites. (d-f) Results from GC-MS analysis: (d) PCA score plot; (e) Volcano plot of differential metabolites (DMs); (f) Total ion chromatogram (TIC) representative overlay. Fig. S7. Validation of gene expression levels associated with key metabolite biosynthetic pathways. qRT-PCR analysis of genes involved in the terpenoid backbone biosynthesis pathway (*AACT*, *HMGS*, *HMGR*, *MVK*, *MVD*, *IDI*, *GGPS*, *NNPS*) and the flavonoid biosynthesis pathway (*PAL*, *CCR*, *IFR*) in *S. vaninii* MF5 under co-culture conditions. *AACT* (Acetyl-CoA C-acetyltransferase), *HMGS* (Hydroxymethylglutaryl-CoA synthase), *HMGR* (Hydroxymethylglutaryl-CoA reductase), *MVK* (Mevalonate kinase), *MVD* (Diphosphomevalonate decarboxylase), *IDI* (Isopentenyl diphosphate isomerase), *GGPS* (Geranylgeranyl diphosphate synthase), *NNPS* (All-trans-nonaprenyl-diphosphate synthase), *PAL* (Phenylalanine ammonia-lyase), *CCR* (Cinnamoyl-CoA reductase), *IFR* (Isoflavone reductase). Fig. S8. (a) Correlation heatmap between expression levels of terpenoid biosynthetic genes and abundance of terpenoid metabolites. (b) Correlation network analysis between the most significantly altered terpenoids and all DEGs. Node shape indicates data type (square: metabolite; circle: gene). Node color and size represent fold-change and connectivity, respectively. Edge color indicates correlation coefficient (red: positive; green: negative). Top 5 hub nodes are labeled. Fig. S9. Correlation heatmap between flavonoid-related gene expression and flavonoid metabolite abundance. Fig. S10. (a) Correlation network analysis between significantly altered flavonoids and all DEGs. Node shape indicates data type (square: metabolite; circle: gene). Node color and size represent fold-change and connectivity, respectively. Edge color indicates correlation coefficient. Top 5 hub nodes are labeled. (b) Correlation network analysis between polysaccharide-related metabolites and all DEGs. Node shape indicates data type (square: metabolite; circle: gene). Node color and size represent fold-change and connectivity, respectively. Edge color indicates correlation coefficient. Top 5 hub nodes are labeled.

Supplementary Material 2. (See in Supplementary *Word*). Table S2. Output statistics and quality assessment of RNA sequencing data for *S. vaninii* MF5 samples from control and co-culture with *F. solani* MF20 groups. Table S3. Summary statistics of unigene annotation for the *S. vaninii* MF5 transcriptome assembly against various public databases. Table S8. List and annotation of DEGs involved in the developmental process and hyphal morphogenesis of *S. vaninii* MF5 induced by *F. solani* MF20. Table S9. List and annotation of DEGs related to cell membrane permeabilization and fatty acid metabolism in *S. vaninii* MF5 induced by *F. solani* MF20. Table S10. List and annotation of DEGs involved in ATP metabolism and calcium signaling/transport in *S. vaninii* MF5 induced by *F. solani* MF20. Table S11. List and annotation of DEGs involved in oxidative stress response and signal transduction in *S. vaninii* MF5 induced by *F. solani* MF20. Table S15. Top 30 most significantly up-regulated differential metabolites (DMs) in *S. vaninii* MF5 induced by co-culture with *F. solani* MF20. Table S16. List and annotation of DEGs involved in the terpenoid backbone biosynthesis pathway of *S. vaninii* MF5 induced by *F. solani* MF20. Table S17. List of differential metabolites (DMs) identified as terpenoids in *S. vaninii* MF5 induced by co-culture with *F. solani* MF20. Table S19. List of differential metabolites (DMs) identified as flavonoids and isoflavonoids in *S. vaninii* MF5 induced by co-culture with *F. solani* MF20. Table S20. List and annotation of DEGs potentially involved in flavonoid and isoflavonoid biosynthesis in *S. vaninii* MF5 induced by *F. solani* MF20. Table S23. List of differential metabolites (DMs) related to polysaccharide and central carbon metabolism in *S. vaninii* MF5 induced by co-culture with F. solani MF20

Supplementary Material 3. (See in Supplementary *Excel* file). Table S1. Primer sequences used for qRT-PCR validation. Table S4. Annotation of DEGs using NR, SWISS-Prot, KEGG, KOG, eggNOG, GO and Pfam databases and their fold change of *S. vaninii* MF5 by *F. solani* MF20. Table S5. GO enrichment of *S. vaninii* MF5 by *F. solani* MF20. Table S6. KEGG enrichment of *S. vaninii* MF5 by *F. solani* MF20. Table S7. Annotation of genes exclusively expressed in the co-culture group (“Inf”) or the control group (“0”). Table S12. Differential metabolites (DMs) detected by LC-MS of *S. vaninii* MF5 by *F. sollani* MF20. Table S13. Differential metabolites (DMs) detected by GC-MS of *S. vaninii* MF5 by *F. sollani* MF20. Table S14. Results of KEGG pathway enrichment analysis for differential metabolites (DMs) from both LC-MS and GC-MS platforms. Table S18. Correlation analysis results between DEGs and differential metabolites (DMs) involved in terpenoid metabolism. Table S21. Correlation analysis results between DEGs and differential metabolites (DMs) involved in flavonoid metabolism. Table S22. List and annotation of DEGs involved in polysaccharide metabolism of *S. vaninii* MF5. Table S24. Correlation analysis results between DEGs and differential metabolites (DMs) involved in polysaccharide and central carbon metabolism. Table S25. List of the top gene-metabolite pairs showing the strongest correlation coefficients in the integrated network analysis.

## Data Availability

The datasets generated and analyzed during the current study are available in the following public repositories: Transcriptomic data have been deposited in the NCBI Gene Expression Omnibus (GEO) under accession number GSE272365.Metabolomic data have been deposited in the Genome Sequence Archive (GSA) under accession number OMIX007469.The ITS rDNA sequence of Fusarium solani MF20 is available in GenBank under accession number OR672754.The strains Sanghuangporus vaninii MF5 and Fusarium solani MF20 are deposited at the China Center for Type Culture Collection (CCTCC) under accession numbers CCTCC AF 2023066 and CCTCC M 20232175, respectively.All data generated or analyzed during this study are included in this published article and its supplementary information files.
