# Supplementary material for: Fungal-fungal interaction between Sanghuangporus vaninii and its endophytic Fusarium solani rewires host secondary metabolism to boost bioactive metabolite production
Source: Microb Cell Fact. 2026 Apr 4;25:135. doi: 10.1186/s12934-026-02994-z (PMC13214342; doi:10.1186/s12934-026-02994-z)
Supplement: Supplementary file 1 — Supplementary Material 1. (See in Supplementary PPT). Fig. S1. Time-course dynamics of mycelial growth and metabolite accumulation in S. vaninii MF5 during solid-state fermentation. (a) Morphological development of S. vaninii MF5 colonies on PDA plates over a 16-day period. (b) Accumulation profiles of total flavonoids and terpenoids in S. vaninii MF5 mycelia during fermentation. (c) Accumulation profile of crude polysaccharides in S. vaninii MF5 mycelia during fermentation. Fig. S2. Isolation, identification of endophytic fungi and their initial effects on the host. (a) Representative images of endophytic fungal strains isolated from surface-sterilized fruiting bodies of S. vaninii. (b) Phylogenetic tree of the endophytic strain MF20 based on ITS rDNA sequence analysis, showing its close relationship to F. solani. (c) Phenotypic changes and quantitative analysis of major metabolites in S. vaninii MF5 during initial plate co-culture with endophytic isolate MF20. Fig. S3. Transcriptomic sequencing analysis of S. vaninii MF5 in response to co-culture with F. solani MF20. (a) PCA score plot of RNA-seq samples from the axenic control (CK) and co-culture (EG) groups. (b) Volcano plot displaying DEGs in S. vaninii MF5 induced by co-culture with F. solani MF20. Fig. S4. qRT-PCR validation of RNA-seq data. (a) Validation of gene expression levels for ten randomly selected genes from KEGG and GO enrichment analyses. CYP450 (Cytochrome P450); TrpH (Tryptophan halogenase); TDH (Tartrate dehydrogenase); ArgC (N-acetyl-gamma-glutamyl-phosphate reductase); RecQ (ATP-dependent DNA helicase RecQ); AACT (Acetyl-CoA acetyltransferase); PckA (Phosphoenolpyruvate carboxykinase (ATP)); ADH1 (Alcohol dehydrogenase); PRDX1 (Putative peroxiredoxin). (b) Validation of gene expression levels related to development, calcium signaling, ATP metabolism, and antioxidant enzymes. GH16 (Glycosyl hydrolases family 16); S6K (Ribosomal protein S6 kinase); STK (Serine/threonine protein kinase); HSP20 ( [file 12934_2026_2994_MOESM1_ESM.pptx]

## Slide 1
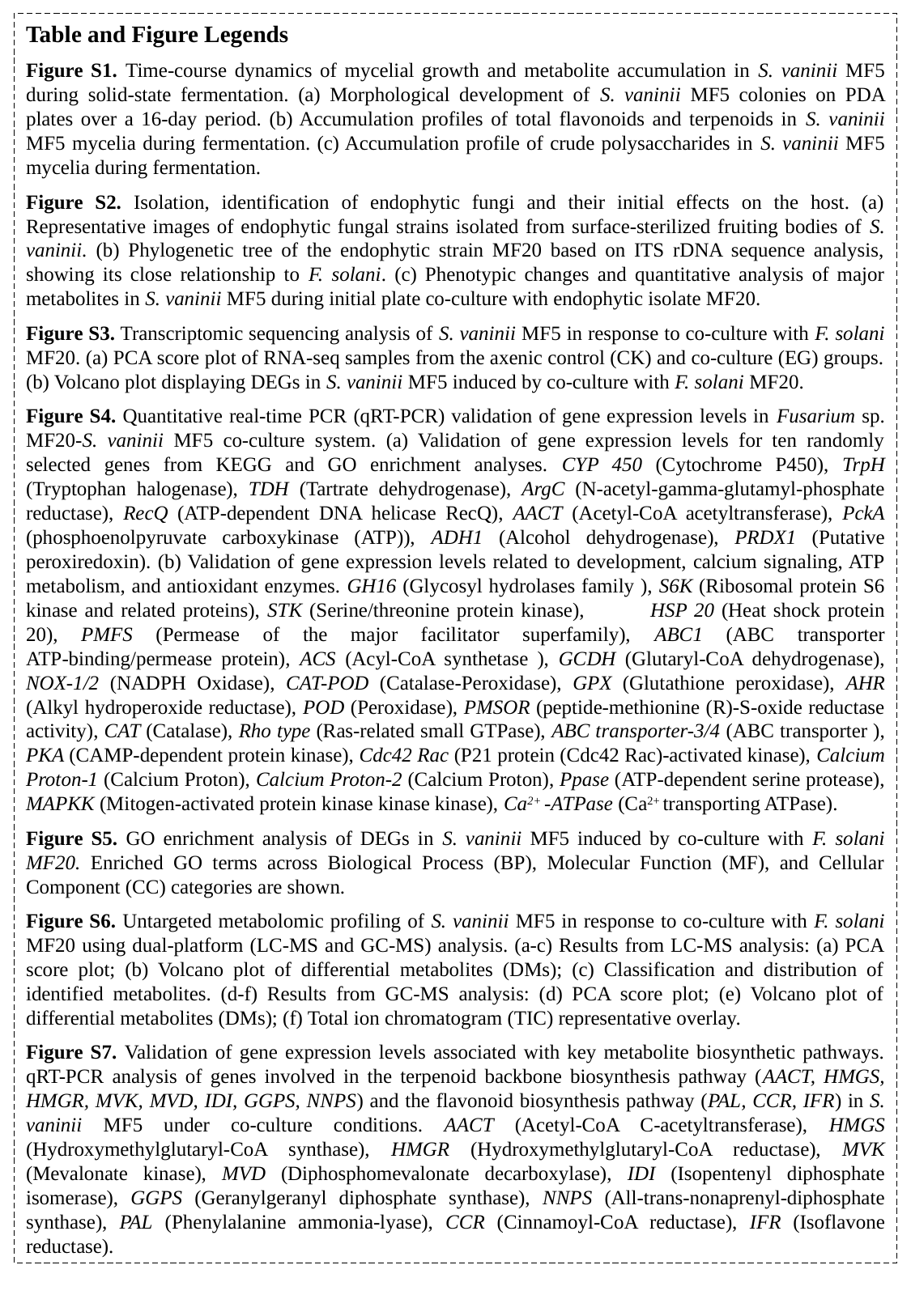

Table and Figure Legends
Figure S1. Time-course dynamics of mycelial growth and metabolite accumulation in S. vaninii MF5 during solid-state fermentation. (a) Morphological development of S. vaninii MF5 colonies on PDA plates over a 16-day period. (b) Accumulation profiles of total flavonoids and terpenoids in S. vaninii MF5 mycelia during fermentation. (c) Accumulation profile of crude polysaccharides in S. vaninii MF5 mycelia during fermentation.
Figure S2. Isolation, identification of endophytic fungi and their initial effects on the host. (a) Representative images of endophytic fungal strains isolated from surface-sterilized fruiting bodies of S. vaninii. (b) Phylogenetic tree of the endophytic strain MF20 based on ITS rDNA sequence analysis, showing its close relationship to F. solani. (c) Phenotypic changes and quantitative analysis of major metabolites in S. vaninii MF5 during initial plate co-culture with endophytic isolate MF20.
Figure S3. Transcriptomic sequencing analysis of S. vaninii MF5 in response to co-culture with F. solani MF20. (a) PCA score plot of RNA-seq samples from the axenic control (CK) and co-culture (EG) groups. (b) Volcano plot displaying DEGs in S. vaninii MF5 induced by co-culture with F. solani MF20.
Figure S4. Quantitative real-time PCR (qRT-PCR) validation of gene expression levels in Fusarium sp. MF20-S. vaninii MF5 co-culture system. (a) Validation of gene expression levels for ten randomly selected genes from KEGG and GO enrichment analyses. CYP 450 (Cytochrome P450), TrpH (Tryptophan halogenase), TDH (Tartrate dehydrogenase), ArgC (N-acetyl-gamma-glutamyl-phosphate reductase), RecQ (ATP-dependent DNA helicase RecQ), AACT (Acetyl-CoA acetyltransferase), PckA (phosphoenolpyruvate carboxykinase (ATP)), ADH1 (Alcohol dehydrogenase), PRDX1 (Putative peroxiredoxin). (b) Validation of gene expression levels related to development, calcium signaling, ATP metabolism, and antioxidant enzymes. GH16 (Glycosyl hydrolases family ), S6K (Ribosomal protein S6 kinase and related proteins), STK (Serine/threonine protein kinase), 	HSP 20 (Heat shock protein 20), PMFS (Permease of the major facilitator superfamily), ABC1 (ABC transporter ATP-binding/permease protein), ACS (Acyl-CoA synthetase ), GCDH (Glutaryl-CoA dehydrogenase), NOX-1/2 (NADPH Oxidase), CAT-POD (Catalase-Peroxidase), GPX (Glutathione peroxidase), AHR (Alkyl hydroperoxide reductase), POD (Peroxidase), PMSOR (peptide-methionine (R)-S-oxide reductase activity), CAT (Catalase), Rho type (Ras-related small GTPase), ABC transporter-3/4 (ABC transporter ), PKA (CAMP-dependent protein kinase), Cdc42 Rac (P21 protein (Cdc42 Rac)-activated kinase), Calcium Proton-1 (Calcium Proton), Calcium Proton-2 (Calcium Proton), Ppase (ATP-dependent serine protease), MAPKK (Mitogen-activated protein kinase kinase kinase), Ca2+ -ATPase (Ca2+ transporting ATPase).
Figure S5. GO enrichment analysis of DEGs in S. vaninii MF5 induced by co-culture with F. solani MF20. Enriched GO terms across Biological Process (BP), Molecular Function (MF), and Cellular Component (CC) categories are shown.
Figure S6. Untargeted metabolomic profiling of S. vaninii MF5 in response to co-culture with F. solani MF20 using dual-platform (LC-MS and GC-MS) analysis. (a-c) Results from LC-MS analysis: (a) PCA score plot; (b) Volcano plot of differential metabolites (DMs); (c) Classification and distribution of identified metabolites. (d-f) Results from GC-MS analysis: (d) PCA score plot; (e) Volcano plot of differential metabolites (DMs); (f) Total ion chromatogram (TIC) representative overlay.
Figure S7. Validation of gene expression levels associated with key metabolite biosynthetic pathways. qRT-PCR analysis of genes involved in the terpenoid backbone biosynthesis pathway (AACT, HMGS, HMGR, MVK, MVD, IDI, GGPS, NNPS) and the flavonoid biosynthesis pathway (PAL, CCR, IFR) in S. vaninii MF5 under co-culture conditions. AACT (Acetyl-CoA C-acetyltransferase), HMGS (Hydroxymethylglutaryl-CoA synthase), HMGR (Hydroxymethylglutaryl-CoA reductase), MVK (Mevalonate kinase), MVD (Diphosphomevalonate decarboxylase), IDI (Isopentenyl diphosphate isomerase), GGPS (Geranylgeranyl diphosphate synthase), NNPS (All-trans-nonaprenyl-diphosphate synthase), PAL (Phenylalanine ammonia-lyase), CCR (Cinnamoyl-CoA reductase), IFR (Isoflavone reductase).

## Slide 2
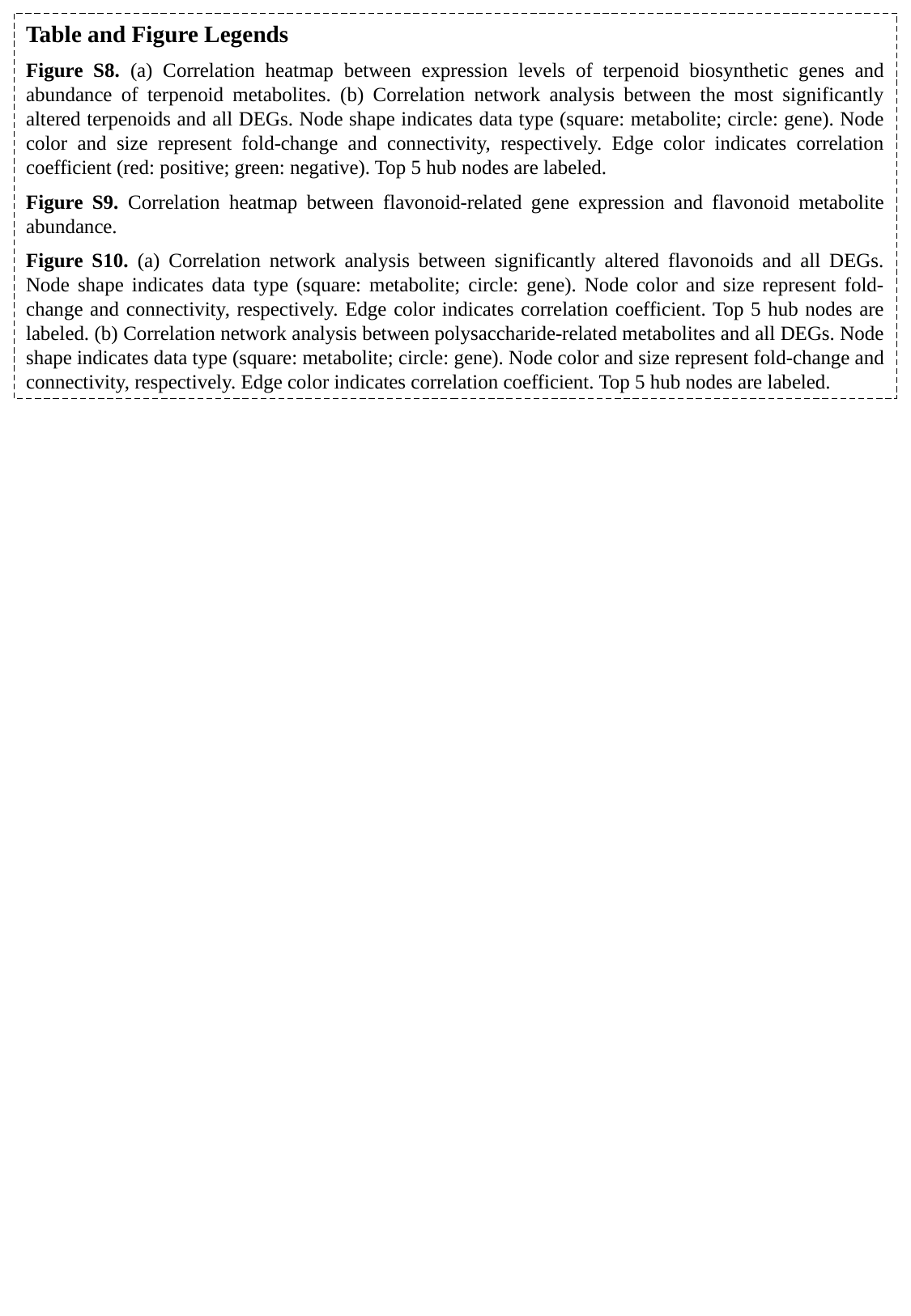

Table and Figure Legends
Figure S8. (a) Correlation heatmap between expression levels of terpenoid biosynthetic genes and abundance of terpenoid metabolites. (b) Correlation network analysis between the most significantly altered terpenoids and all DEGs. Node shape indicates data type (square: metabolite; circle: gene). Node color and size represent fold-change and connectivity, respectively. Edge color indicates correlation coefficient (red: positive; green: negative). Top 5 hub nodes are labeled.
Figure S9. Correlation heatmap between flavonoid-related gene expression and flavonoid metabolite abundance.
Figure S10. (a) Correlation network analysis between significantly altered flavonoids and all DEGs. Node shape indicates data type (square: metabolite; circle: gene). Node color and size represent fold-change and connectivity, respectively. Edge color indicates correlation coefficient. Top 5 hub nodes are labeled. (b) Correlation network analysis between polysaccharide-related metabolites and all DEGs. Node shape indicates data type (square: metabolite; circle: gene). Node color and size represent fold-change and connectivity, respectively. Edge color indicates correlation coefficient. Top 5 hub nodes are labeled.

## Slide 3
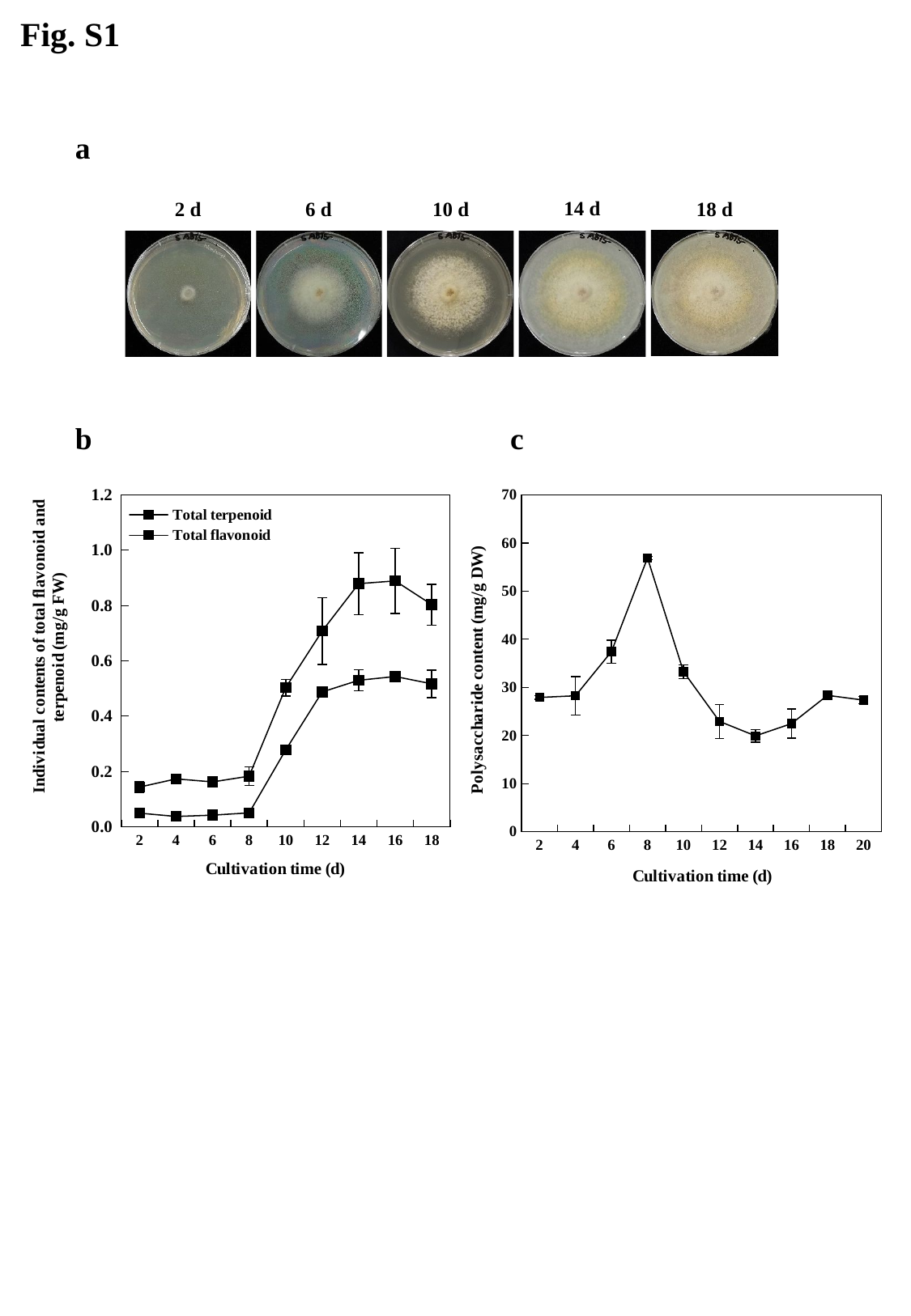

Fig. S1
a
14 d
2 d
6 d
10 d
18 d
b
c
### Chart
| Category | |
|---|---|
| 2 | 27.8957272793071 |
| 4 | 28.2475652810281 |
| 6 | 37.4456158974483 |
| 8 | 56.8972311354516 |
| 10 | 33.2738224484708 |
| 12 | 22.9197326835388 |
| 14 | 19.9039783830732 |
| 16 | 22.4522907669666 |
| 18 | 28.3279853957072 |
| 20 | 27.3428389908884 |
### Chart
| Category | Total terpenoid | Total flavonoid |
|---|---|---|
| 2 | 0.14395600940185 | 0.0495812347510951 |
| 4 | 0.173435055695745 | 0.0376912503983145 |
| 6 | 0.16262607205465 | 0.0424472441394267 |
| 8 | 0.183261404460376 | 0.0507702331863731 |
| 10 | 0.503109056749129 | 0.278106734011538 |
| 12 | 0.707890165004048 | 0.48772715815106 |
| 14 | 0.879163423971574 | 0.529936602603431 |
| 16 | 0.888989772736206 | 0.543491184765601 |
| 18 | 0.80330401150862 | 0.517214319345956 |

## Slide 4
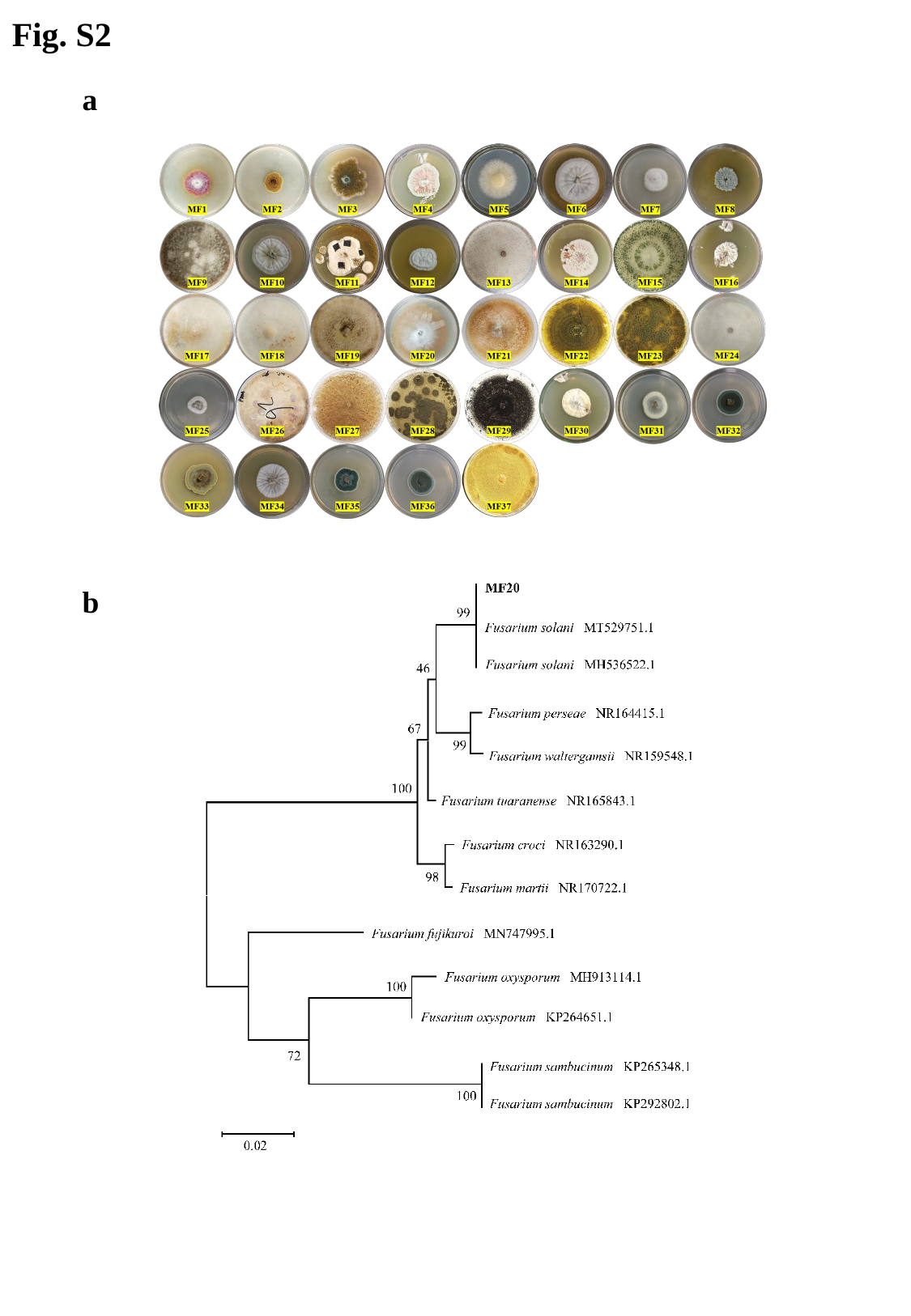

Fig. S2
a
b

## Slide 5
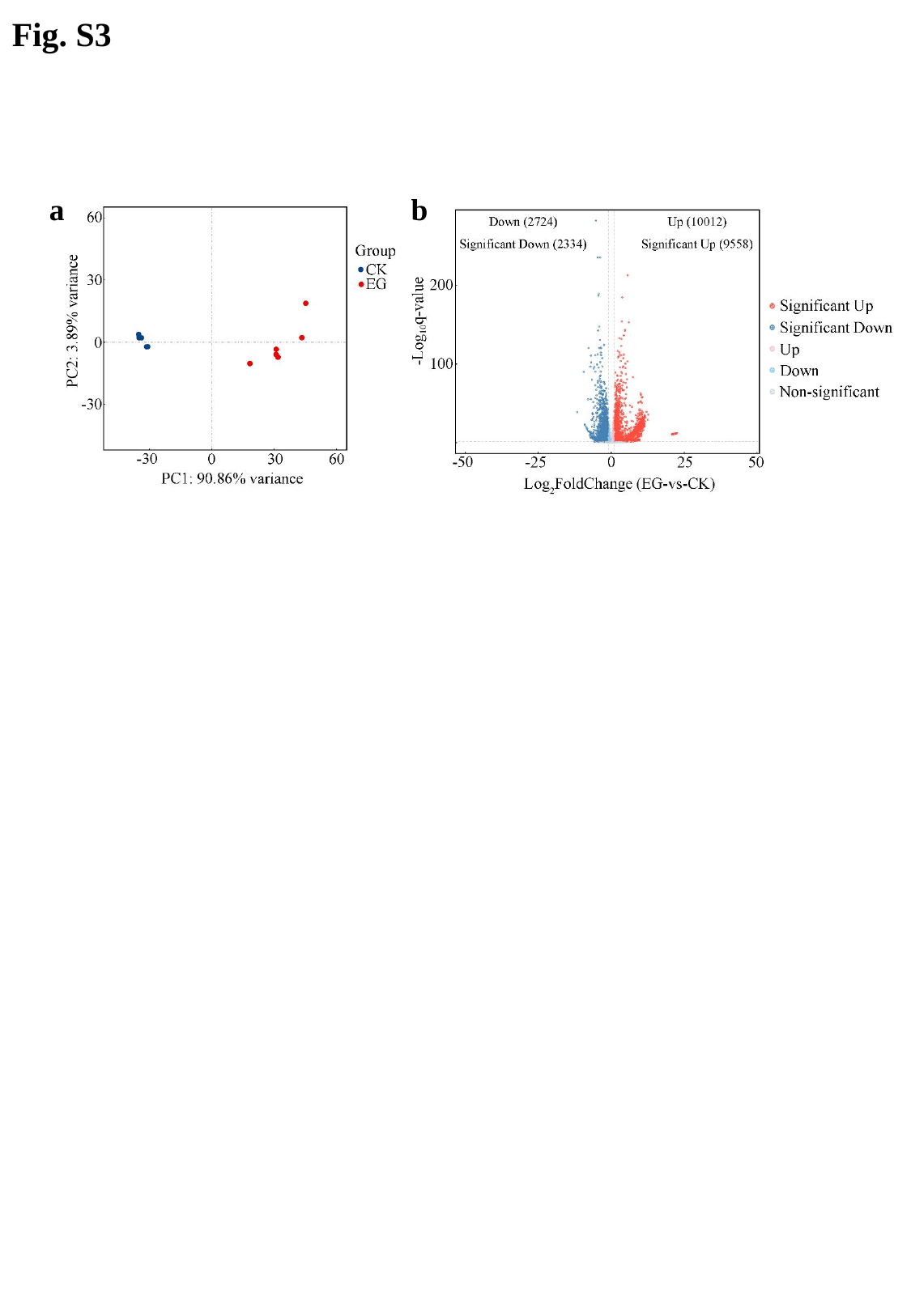

Fig. S3
a
b

## Slide 6
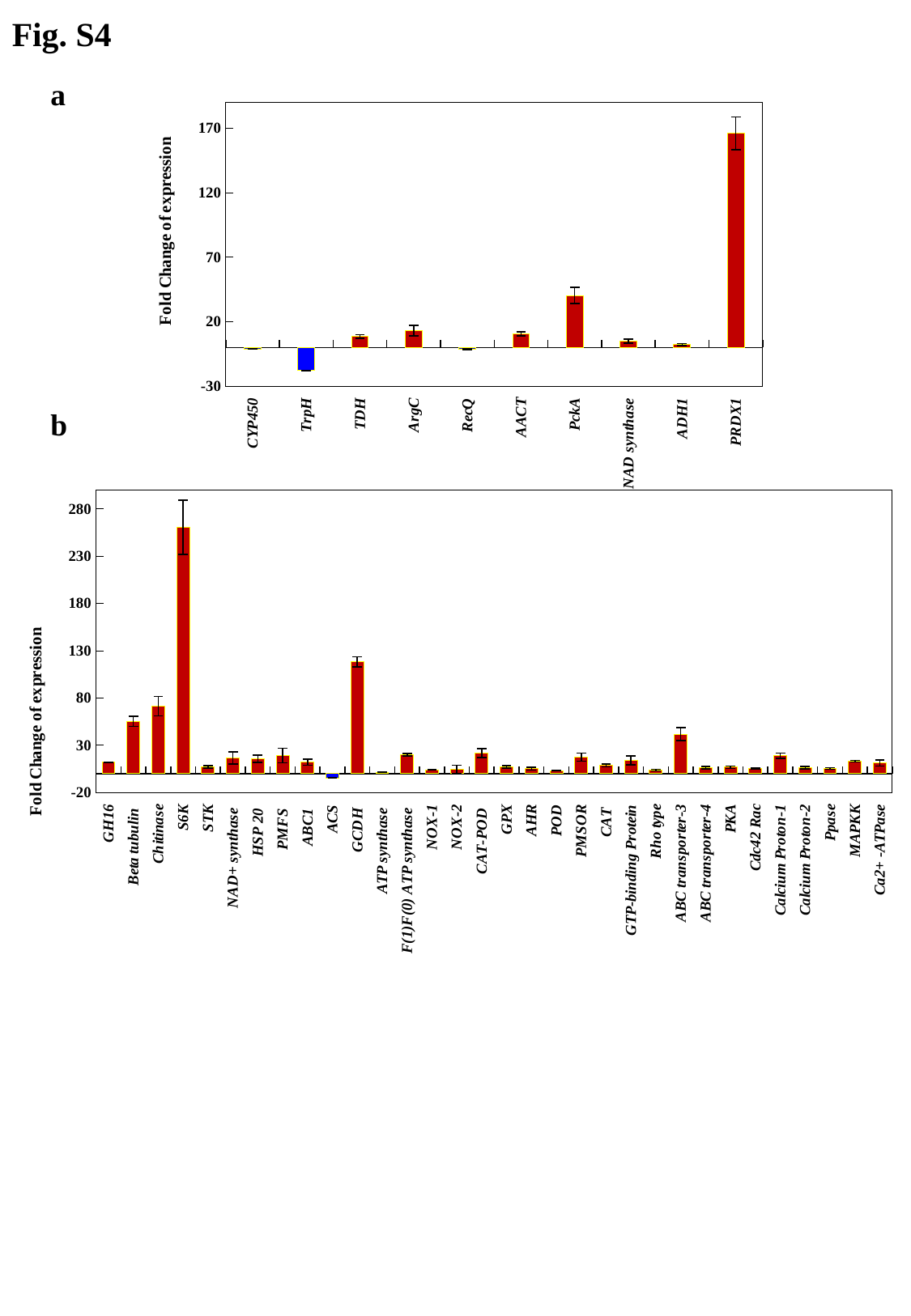

Fig. S4
a
### Chart
| Category | |
|---|---|
| CYP450 | -1.05048069608986 |
| TrpH | -17.9192550759397 |
| TDH | 8.51375268780557 |
| ArgC | 13.1071435121809 |
| RecQ | -1.36275442337687 |
| AACT | 10.5989112246503 |
| PckA | 40.2989015675887 |
| NAD synthase | 4.88211246924389 |
| ADH1 | 2.25590604056081 |
| PRDX1 | 166.120432995972 |b
### Chart
| Category | |
|---|---|
| GH16 | 12.1078875662004 |
| Beta tubulin | 55.2927647224035 |
| Chitinase | 71.3406224720427 |
| S6K | 260.573810969465 |
| STK | 7.29084464515721 |
| NAD+ synthase | 16.6619262752446 |
| HSP 20 | 15.7188524476773 |
| PMFS | 19.212728627544 |
| ABC1 | 12.0833551048505 |
| ACS | -4.4307213749259 |
| GCDH | 118.130315804311 |
| ATP synthase | 1.46514290241556 |
| F(1)F(0) ATP synthase | 20.0124219985552 |
| NOX-1 | 4.14297754968228 |
| NOX-2 | 4.66697293290607 |
| CAT-POD | 21.5882912567352 |
| GPX | 7.17492660664903 |
| AHR | 5.40266880327287 |
| POD | 3.28052559018027 |
| PMSOR | 17.6435969030512 |
| CAT | 8.81203850906447 |
| GTP-binding Protein | 14.0254827907148 |
| Rho type | 3.68275495892795 |
| ABC transporter-3 | 41.7113555507773 |
| ABC transporter-4 | 6.48922001203956 |
| PKA | 6.81838434655346 |
| Cdc42 Rac | 5.58210409042516 |
| Calcium Proton-1 | 18.8743353791309 |
| Calcium Proton-2 | 6.32943648490205 |
| Ppase | 5.27946500039556 |
| MAPKK | 13.2882894653977 |
| Ca2+ -ATPase | 11.1961942903944 |

## Slide 7
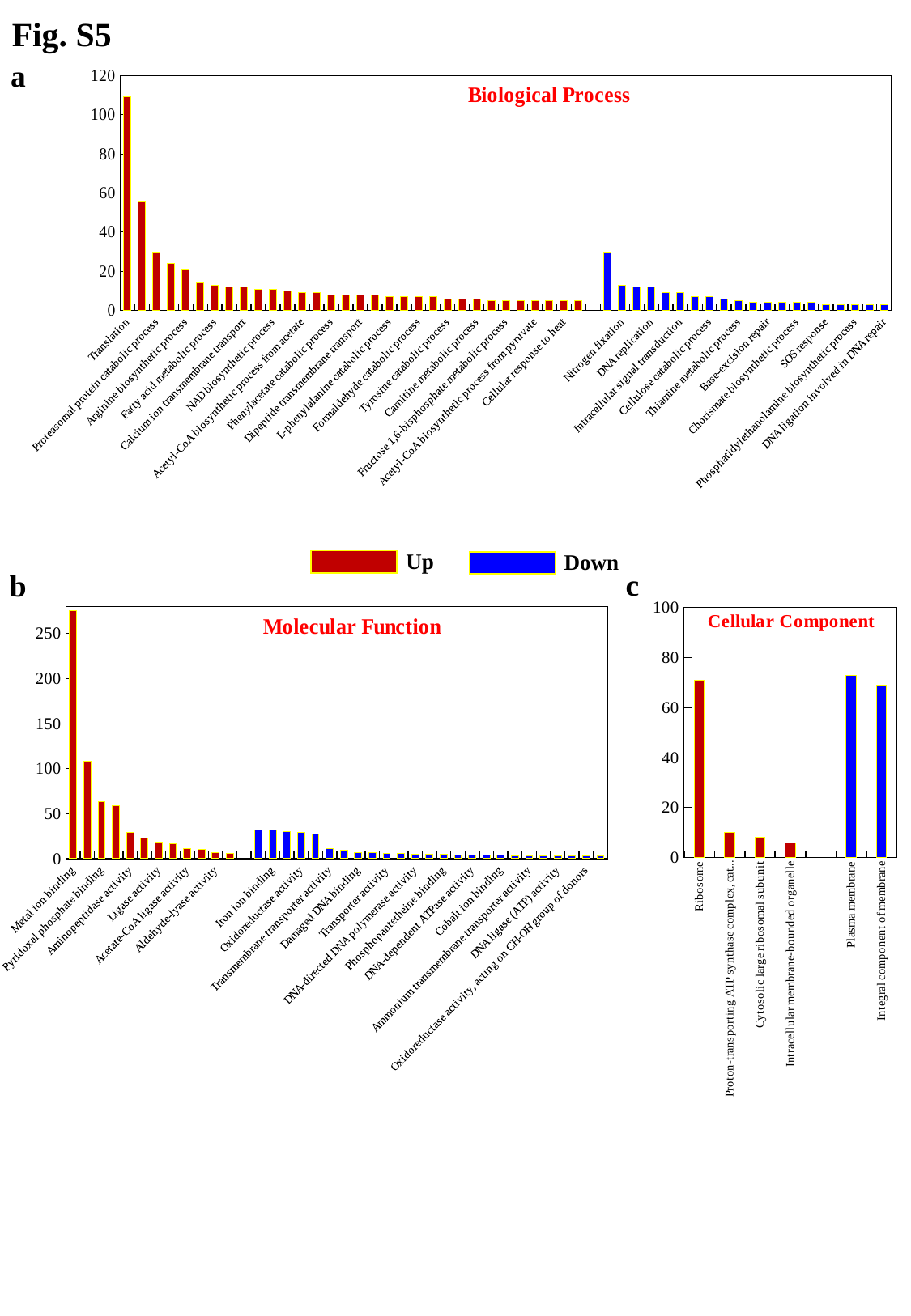

Fig. S5
a
### Chart: Biological Process
| Category | |
|---|---|
| Translation | 109.0 |
| Protein folding | 56.0 |
| Proteasomal protein catabolic process | 30.0 |
| Protein ubiquitination | 24.0 |
| Arginine biosynthetic process | 21.0 |
| Response to oxidative stress | 14.0 |
| Fatty acid metabolic process | 13.0 |
| Menaquinone biosynthetic process | 12.0 |
| Calcium ion transmembrane transport | 12.0 |
| ATP synthesis coupled proton transport | 11.0 |
| NAD biosynthetic process | 11.0 |
| Riboflavin biosynthetic process | 10.0 |
| Acetyl-CoA biosynthetic process from acetate | 9.0 |
| Purine nucleobase metabolic process | 9.0 |
| Phenylacetate catabolic process | 8.0 |
| Pectin catabolic process | 8.0 |
| Dipeptide transmembrane transport | 8.0 |
| Photosynthesis | 8.0 |
| L-phenylalanine catabolic process | 7.0 |
| Catechol-containing compound metabolic process | 7.0 |
| Formaldehyde catabolic process | 7.0 |
| Gamma-aminobutyric acid catabolic process | 7.0 |
| Tyrosine catabolic process | 6.0 |
| Valine catabolic process | 6.0 |
| Carnitine metabolic process | 6.0 |
| Cytoplasmic translation | 5.0 |
| Fructose 1,6-bisphosphate metabolic process | 5.0 |
| Formate catabolic process | 5.0 |
| Acetyl-CoA biosynthetic process from pyruvate | 5.0 |
| Lipid biosynthetic process | 5.0 |
| Cellular response to heat | 5.0 |
| Beta-ketoadipate pathway | 5.0 |
| | None |
| Transmembrane transport | 30.0 |
| Nitrogen fixation | 13.0 |
| DNA repair | 12.0 |
| DNA replication | 12.0 |
| Cyclic nucleotide biosynthetic process | 9.0 |
| Intracellular signal transduction | 9.0 |
| DNA recombination | 7.0 |
| Cellulose catabolic process | 7.0 |
| Mismatch repair | 6.0 |
| Thiamine metabolic process | 5.0 |
| Unsaturated fatty acid biosynthetic process | 4.0 |
| Base-excision repair | 4.0 |
| Aromatic amino acid family biosynthetic process | 4.0 |
| Chorismate biosynthetic process | 4.0 |
| Diaminopimelate biosynthetic process | 4.0 |
| SOS response | 3.0 |
| Toxin biosynthetic process | 3.0 |
| Phosphatidylethanolamine biosynthetic process | 3.0 |
| DNA-dependent DNA replication | 3.0 |
| DNA ligation involved in DNA repair | 3.0 |Up
Down
c
b
### Chart: Molecular Function
| Category | |
|---|---|
| Metal ion binding | 275.0 |
| Structural constituent of ribosome | 108.0 |
| Pyridoxal phosphate binding | 63.0 |
| NAD binding | 59.0 |
| Aminopeptidase activity | 29.0 |
| Calcium-transporting ATPase activity | 23.0 |
| Ligase activity | 18.0 |
| Proton-transporting ATP synthase activity, rotational mechanism | 17.0 |
| Acetate-CoA ligase activity | 11.0 |
| AMP binding | 10.0 |
| Aldehyde-lyase activity | 7.0 |
| Transferase activity, transferring hexosyl groups | 6.0 |
| | None |
| Heme binding | 32.0 |
| Iron ion binding | 32.0 |
| Monooxygenase activity | 30.0 |
| Oxidoreductase activity | 29.0 |
| Oxidoreductase activity, acting on paired donors, with incorporation or reduction of molecular oxygen | 27.0 |
| Transmembrane transporter activity | 11.0 |
| Phosphorus-oxygen lyase activity | 9.0 |
| Damaged DNA binding | 7.0 |
| Cholinesterase activity | 7.0 |
| Transporter activity | 6.0 |
| Non-membrane spanning protein tyrosine kinase activity | 6.0 |
| DNA-directed DNA polymerase activity | 5.0 |
| Cellulase activity | 5.0 |
| Phosphopantetheine binding | 5.0 |
| Delta12-fatty-acid desaturase activity | 4.0 |
| DNA-dependent ATPase activity | 4.0 |
| Choline dehydrogenase activity | 4.0 |
| Cobalt ion binding | 4.0 |
| 8-oxo-7,8-dihydroguanine DNA N-glycosylase activity | 3.0 |
| Ammonium transmembrane transporter activity | 3.0 |
| Phosphatidylserine decarboxylase activity | 3.0 |
| DNA ligase (ATP) activity | 3.0 |
| L-iditol 2-dehydrogenase activity | 3.0 |
| Oxidoreductase activity, acting on CH-OH group of donors | 3.0 |
| Mismatched DNA binding | 3.0 |
### Chart: Cellular Component
| Category | |
|---|---|
| Ribosome | 71.0 |
| Proton-transporting ATP synthase complex, catalytic core F (1) | 10.0 |
| Cytosolic large ribosomal subunit | 8.0 |
| Intracellular membrane-bounded organelle | 6.0 |
| | None |
| Plasma membrane | 73.0 |
| Integral component of membrane | 69.0 |

## Slide 8
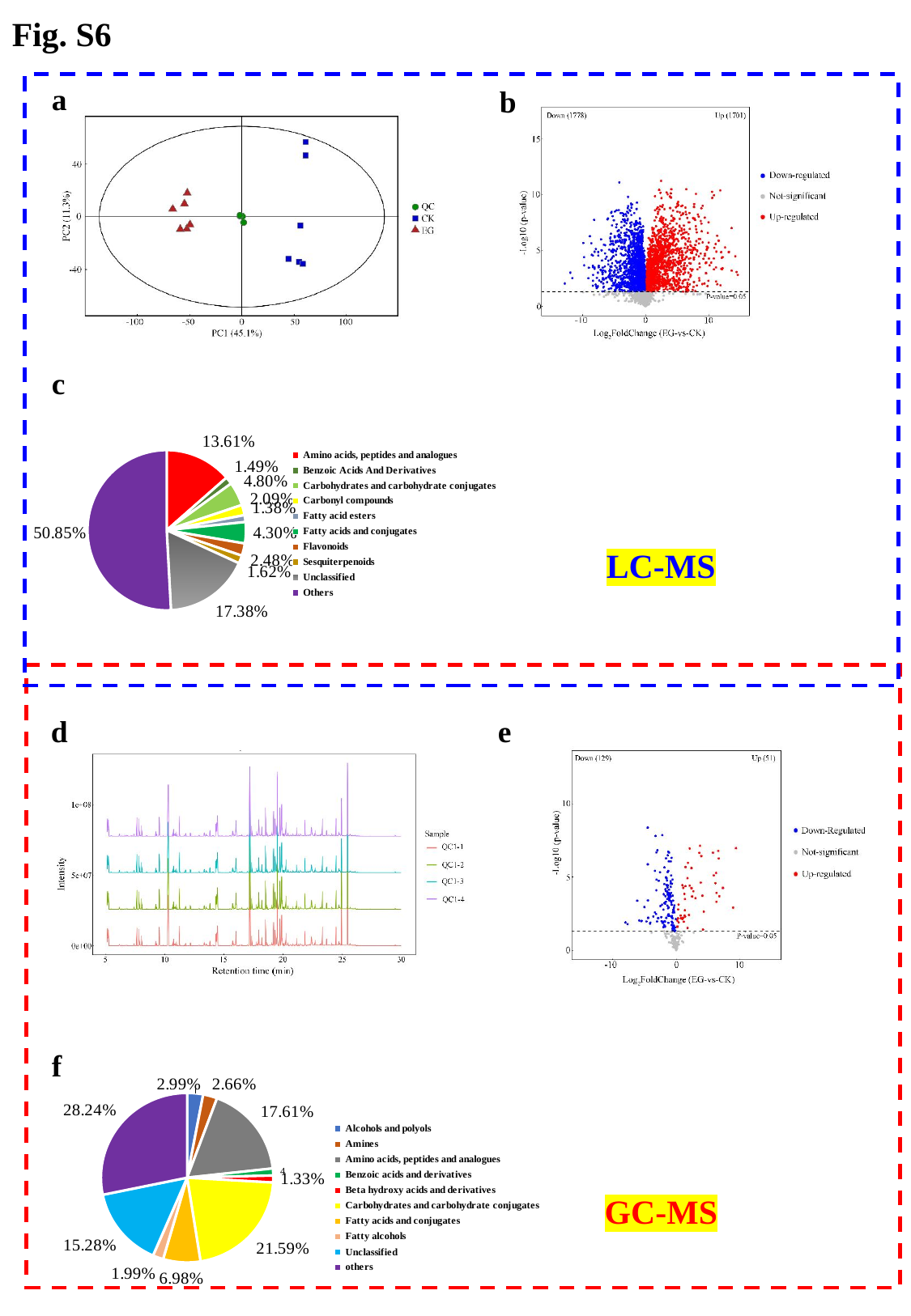

Fig. S6
a
b
c
### Chart
| Category | |
|---|---|
| Amino acids, peptides and analogues | 757.0 |
| Benzoic Acids And Derivatives | 83.0 |
| Carbohydrates and carbohydrate conjugates | 267.0 |
| Carbonyl compounds | 116.0 |
| Fatty acid esters | 77.0 |
| Fatty acids and conjugates | 239.0 |
| Flavonoids | 138.0 |
| Sesquiterpenoids | 90.0 |
| Unclassified | 967.0 |
| Others | 2829.0 |LC-MS
e
d
f
### Chart
| Category | |
|---|---|
| Alcohols and polyols | 9.0 |
| Amines | 8.0 |
| Amino acids, peptides and analogues | 53.0 |
| Benzoic acids and derivatives | 4.0 |
| Beta hydroxy acids and derivatives | 4.0 |
| Carbohydrates and carbohydrate conjugates | 65.0 |
| Fatty acids and conjugates | 21.0 |
| Fatty alcohols | 6.0 |
| Unclassified | 46.0 |
| others | 85.0 |GC-MS

## Slide 9
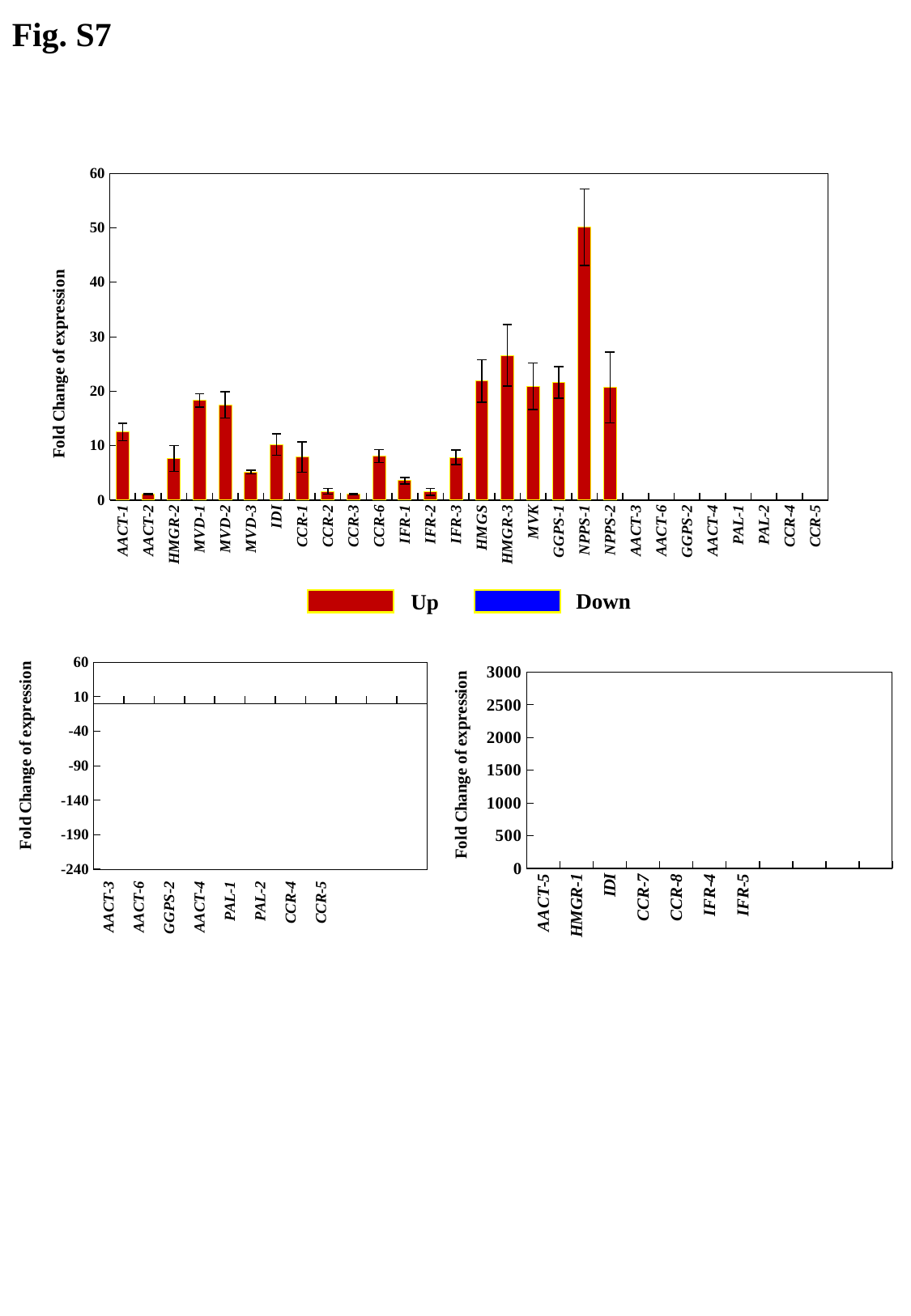

Fig. S7
[unsupported chart]
Down
Up
### Chart
| Category | |
|---|---|
| AACT-3 | -6.06407699837825 |
| AACT-6 | -6.5185077308569 |
| GGPS-2 | -7.90978970354821 |
| AACT-4 | -235.51754054233 |
| PAL-1 | -1.42674809958241 |
| PAL-2 | -11.7162778407913 |
| CCR-4 | -6.88721928994978 |
| CCR-5 | -1.49061911399777 |
### Chart
| Category | |
|---|---|
| AACT-5 | 2012.44565029163 |
| HMGR-1 | 211.033892760385 |
| IDI | 2160.99913870324 |
| CCR-7 | 130.853837423802 |
| CCR-8 | 178.558127049918 |
| IFR-4 | 166.45050589978 |
| IFR-5 | 322.780588017467 |

## Slide 10
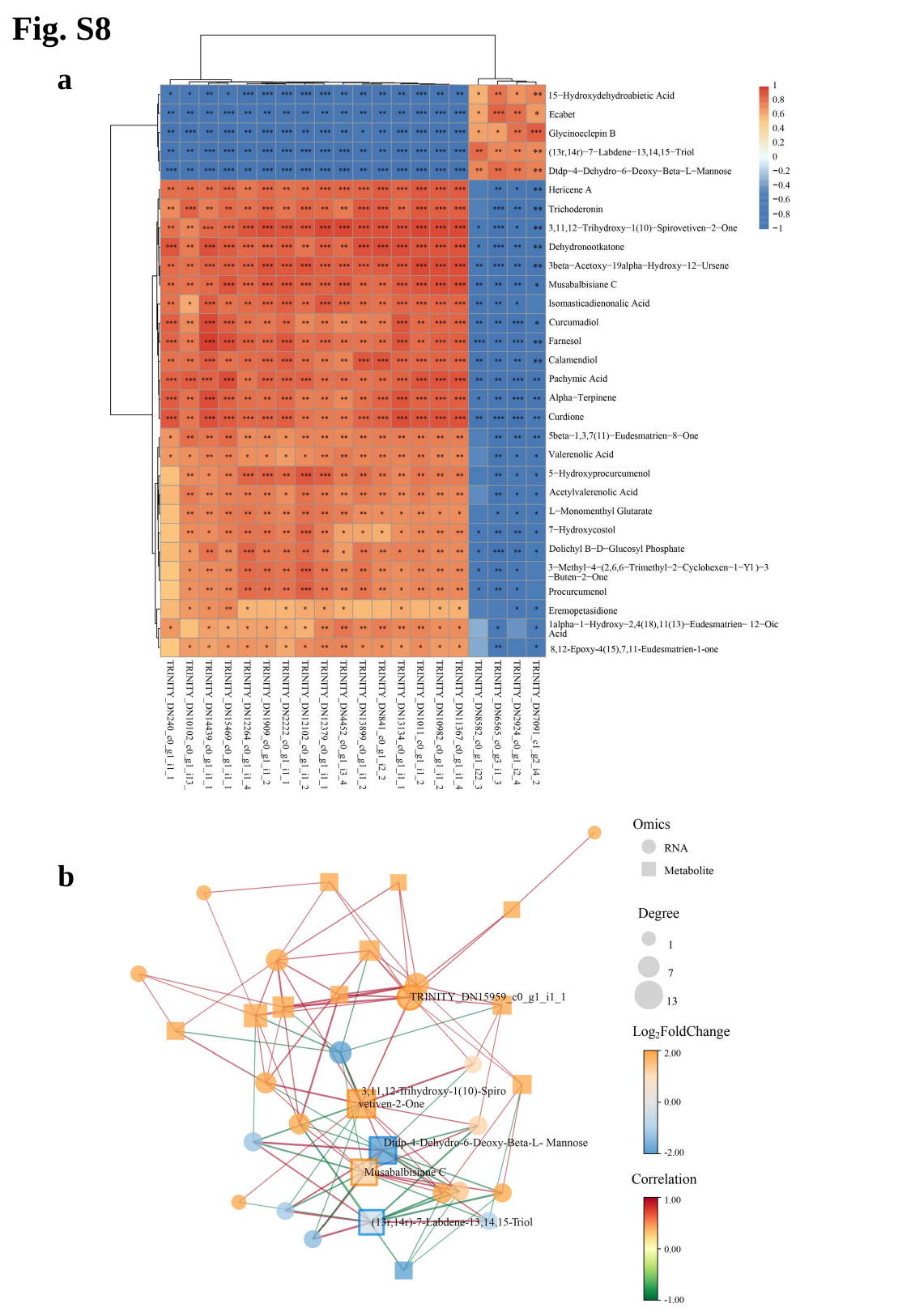

Fig. S8
a
b

## Slide 11
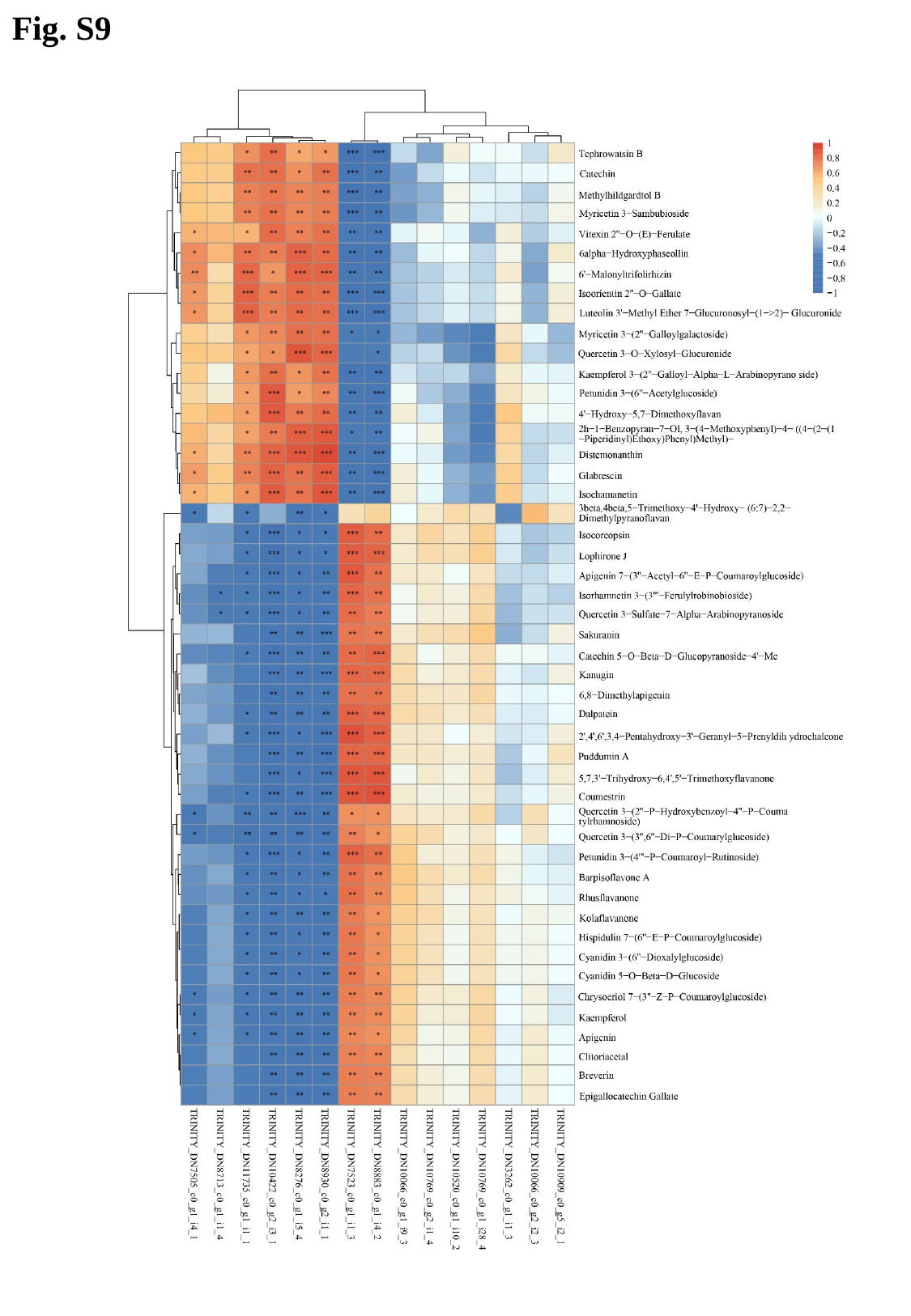

Fig. S9

## Slide 12
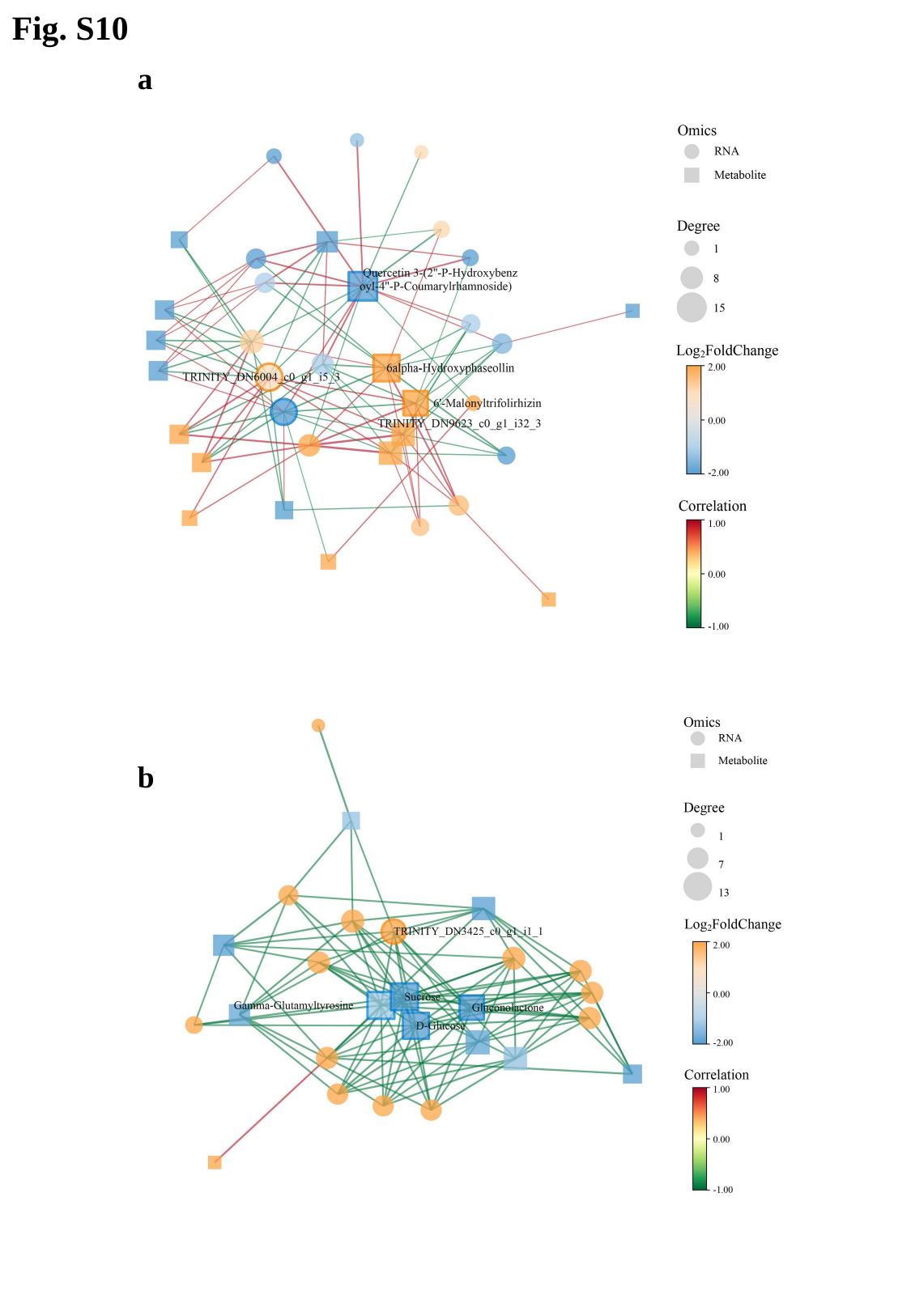

Fig. S10
a
b
