# Supplementary material for: Fungal-fungal interaction between Sanghuangporus vaninii and its endophytic Fusarium solani rewires host secondary metabolism to boost bioactive metabolite production
Source: Microb Cell Fact. 2026 Apr 4;25:135. doi: 10.1186/s12934-026-02994-z (PMC13214342; doi:10.1186/s12934-026-02994-z)
Supplement: Supplementary file 2 — Supplementary Material 2. (See in Supplementary Word). Table S2. Output statistics and quality assessment of RNA sequencing data for S. vaninii MF5 samples from control and co-culture with F. solani MF20 groups. Table S3. Summary statistics of unigene annotation for the S. vaninii MF5 transcriptome assembly against various public databases. Table S8. List and annotation of DEGs involved in the developmental process and hyphal morphogenesis of S. vaninii MF5 induced by F. solani MF20. Table S9. List and annotation of DEGs related to cell membrane permeabilization and fatty acid metabolism in S. vaninii MF5 induced by F. solani MF20. Table S10. List and annotation of DEGs involved in ATP metabolism and calcium signaling/transport in S. vaninii MF5 induced by F. solani MF20. Table S11. List and annotation of DEGs involved in oxidative stress response and signal transduction in S. vaninii MF5 induced by F. solani MF20. Table S15. Top 30 most significantly up-regulated differential metabolites (DMs) in S. vaninii MF5 induced by co-culture with F. solani MF20. Table S16. List and annotation of DEGs involved in the terpenoid backbone biosynthesis pathway of S. vaninii MF5 induced by F. solani MF20. Table S17. List of differential metabolites (DMs) identified as terpenoids in S. vaninii MF5 induced by co-culture with F. solani MF20. Table S19. List of differential metabolites (DMs) identified as flavonoids and isoflavonoids in S. vaninii MF5 induced by co-culture with F. solani MF20. Table S20. List and annotation of DEGs potentially involved in flavonoid and isoflavonoid biosynthesis in S. vaninii MF5 induced by F. solani MF20. Table S23. List of differential metabolites (DMs) related to polysaccharide and central carbon metabolism in S. vaninii MF5 induced by co-culture with F. solani MF20 [file 12934_2026_2994_MOESM2_ESM.docx]

**Additional file 2**

**Table S2.** Output statistics and quality assessment of RNA sequencing data for *S. vaninii* MF5 samples from control and co-culture with *F. solani* MF20 groups.

| **Attributes** | **Control** | | | | | | **Co-culture** | | | | | |
| --- | --- | --- | --- | --- | --- | --- | --- | --- | --- | --- | --- | --- |
| Sample | CK1 | CK2 | CK3 | CK4 | CK5 | CK6 | T1 | T2 | T3 | T4 | T5 | T6 |
| CleanReads (M) | 49.81 | 47.61 | 48.30 | 49.29 | 50.87 | 50.96 | 50.94 | 47.94 | 48.42 | 48.54 | 48.33 | 50.26 |
| CleanBases (G) | 7.23 | 6.96 | 7.05 | 7.17 | 7.41 | 7.44 | 7.45 | 7.02 | 7.02 | 7.09 | 6.97 | 7.35 |
| ValidBases (%) | 96.52 | 97.2 | 97.04 | 96.77 | 96.85 | 97.08 | 97.27 | 97.35 | 96.41 | 97.12 | 95.81 | 97.18 |
| Q30 (%) | 96.3 | 95.88 | 96.06 | 95.39 | 96.18 | 95.87 | 96.19 | 95.8 | 96.28 | 95.95 | 96.36 | 94.78 |
| GC (%) | 51.6 | 51.6 | 51.29 | 51.56 | 51.64 | 51.5 | 51.48 | 51.53 | 51.48 | 51.7 | 51.42 | 51.65 |
| CleanData (G) | 86.16 G | | | | | | | | | | | |
| Total unigenes | 25,373 | | | | | | | | | | | |
| Max length | 15,026 | | | | | | | | | | | |
| Min length | 301 | | | | | | | | | | | |
| Average length | 1,470.87 | | | | | | | | | | | |

**Table S3.** Summary statistics of unigene annotation for the *S. vaninii* MF5 transcriptome assembly against various public databases.

| **Annotation** | **Database** | | | | |  |  |
| --- | --- | --- | --- | --- | --- | --- | --- |
|  | **NR** | **Swiss-Prot** | **KEGG** | **KOG** | **eggNOG** | **GO** | **Pfam** |
| **Numbers** | 11,437 | 5,563 | 2,977 | 10,209 | 18,205 | 5,177 | 12,068 |
| **Ratio (%)** | 45.08 | 21.92 | 11.73 | 40.24 | 71.75 | 20.40 | 47.56 |

**Table S8.** List and annotation of DEGs involved in the developmental process and hyphal morphogenesis of *S. vaninii* MF5 induced by *F. solani* MF20.

| **Unigene ID** | **Up/down** | **Fold Change** | **Description** |
| --- | --- | --- | --- |
| Developmental process | | | |
| TRINITY_DN1022_c0_g1_i1_3 | Up | Inf | Ras-related small GTPase, Rho type [KOG0393] |
| TRINITY_DN10861_c0_g1_i1_2 | Up | Inf | Ras-related small GTPase, Rho type [KOG0393] |
| TRINITY_DN11645_c0_g1_i1_2 | Up | 158.73 | Ras-related GTPase [KOG0395] |
| TRINITY_DN12798_c0_g1_i1_4 | Up | Inf | Ras-related GTPase [KOG0395] |
| TRINITY_DN1472_c0_g2_i1_1 | Up | 59.19 | Ras-related GTPase [KOG0395] |
| TRINITY_DN15515_c0_g1_i1_1 | Up | Inf | Ras-related GTPase [KOG0395] |
| TRINITY_DN15589_c0_g1_i1_1 | Up | Inf | Ras-related GTPase [KOG0395] |
| TRINITY_DN15607_c0_g1_i1_1 | Up | Inf | Ras-related GTPase [KOG0395] |
| TRINITY_DN2204_c0_g1_i2_4 | Up | 227.56 | Ras-related small GTPase, Rho type [KOG0393] |
| TRINITY_DN2464_c0_g1_i2_4 | Up | Inf | Ras-related small GTPase, Rho type [KOG0393] |
| TRINITY_DN2911_c0_g1_i1_1 | Up | 175.71 | Ras-related small GTPase, Rho type [KOG0393] |
| TRINITY_DN3889_c0_g1_i2_2 | Up | 955.80 | Ras-related small GTPase, Rho type [KOG0393] |
| TRINITY_DN6970_c0_g1_i2_1 | Up | 2.13 | Ras-related small GTPase, Rho type [KOG0393] |
| TRINITY_DN770_c0_g1_i1_1 | Up | Inf | Ras-related GTPase [KOG0395] |
| TRINITY_DN11008_c0_g1_i1_2 | Up | Inf | Glycosyl hydrolases family 16, GH16 [PF00722.21] |
| TRINITY_DN12613_c0_g1_i1_4 | Up | Inf | Glycosyl hydrolases family 28, GH28 [PF00295.17] |
| TRINITY_DN8930_c0_g1_i11_3 | Up | 2.44 | Glycosyl hydrolases family 28, GH28 [PF00295.17] |
| TRINITY_DN14306_c0_g1_i1_4 | Up | Inf | Beta tubulin [KOG1375] |
| TRINITY_DN3388_c0_g1_i3_2 | Up | 1722.63 | Beta tubulin [KOG1375] |
| TRINITY_DN10956_c0_g1_i1_4 | Up | Inf | Chitinase [KOG2806] |
| TRINITY_DN10960_c0_g1_i1_2 | Up | 250.82 | Chitinase [KOG2806] |
| TRINITY_DN15023_c0_g1_i1_4 | Up | Inf | Chitinase [KOG2806] |
| TRINITY_DN10253_c0_g1_i25_2 | Up | 2.61 | Histone acetyltransferase (MYST family) [KOG2747] |
| TRINITY_DN11527_c0_g1_i1_4 | Up | Inf | Ribosomal protein S6 kinase and related proteins, S6K [KOG0598] |
| TRINITY_DN11940_c0_g1_i1_1 | Up | Inf | Ribosomal protein S6 kinase and related proteins, S6K [KOG0598] |
| TRINITY_DN12158_c0_g1_i1_4 | Up | 70.13 | Ribosomal protein S6 kinase and related proteins, S6K [KOG0598] |
| TRINITY_DN1417_c0_g1_i1_2 | Up | Inf | Ribosomal protein S6 kinase and related proteins, S6K [KOG0598] |
| TRINITY_DN15390_c0_g1_i1_1 | Up | Inf | Ribosomal protein S6 kinase and related proteins, S6K [KOG0598] |
| TRINITY_DN2202_c0_g1_i1_1 | Up | Inf | Ribosomal protein S6 kinase and related proteins, S6K [KOG0598] |
| TRINITY_DN11747_c0_g1_i1_4 | Up | Inf | Putative serine/threonine protein kinase [KOG0610] |
| TRINITY_DN12079_c0_g1_i1_2 | Up | Inf | Serine/threonine protein kinase, Ser/Thr PK [KOG0694] |
| TRINITY_DN12424_c0_g1_i1_4 | Up | Inf | Serine/threonine protein kinase, Ser/Thr PK [KOG0583] |
| TRINITY_DN12471_c0_g1_i1_2 | Up | Inf | Serine/threonine protein kinase, Ser/Thr PK [KOG0580] |
| TRINITY_DN13151_c0_g1_i1_4 | Up | Inf | Serine/threonine protein kinase, Ser/Thr PK [KOG0583] |
| TRINITY_DN1388_c0_g1_i1_1 | Up | Inf | Serine/threonine protein kinase Chk2 and related proteins [KOG0615] |
| TRINITY_DN2082_c0_g1_i1_1 | Up | Inf | Serine/threonine protein kinase, Ser/Thr PK [KOG0694] |
| TRINITY_DN2471_c0_g1_i1_1 | Up | Inf | Serine/threonine protein kinase, Ser/Thr PK [KOG0583] |
| TRINITY_DN10855_c0_g1_i1_2 | Up | Inf | Sporulation resulting in formation of a cellular spore [GO:0030435] |
| TRINITY_DN12899_c0_g1_i1_1 | Up | Inf | NAD+ synthase [38/111(34.23)] |
| TRINITY_DN13278_c0_g1_i1_3 | Up | Inf | Heat shock protein, HSP 20 [ENOG4111YTA] |

**Table S9.** List and annotation of DEGs related to cell membrane permeabilization and fatty acid metabolism in *S. vaninii* MF5 induced by *F. solani* MF20.

| **Unigene ID** | **Up/Down** | **Fold Change** | **Description** |
| --- | --- | --- | --- |
| Cell membrane permeabilization | | | |
| TRINITY_DN10036_c0_g1_i3_3 | Up | 6.63 | ABC transporter ATP-binding/permease protein, ABC1 [O65934\|ABC1_MYCTU] |
| TRINITY_DN10367_c0_g1_i18_1 | Up | 20.54 | ABC transporter ATP-binding/permease protein, ABC1 [O65934\|ABC1_MYCTU] |
| TRINITY_DN10752_c0_g3_i3_4 | Up | 2.75 | ABC transporter ATP-binding/permease protein, ABC1 [O65934\|ABC1_MYCTU] |
| TRINITY_DN10936_c0_g1_i1_2 | Up | Inf | Metal ABC transporter permease [SAR116 cluster bacterium] [MAB18248.1] |
| TRINITY_DN10956_c0_g1_i1_2 | Up | Inf | ABC transporter ATP-binding/permease protein [O65934\|ABC1_MYCTU] |
| TRINITY_DN11584_c0_g1_i1_4 | Up | Inf | ABC transporter ATP-binding/permease protein [O65934\|ABC1_MYCTU] |
| TRINITY_DN15150_c0_g1_i1_4 | Up | 446.25 | Metal ABC transporter permease [SAR116 cluster bacterium][MAB18248.1] |
| TRINITY_DN15490_c0_g1_i1_1 | Up | Inf | ABC transporter ATP-binding/permease protein [O65934\|ABC1_MYCTU] |
| TRINITY_DN10375_c0_g1_i17_2 | Inf | 7.20 | Permease of the major facilitator superfamily, PMFS [KOG2533] |
| TRINITY_DN10475_c0_g1_i1_3 | Up | Inf | Permease of the major facilitator superfamily, PMFS [KOG2533] |
| TRINITY_DN10560_c0_g1_i4_1 | Up | 2.16 | Permease of the major facilitator superfamily, PMFS [KOG2533] |
| TRINITY_DN10676_c0_g1_i1_3 | Up | 26.26 | Permease of the major facilitator superfamily, PMFS [KOG2533] |
| TRINITY_DN10936_c0_g1_i1_4 | Up | Inf | Permease of the major facilitator superfamily, PMFS [KOG2533] |
| TRINITY_DN10986_c0_g1_i1_1 | Up | 6.15 | Permease of the major facilitator superfamily, PMFS [KOG2533] |
| TRINITY_DN11884_c0_g1_i1_3 | Up | Inf | Permease of the major facilitator superfamily, PMFS [KOG2533] |
| TRINITY_DN13895_c0_g1_i1_4 | Up | 115.69 | Permease of the major facilitator superfamily, PMFS [KOG2533] |
| TRINITY_DN14254_c0_g1_i1_4 | Up | Inf | Permease of the major facilitator superfamily, PMFS [KOG2533] |
| TRINITY_DN101_c0_g1_i1_1 | Up | Inf | Amino acid permease [WP_099223559.1] |
| TRINITY_DN11959_c0_g1_i1_2 | Up | Inf | Ammonia permease [KOG0682] |
| TRINITY_DN12960_c0_g1_i1_4 | Up | Inf | Ammonia permease [KOG0682] |
| TRINITY_DN1694_c0_g2_i1_3 | Up | 3323.81 | Amino acid permease [WP_113657938.1] |
| TRINITY_DN2390_c0_g1_i1_1 | Up | 444.47 | Amino acid permease [PF00324.21] |
| TRINITY_DN468_c0_g1_i1_1 | Up | 937.77 | Amino acid permease [WP_122919845.1] |
| TRINITY_DN10845_c0_g1_i1_3 | Up | Inf | Permease for cytosine/purines, uracil, thiamine, allantoin [PF02133.15] |
| TRINITY_DN13769_c0_g1_i1_1 | Up | Inf | Permease for cytosine/purines, uracil, thiamine, allantoin [PF02133.15] |
| TRINITY_DN14712_c0_g1_i1_4 | Up | Inf | Permease for cytosine/purines, uracil, thiamine, allantoin [PF02133.15] |
| TRINITY_DN13772_c0_g1_i1_4 | Up | Inf | Gamma-aminobutyrate permease [EPZ52880.1] |
| TRINITY_DN14118_c0_g1_i1_1 | Up | Inf | Lysine-specific permease [P25737\|LYSP_ECOLI] |
| TRINITY_DN15471_c0_g1_i1_4 | Up | Inf | Lysine-specific permease [P25737\|LYSP_ECOLI] |
| TRINITY_DN14126_c0_g1_i1_2 | Up | Inf | Uracil permease [ARB01809.1] |
| TRINITY_DN1531_c0_g1_i1_3 | Up | Inf | Sugar phosphate permease [RKR75173.1] |
| Fatty acid related enzyme | | | |
| TRINITY_DN10605_c0_g1_i17_4 | Down | -2.03 | Acyl-CoA synthetase, ACS [OJV93677.1] |
| TRINITY_DN1045_c0_g1_i1_4 | Up | 136.81 | Glutaryl-CoA dehydrogenase, GCDH [194/320(60.62)] |
| TRINITY_DN10936_c0_g2_i1_1 | Up | 4.10 | Enoyl-CoA hydratase [108/224(48.21)] |
| TRINITY_DN11290_c0_g1_i1_2 | Up | Inf | Δ3-Δ2-enoyl-CoA isomerase [75/215(34.88)] |
| TRINITY_DN11965_c0_g1_i1_1 | Up | Inf | Enoyl-CoA hydratase [108/224(48.21)] |
| TRINITY_DN170_c0_g1_i1_3 | Up | 575.78 | Δ12-fatty-acid desaturase [Q54794\|DESA_ARTPT] |

**Table S10.** List and annotation of DEGs involved in ATP metabolism and calcium signaling/transport in *S. vaninii* MF5 induced by *F. solani* MF20.

| **Unigene ID** | **Up/Down** | **Fold Change** | **Description** |
| --- | --- | --- | --- |
| ATP reated enzyme | | | |
| TRINITY_DN10187_c0_g1_i7_1 | Up | 2.02 | F0F1-type ATP synthase [KOG1353] |
| TRINITY_DN10967_c0_g1_i1_3 | Up | 1148.16 | ATP synthase [PF02823.16] |
| TRINITY_DN14667_c0_g1_i1_2 | Up | 2.00 | ATP synthase [PF00213.18] |
| TRINITY_DN14943_c0_g1_i1_2 | Inf | 459.70 | ATP synthase [PF00213.18] |
| TRINITY_DN158_c0_g1_i1_1 | Up | 3.04 | ATP synthase [PF00213.18] |
| TRINITY_DN1691_c0_g1_i2_2 | Up | 577.55 | ATP synthase [PF00231.19] |
| TRINITY_DN2193_c0_g1_i1_2 | Up | 1386.99 | ATP synthase [PF00006.25] |
| TRINITY_DN2863_c0_g1_i3_1 | Up | 1443.22 | ATP synthase [PF00006.25] |
| TRINITY_DN3839_c0_g1_i1_3 | Up | 2.67 | ATP synthase [PF02823.16] |
| TRINITY_DN5082_c0_g1_i2_1 | Up | 3.07 | ATP synthase [PF02823.16] |
| TRINITY_DN12771_c0_g1_i1_3 | Up | 2428.08 | F(1)F(0) ATP synthase [COG0636] |
| TRINITY_DN515_c0_g1_i1_1 | Up | 3.29 | F(1)F(0) ATP synthase [COG0636] |
| Calcium related enzyme | | | |
| TRINITY_DN11665_c0_g1_i1_3 | Up | Inf | Calcium-dependent protein kinase [ENOG410XRMJ] |
| TRINITY_DN13136_c0_g1_i1_4 | Up | Inf | Calcium-dependent protein kinase [ENOG410XRMJ] |
| TRINITY_DN1956_c0_g2_i1_3 | Up | Inf | Calcium-dependent protein kinase [ENOG410XRMJ] |
| TRINITY_DN2980_c0_g1_i2_1 | Up | Inf | Calcium-dependent protein kinase [ENOG410XRMJ] |
| TRINITY_DN1072_c0_g1_i1_2 | Up | Inf | Calcium Proton [COG0387] |
| TRINITY_DN11751_c0_g1_i1_2 | Up | Inf | Calcium Proton [COG0387] |
| TRINITY_DN1250_c0_g1_i1_4 | Up | Inf | Calcium Proton [COG0387] |
| TRINITY_DN12837_c0_g1_i1_2 | Up | Inf | Calcium Proton [COG0387] |
| TRINITY_DN15028_c0_g1_i1_4 | Up | Inf | Calcium Proton [COG0387] |
| TRINITY_DN15499_c0_g1_i1_1 | Up | Inf | Calcium Proton [COG0387] |
| TRINITY_DN2314_c0_g1_i1_1 | Up | Inf | Calcium Proton [COG0387] |
| TRINITY_DN4302_c0_g1_i7_4 | Up | Inf | Calcium Proton [COG0387] |
| TRINITY_DN4845_c0_g1_i1_2 | Up | Inf | Calcium Proton [COG0387] |
| TRINITY_DN5534_c1_g1_i10_1 | Up | Inf | Calcium Proton [COG0387] |
| TRINITY_DN11869_c0_g1_i1_1 | Up | Inf | Ca^2+^  transporting ATPase, Ca^2+^ -ATPase[KOG0202] |
| TRINITY_DN11871_c0_g1_i1_2 | Up | Inf | Ca^2+^  transporting ATPase [KOG0202] |
| TRINITY_DN11939_c0_g1_i1_3 | Up | Inf | Ca^2+^  transporting ATPase [KOG0202] |
| TRINITY_DN1251_c0_g1_i1_4 | Up | Inf | Ca^2+^  transporting ATPase [KOG0202] |
| TRINITY_DN1251_c0_g2_i1_4 | Up | Inf | Ca^2+^  transporting ATPase [KOG0202] |
| TRINITY_DN13774_c0_g1_i1_1 | Up | Inf | Ca^2+^  transporting ATPase [KOG0202] |
| TRINITY_DN14814_c0_g1_i1_2 | Up | Inf | Ca^2+^  transporting ATPase [KOG0202] |
| TRINITY_DN14978_c0_g1_i1_1 | Up | Inf | Ca^2+^  transporting ATPase [KOG0202] |
| TRINITY_DN15015_c0_g1_i1_1 | Up | Inf | Ca^2+^  transporting ATPase [KOG0202] |
| TRINITY_DN1923_c0_g1_i1_1 | Up | Inf | Ca^2+^  transporting ATPase [KOG0202] |
| TRINITY_DN2601_c0_g1_i1_1 | Up | Inf | Ca^2+^  transporting ATPase [KOG0202] |
| TRINITY_DN285_c0_g2_i1_1 | Up | Inf | Ca^2+^  transporting ATPase [KOG0202] |
| TRINITY_DN41_c0_g1_i1_1 | Up | Inf | Ca^2+^  transporting ATPase [KOG0202] |
| TRINITY_DN12985_c0_g1_i1_1 | Up | 702.14 | Calcium transporting ATPase [KOG0204] |
| TRINITY_DN14885_c0_g1_i1_1 | Up | Inf | Calcium transporting ATPase [KOG0204] |
| TRINITY_DN15232_c0_g1_i1_4 | Up | Inf | Calcium transporting ATPase [KOG0204] |
| TRINITY_DN2487_c0_g1_i1_1 | Up | 995.05 | Cation transport ATPase [PF13246.6] |
| TRINITY_DN2497_c0_g1_i2_3 | Up | 1279.67 | Cation transport ATPase [PF13246.6] |
| TRINITY_DN1072_c0_g1_i1_2 | Up | Inf | Sodium/calcium exchanger protein [PF01699.24] |
| TRINITY_DN6533_c0_g1_i4_2 | Up | 2.15 | Sodium/calcium exchanger protein [PF01699.24] |
| TRINITY_DN6816_c0_g1_i13_1 | Up | 2.22 | Sodium/calcium exchanger protein [PF01699.24] |
| TRINITY_DN7981_c0_g1_i6_2 | Up | 30.33 | Ca^2+^-binding protein Regucalcin/SMP30 [KOG4499] |

**Table S11.** List and annotation of DEGs involved in oxidative stress response and signal transduction in *S. vaninii* MF5 induced by *F. solani* MF20.

| **Unigene ID** | **Up/Down** | **Fold Change** | **Description** |
| --- | --- | --- | --- |
| Response to oxidative stress | | | |
| TRINITY_DN15336_c0_g1_i1_4 | Up | Inf | Glutathione peroxidase, GPX [KOG1651] |
| TRINITY_DN3714_c0_g1_i1_1 | Up | Inf | Glutathione peroxidase, GPX [KOG1651] |
| TRINITY_DN10923_c0_g2_i4_1 | Up | 3.45 | Glutathione peroxidase, GPX [KOG1651] |
| TRINITY_DN10783_c0_g1_i1_3 | Inf | Inf | Catalase-Peroxidase, CAT-POD [COG0376] |
| TRINITY_DN2150_c0_g1_i1_1 | Up | Inf | Catalase-Peroxidase, CAT-POD [COG0376] |
| TRINITY_DN14375_c0_g1_i1_4 | Up | 1572.69 | Peroxidase, POD[PF00141.23] |
| TRINITY_DN12120_c0_g1_i1_1 | Up | 1072.40 | Peroxidase, POD [PF00141.23] |
| TRINITY_DN2239_c0_g1_i1_3 | Up | Inf | Peroxidase, POD[PF00141.23] |
| TRINITY_DN2239_c1_g1_i1_3 | Up | Inf | Peroxidase, POD[PF00141.23] |
| TRINITY_DN10938_c0_g1_i1_2 | Up | Inf | Alkyl hydroperoxide reductase, AHR [KOG0852] |
| TRINITY_DN164_c0_g1_i2_1 | Up | Inf | Peptide-methionine (R)-S-oxide reductase activity, PMSOR [GO:0033743] |
| TRINITY_DN8280_c0_g1_i1_4 | Up | 2.01 | Catalase, CAT [SBW02026.1] |
| Other enzymes related to response processes | | | |
| TRINITY_DN1119_c0_g1_i1_2 | Up | Inf | ATP-dependent serine protease, PPase [COG0466] |
| TRINITY_DN1348_c0_g1_i1_2 | Up | Inf | ATP-dependent serine protease, PPase [COG0466] |
| TRINITY_DN14775_c0_g1_i1_4 | Up | Inf | ATP-dependent serine protease, PPase[COG0466] |
| TRINITY_DN15887_c0_g1_i1_1 | Up | Inf | ATP-dependent serine protease, PPase [COG0466] |
| TRINITY_DN2611_c0_g1_i1_1 | Up | Inf | ATP-dependent serine protease PPase [COG0466] |
| TRINITY_DN1292_c0_g1_i1_1 | Up | Inf | Mitogen-activated protein kinase kinase kinase, MAPKK [ENOG410XQGS] |
| TRINITY_DN6472_c0_g1_i4_2 | Up | 2.04 | Serine/threonine-protein kinase [P54736\|PKN2_MYXXA] |
| TRINITY_DN13078_c0_g1_i1_4 | Up | Inf | NADPH Oxidase, NOX-1 [ENOG410XNZY] |
| TRINITY_DN13411_c0_g1_i1_4 | Up | Inf | NADPH Oxidase, NOX-2 [ENOG410XNZY] |
| TRINITY_DN14585_c0_g1_i1_4 | Up | Inf | Serine Threonine protein kinase [COG0515] |
| TRINITY_DN15120_c0_g1_i1_2 | Up | Inf | Serine Threonine protein kinase [COG0515] |
| TRINITY_DN11747_c0_g1_i1_4 | Up | Inf | Serine threonine-protein kinase [ENOG410XQ0C] |
| TRINITY_DN11940_c0_g1_i1_1 | Up | Inf | Protein serine/threonine kinase activity [ENOG410XNPH] |
| TRINITY_DN13269_c0_g1_i1_1 | Up | Inf | Serine Threonine protein kinase [ENOG410XQ0D] |
| TRINITY_DN14213_c0_g1_i1_4 | Up | Inf | Serine threonine-protein kinase [ENOG410XQC0] |
| TRINITY_DN14601_c0_g1_i1_1 | Up | Inf | Serine threonine-protein kinase [ENOG410XQC0] |
| TRINITY_DN2202_c0_g1_i1_1 | Up | Inf | Protein serine/threonine kinase activity [ENOG410XNPH] |
| TRINITY_DN1022_c0_g1_i1_3 | Up | Inf | GTP-binding Protein [COG1100 ] |
| TRINITY_DN15515_c0_g1_i1_1 | Up | Inf | GTP-binding Protein [COG1100 ] |
| TRINITY_DN13091_c0_g1_i1_2 | Up | Inf | CAMP-dependent protein kinase, PKA [KOG0616] |
| TRINITY_DN15011_c0_g1_i1_4 | Up | Inf | ABC transporter [PF00005.27] |
| TRINITY_DN13506_c0_g1_i1_4 | Up | Inf | ABC transporter [PF00664.23] |
| TRINITY_DN2042_c0_g1_i1_2 | Up | Inf | ABC transporter [PF00005.27] |
| TRINITY_DN2241_c0_g1_i1_1 | Up | Inf | ABC transporter [PF00005.27] |
| TRINITY_DN843_c0_g1_i2_1 | Up | Inf | GTP-binding protein SEC4 [KOG0078] |
| TRINITY_DN13582_c0_g1_i1_2 | Up | Inf | P21 protein (Cdc42 Rac)-activated kinase, Cdc42 Rac [ENOG410XP4K] |
| TRINITY_DN14197_c0_g1_i1_2 | Up | Inf | FAD-binding protein DIMINUTO [KOG1262] |
| TRINITY_DN11473_c0_g1_i1_1 | Up | Inf | FAD binding domain [PF01565.23] |
| TRINITY_DN11051_c0_g1_i1_2 | Up | Inf | Ribose/Galactose Isomerase [PF02502.18] |
| TRINITY_DN11363_c0_g1_i1_3 | Up | Inf | Glutamine synthetase [COG0174] |

**Table S15.** Top 30 most significantly up-regulated differential metabolites (DMs) in *S. vaninii* MF5 induced by co-culture with *F. solani* MF20.

| **Metabolites** | **Up/Down** | **VIP** | **Log_2_ (FC)** | **Formula** | **Class** |
| --- | --- | --- | --- | --- | --- |
| Capmatinib | Up | 5.07 | 37.29 | C23H17FN6O | Quinolines and derivatives |
| 20-F4t-Neurop | Up | 3.88 | 36.79 | C35H52O9 | Fatty Acyls |
| Isoorientin 2''-O-Gallate | Up | 4.04 | 36.64 | C28H24O15 | Polyketides |
| 6alpha-Hydroxyphaseollin | Up | 3.38 | 36.08 | C20H18O5 | Isoflavonoids |
| Myricetin 3-Sambubioside | Up | 3.24 | 35.99 | C26H28O17 | Polyketides |
| Luteolin 3'-Methyl Ether 7-Glucuronosyl-(1->2)-Glucuronide | Up | 2.88 | 35.67 | C28H28O18 | Polyketides |
| (3r,5r)-3-Butyl-3-Ethyl-5-Phenyl-4,5-Dihydro-2h-1$L^{6},4-Benzothiazepine 1,1-Dioxide | Up | 2.54 | 35.39 | C21H27NO2S | Benzothiazepines |
| Trimethylolpropane Trimethacrylate | Up | 2.24 | 35.04 | C18H26O6 | Unclassified |
| Firocoxib | Up | 2.04 | 35.04 | C17H20O5S | Benzene and substituted derivatives |
| N-(2-Hydroxyethyl)Ethylenediaminetriacetic Acid | Up | 2.25 | 34.91 | C10H18N2O7 | Carboxylic acids and derivatives |
| Pachymic Acid | Up | 2.21 | 34.88 | C33H52O5 | Prenol lipids |
| Methylhildgardtol B | Up | 2.01 | 34.84 | C22H24O4 | Polyketides |
| Urolithin A 3,8-O-Diglucuronide | Up | 1.97 | 34.75 | C25H24O16 | Organooxygen compounds |
| Oxacillin | Up | 2.09 | 34.74 | C19H19N3O5S | Carboxylic acids and derivatives |
| Α-D-Glcnac3s-(1→4)-Β-D-Glca(1→3)-Β-D-Gal(1→3)-Β-D-Gal(1→4)-D-Xyl | Up | 2.05 | 34.66 | C31H51NO29S | Organooxygen compounds |
| Ipsapirone | Up | 1.88 | 34.58 | C19H23N5O3S | Diazinanes |
| Mono-Isopropyl-Disopyramide | Up | 1.74 | 34.19 | C18H23N3O | Carboxylic acids and derivatives |
| Defactinib | Up | 1.72 | 34.13 | C20H21F3N8O3S | Benzene and substituted derivatives |
| PA(20:1(11Z)/18:1(12Z)-2OH(9,10)) | Up | 1.33 | 33.96 | C41H77O10P | Unclassified |
| Protocatechuic Acid 3-O-Sulfate | Up | 1.49 | 33.75 | C7H6O7S | Organic sulfuric acids and derivatives |
| Levonantradol | Up | 1.22 | 33.72 | C27H35NO4 | Quinolines and derivatives |
| (4r,6s)-6-[(E)-2-[2-(4-Fluoro-3-Methylphenyl)-4,6-Dimethylphenyl]Ethenyl]-4-Hydroxyoxan-2-One | Up | 1.31 | 33.65 | C22H23FO3 | Benzene and substituted derivatives |
| Disialyllactose | Up | 1.43 | 33.60 | C34H56N2O27 | Organooxygen compounds |
| Methyl 3,4,6-Tri-O-Galloyl-Beta-D-Glucopyranoside | Up | 1.20 | 33.58 | C28H26O18 | Tannins |
| N-[[2-(4-Amino-1,2,5-Oxadiazol-3-Yl)-1-Ethylimidazo[5,4-D]Pyridin-7-Yl]Methyl]Piperidin-4-Amine | Up | 1.34 | 33.50 | C16H22N8O | Imidazopyridines |
| Distemonanthin | Up | 1.33 | 33.42 | C17H10O9 | Polyketides |
| Luxabendazole | Up | 1.22 | 33.39 | C15H12FN3O5S | Benzimidazoles |
| 9h-Fluorene-9-Carboxamide, 9-(3-Aminopropyl)- | Up | 1.23 | 33.28 | C17H18N2O | Fluorenes |
| PPA(16:0/18:1(9Z)) | Up | 1.16 | 33.24 | C37H72O11P2 | Glycerophospholipids |
| Befloxatone | Up | 1.13 | 33.10 | C15H18F3NO5 | Phenol ethers |

**Table S16.** List and annotation of DEGs involved in the terpenoid backbone biosynthesis pathway of *S. vaninii* MF5 induced by *F. solani* MF20.

| **Unigene ID** | **Up/down** | **Fold Change** | **Description** |
| --- | --- | --- | --- |
| TRINITY_DN10102_c0_g1_i13_1 | Up | 3.19 | Acetyl-CoA C-acetyltransferase, AACT-1[40/70(57.14)] |
| TRINITY_DN10982_c0_g1_i1_2 | Up | Inf | Acetyl-CoA C-acetyltransferase, AACT-2 [222/396(56.06)] |
| TRINITY_DN6565_c0_g3_i1_3 | Down | -78.19 | Acetyl-CoA C-acetyltransferase, AACT-3 [28/46(60.87)] |
| TRINITY_DN7091_c1_g2_i4_2 | Down | -12.62 | Acetyl-CoA C-acetyltransferase, AACT-4 [28/46(60.87)] |
| TRINITY_DN841_c0_g1_i2_2 | Up | 698.88 | Acetyl-CoA C-acetyltransferase, AACT-5 [230/414(55.56)] |
| TRINITY_DN8582_c0_g1_i22_3 | Down | -2.21 | Acetyl-CoA C-acetyltransferase, AACT-6 [36/60(60.00)] |
| TRINITY_DN4452_c0_g1_i3_4 | Up | 973.37 | Hydroxymethylglutaryl-CoA synthase, HMGS [152/448(33.93)] |
| TRINITY_DN12102_c0_g1_i1_2 | Up | Inf | Hydroxymethylglutaryl-CoA reductase, HMGR-1 [79/134(58.96)] |
| TRINITY_DN12379_c0_g1_i1_1 | Up | 142.94 | Hydroxymethylglutaryl-CoA reductase, HMGR-2 [81/223(36.32)] |
| TRINITY_DN14439_c0_g1_i1_1 | Up | Inf | Hydroxymethylglutaryl-CoA reductase, HMGR-3 [68/120(56.67)] |
| TRINITY_DN13899_c0_g1_i1_2 | Up | 295.38 | Mevalonate kinase, MVK [113/366(30.87)] |
| TRINITY_DN1011_c0_g1_i1_2 | Up | Inf | Diphosphomevalonate decarboxylase, DPMDD-1 [71/167(42.51)] |
| TRINITY_DN15469_c0_g1_i1_1 | Up | Inf | Diphosphomevalonate decarboxylase, DPMDD-2 [35/64(54.69)] |
| TRINITY_DN1909_c0_g1_i1_2 | Up | Inf | Diphosphomevalonate decarboxylase, DPMDD-3 [28/42(66.67)] |
| TRINITY_DN11367_c0_g1_i1_4 | Up | Inf | Isopentenyl diphosphate isomerase, IDI [76/184(41.30)] |
| TRINITY_DN13134_c0_g1_i1_1 | Up | Inf | Geranylgeranyl diphosphate synthase, GGPS-1 [82/269(30.48)] |
| TRINITY_DN2222_c0_g1_i1_1 | Up | Inf | Geranylgeranyl diphosphate synthase, GGPS-2 [64/198(32.32)] |
| TRINITY_DN2924_c0_g1_i2_4 | Down | -2.21 | Geranylgeranyl diphosphate synthase, GGPS-3 [80/266(30.08)] |
| TRINITY_DN12264_c0_g1_i1_4 | Up | Inf | All-trans-nonaprenyl-diphosphate synthase, NPPS-1 [26/52(50.00)] |
| TRINITY_DN240_c0_g1_i1_1 | Up | Inf | All-trans-nonaprenyl-diphosphate synthase, NPPS-2 [73/133(54.89)] |
| TRINITY_DN10350_c0_g3_i6_4 | Up | 6.42 | None |
| TRINITY_DN1891_c0_g1_i1_4 | Up | 2.33 | Nuclear architecture related protein [KOG2439] |
| TRINITY_DN2002_c0_g1_i1_1 | Up | 150.97 | Ankyrin repeat and DHHC-type Zn-finger domain containing proteins [KOG0509] |
| TRINITY_DN3366_c0_g1_i1_1 | Up | 2.89 | Phosphatidylinositol-4-phosphate 5-kinase [COG5253] |
| TRINITY_DN5131_c0_g1_i1_1 | Down | -2.33 | None |
| TRINITY_DN6496_c0_g2_i1_3 | Down | -2.51 | None |
| TRINITY_DN7070_c0_g1_i3_4 | Up | 5.36 | Phosphatidylserine decarboxylase [89/202(44.06)] |
| TRINITY_DN9969_c0_g1_i4_4 | Down | -2.80 | None |
| TRINITY_DN10618_c0_g3_i4_1 | Down | -2.06 | Other AgaK1 protein kinase [ENOG4110BRX] |
| TRINITY_DN11144_c0_g2_i1_1 | Up | 76.21 | None |
| TRINITY_DN13684_c0_g1_i1_2 | Up | 54.88 | None |
| TRINITY_DN6766_c0_g1_i2_4 | Down | -12.69 | None |
| TRINITY_DN9874_c1_g1_i1_1 | Up | 2.16 | Predicted spermine/spermidine synthase [KOG2352] |
| TRINITY_DN13379_c0_g1_i1_2 | Up | Inf | Glutathione synthase [56/148(37.84)] |
| TRINITY_DN15959_c0_g1_i1_1 | Up | Inf | Glutamate synthase [KOG0399] |
| TRINITY_DN3555_c0_g1_i2_2 | Up | 586.52 | Peptidylprolyl isomerase [WP_106474478.1] |
| TRINITY_DN12008_c0_g1_i1_1 | Up | Inf | 1,3-beta-glucan synthase component [PF02364.15] |
| TRINITY_DN12520_c0_g1_i1_1 | Up | Inf | Hypothetical protein [TKW60297.1] |
| TRINITY_DN4302_c0_g1_i7_4 | Up | Inf | Calcium/proton exchanger [RZD45892.1] |

**Table S17.** List of differential metabolites (DMs) identified as terpenoids in *S. vaninii* MF5 induced by co-culture with *F. solani* MF20.

| **Metabolites** | **Up/Down** | **VIP** | **Fold Change** | **Formula** | **Class** |
| --- | --- | --- | --- | --- | --- |
| Farnesol | Up | 2.77 | 688.65 |  | Sesquiterpenoids |
| 15-Hydroxydehydroabietic Acid | Down | 1.00 | -2.62 |  | Diterpenoids |
| 5-Hydroxyprocurcumenol | Up | 13.40 | 38.91 | C15H22O3 | Sesquiterpenoids |
| Hericene A | Up | 12.78 | 85.30 | C35H56O5 | Monoterpenoids |
| Curdione | Up | 6.34 | 20.01 | C15H24O2 | Sesquiterpenoids |
| Dehydronootkatone | Up | 5.56 | 186.41 | C15H20O | Sesquiterpenoids |
| Ecabet | Down | 2.96 | -2.18 | C20H28O5S | Diterpenoids |
| Curcumadiol | Up | 2.91 | 17.93 | C15H26O2 | Sesquiterpenoids |
| Calamendiol | Up | 2.86 | 52.36 | C15H26O2 | Sesquiterpenoids |
| Valerenolic Acid | Up | 2.66 | 1.81 | C15H22O3 | Sesquiterpenoids |
| Dolichyl B-D-Glucosyl Phosphate | Up | 2.34 | 4066.11 | C21H39O9P | Sesquiterpenoids |
| Dtdp-4-Dehydro-6-Deoxy-Beta-L-Mannose | Down | 2.24 | -13.86 | C16H24N2O15P2 | Diterpenoids |
| Pachymic Acid | Up | 2.21 | 3.16×10^10^ | C33H52O5 | Triterpenoids |
| Trichoderonin | Up | 2.21 | 8.58 | C17H24O4 | Sesquiterpenoids |
| 3,11,12-Trihydroxy-1(10)-Spirovetiven-2-One | Up | 2.11 | 88.11 | C15H24O4 | Sesquiterpenoids |
| 1alpha-1-Hydroxy-2,4(18),11(13)-Eudesmatrien-12-Oic Acid | Up | 2.05 | 1.78 | C15H20O3 | Sesquiterpenoids |
| (13r,14r)-7-Labdene-13,14,15-Triol | Down | 2.03 | -1.31 | C20H36O3 | Diterpenoids |
| Acetylvalerenolic Acid | Up | 1.67 | 3.99 | C17H24O4 | Sesquiterpenoids |
| 5beta-1,3,7(11)-Eudesmatrien-8-One | Up | 1.58 | 5.67 | C15H20O | Sesquiterpenoids |
| Glycinoeclepin B | Down | 1.56 | -45.85 | C31H42O9 | Sesquiterpenoids |
| Procurcumenol | Up | 1.48 | 21.76 | C15H22O2 | Sesquiterpenoids |
| 8,12-Epoxy-4(15),7,11-Eudesmatrien-1-One | Up | 1.35 | 1.87 | C15H18O2 | Sesquiterpenoids |
| Alpha-Terpinene | Up | 1.34 | 6.10 | C10H16 | Monoterpenoids |
| Eremopetasidione | Up | 1.26 | 1.66 | C14H20O3 | Sesquiterpenoids |
| Isomasticadienonalic Acid | Up | 1.25 | 15.62 | C30H44O4 | Triterpenoids |
| 7-Hydroxycostol | Up | 1.16 | 2.14 | C15H24O2 | Sesquiterpenoids |
| 3beta-Acetoxy-19alpha-Hydroxy-12-Ursene | Up | 1.15 | 8.57×10^7^ | C32H52O3 | Triterpenoids |
| 3-Methyl-4-(2,6,6-Trimethyl-2-Cyclohexen-1-Yl)-3-Buten-2-One | Up | 1.12 | 11.38 | C14H22O | Sesquiterpenoids |
| L-Monomenthyl Glutarate | Up | 1.10 | 3.82 | C15H26O4 | Monoterpenoids |
| Musabalbisiane C | Up | 1.01 | 2.47 | C28H40O12 | Diterpenoids |

**Table S19.** List of differential metabolites (DMs) identified as flavonoids and isoflavonoids in *S. vaninii* MF5 induced by co-culture with *F. solani* MF20.

| **Metabolites** | **Up/Down** | **VIP** | **Fold Change** | **Formula** | **Class** |
| --- | --- | --- | --- | --- | --- |
| 6'-Malonyltrifolirhizin | Up | 4.86 | 5.24×10^7^ | C25H24O13 | Isoflavonoids |
| 2',4',6',3,4-Pentahydroxy-3'-Geranyl-5-Prenyldihydrochalcone | Down | 4.47 | -61.15 | C30H38O6 | Flavonoids |
| Isorhamnetin 3-(3'''-Ferulylrobinobioside) | Down | 4.43 | -5.09 | C38H40O19 | Flavonoids |
| Clitoriacetal | Down | 4.06 | -5.22 | C19H18O9 | Flavonoids |
| Isoorientin 2''-O-Gallate | Up | 4.04 | 1.07×10^11^ | C28H24O15 | Flavonoids |
| Breverin | Down | 4.03 | -5.74 | C18H16O7 | Flavonoids |
| 6alpha-Hydroxyphaseollin | Up | 3.38 | 7.26×10^10^ | C20H18O5 | Flavonoids |
| Apigenin 7-(3''-Acetyl-6''-E-P-Coumaroylglucoside) | Down | 3.33 | -4.00 | C32H28O13 | Flavonoids |
| Myricetin 3-Sambubioside | Up | 3.24 | 6.82×10^10^ | C26H28O17 | Flavonoids |
| Catechin 5-O-Beta-D-Glucopyranoside-4'-Me | Down | 3.10 | -327.21 | C22H26O11 | Flavonoids |
| Quercetin 3-(3'',6''-Di-P-Coumarylglucoside) | Down | 2.90 | -3,091.72 | C39H32O16 | Flavonoids |
| Luteolin 3'-Methyl Ether 7-Glucuronosyl-(1->2)-Glucuronide | Up | 2.88 | 5.47×10^10^ | C28H28O18 | Flavonoids |
| Tephrowatsin B | Up | 2.67 | 917.37 | C22H24O3 | Flavonoids |
| Quercetin 3-Sulfate-7-Alpha-Arabinopyranoside | Down | 2.60 | -2.31 | C20H18O14S | Flavonoids |
| 5,7,3'-Trihydroxy-6,4',5'-Trimethoxyflavanone | Down | 2.11 | -27.15 | C18H18O8 | Flavonoids |
| Epigallocatechin Gallate | Down | 2.07 | -5.24 | C22H18O11 | Flavonoids |
| Quercetin 3-(2''-P-Hydroxybenzoyl-4''-P-Coumarylrhamnoside) | Down | 2.01 | 2.34×10^-11^ | C37H30O15 | Flavonoids |
| Methylhildgardtol B | Up | 2.07 | 3.08×10^10^ | C22H24O4 | Flavonoids |
| 6,8-Dimethylapigenin | Down | 1.83 | -28.42 | C17H14O5 | Flavonoids |
| Myricetin 3-(2''-Galloylgalactoside) | Up | 1.82 | 3.93 | C28H24O17 | Flavonoids |
| Dalpatein | Down | 1.79 | -11.52 | C18H14O7 | Flavonoids |
| Puddumin A | Down | 1.69 | -13.31 | C22H24O10 | Flavonoids |
| Petunidin 3-(4'''-P-Coumaroyl-Rutinoside) | Down | 1.61 | -179.82 | C37H39O18+ | Flavonoids |
| Kolaflavanone | Down | 1.60 | -7.30 | C31H24O12 | Flavonoids |
| Cyanidin 3-(6''-Dioxalylglucoside) | Down | 1.55 | -9.88 | C25H21O17+ | Flavonoids |
| Isochamanetin | Up | 1.55 | 7.66 | C22H18O5 | Flavonoids |
| Catechin | Up | 1.52 | 6.91 | C15H14O6 | Flavonoids |
| Cyanidin 5-O-Beta-D-Glucoside | Down | 1.52 | -11.36 | C21H19O11- | Flavonoids |
| Lophirone J | Down | 1.47 | -3.36 | C25H20O5 | Flavonoids |
| Coumestrin | Down | 1.47 | -21.71 | C21H18O10 | Coumestans |
| Quercetin 3-O-Xylosyl-Glucuronide | Up | 1.46 | 80.74 | C26H26O17 | Flavonoids |
| Vitexin 2''-O-(E)-Ferulate | Up | 1.44 | 3.84 | C31H28O13 | Flavonoids |
| 2h-1-Benzopyran-7-Ol, 3-(4-Methoxyphenyl)-4-((4-(2-(1-Piperidinyl)Ethoxy)Phenyl)Methyl)- | Up | 1.39 | 25.97 | C30H33NO4 | Isoflavonoids |
| Barpisoflavone A | Down | 1.35 | -28.27 | C16H12O6 | Isoflavonoids |
| Hispidulin 7-(6''-E-P-Coumaroylglucoside) | Down | 1.34 | -6.91 | C31H28O13 | Flavonoids |
| Distemonanthin | Up | 1.33 | 1.15×10^10^ | C17H10O9 | Flavonoids |
| Apigenin | Down | 1.31 | -39.37 | C15H10O5 | Flavonoids |
| 4'-Hydroxy-5,7-Dimethoxyflavan | Up | 1.31 | 52.10 | C17H18O4 | Flavonoids |
| Petunidin 3-(6''-Acetylglucoside) | Up | 1.28 | 203.79 | C24H24O13 | Flavonoids |
| Rhusflavanone | Down | 1.25 | -5.25 | C30H22O10 | Flavonoids |
| Glabrescin | Up | 1.24 | 6.25 | C23H20O7 | Flavonoids |
| Isocoreopsin | Down | 1.15 | -4.38 | C21H22O10 | Flavonoids |
| 3beta,4beta,5-Trimethoxy-4'-Hydroxy- (6:7)-2,2-Dimethylpyranoflavan | Down | 1.13 | -1.22 | C23H26O6 | Flavonoids |
| Kaempferol | Down | 1.11 | -25.94 | C15H10O6 | Flavonoids |
| Sakuranin | Down | 1.07 | -19.07 | C22H24O10 | Flavonoids |
| Kanugin | Down | 1.02 | -9.92 | C19H16O7 | Flavonoids |
| Chrysoeriol 7-(3''-Z-P-Coumaroylglucoside) | Down | 1.01 | -8.07 | C31H28O13 | Flavonoids |
| Kaempferol 3-(2''-Galloyl-Alpha-L-Arabinopyranoside) | Up | 1.00 | 98.13 | C27H22O14 | Flavonoids |

**Table S20.** List and annotation of DEGs potentially involved in flavonoid and isoflavonoid biosynthesis in *S. vaninii* MF5 induced by *F. solani* MF20.

| **Unigene ID** | **Up/down** | **Fold Change** | **Description** |
| --- | --- | --- | --- |
| TRINITY_DN8883_c0_g1_i4_2 | Down | -15.19 | Phenylalanine ammonia-lyase, PAL-1 [126/417(30.22)] |
| TRINITY_DN7523_c0_g1_i1_3 | Down | -16.02 | Phenylalanine ammonia-lyase, PAL-2 [B2J528\|PAL_NOSP7] |
| TRINITY_DN10066_c0_g1_i9_3 | Up | 1.06 | Cinnamoyl-CoA reductase, CCR-1 [KOG1502] |
| TRINITY_DN10066_c0_g2_i2_3 | Up | 1.07 | Cinnamoyl-CoA reductase, CCR-2 [KOG1502] |
| TRINITY_DN10520_c0_g1_i10_2 | Up | 1.12 | Cinnamoyl-CoA reductase, CCR-3 [KOG1502] |
| TRINITY_DN10769_c0_g1_i28_4 | Down | -1.03 | Cinnamoyl-CoA reductase, CCR-4 [KOG1502] |
| TRINITY_DN10769_c0_g2_i1_4 | Down | -1.12 | Cinnamoyl-CoA reductase, CCR-5 [KOG1502] |
| TRINITY_DN3262_c0_g1_i1_3 | Up | 1.19 | Cinnamoyl-CoA reductase, CCR-6 [KOG1502] |
| TRINITY_DN7505_c0_g1_i4_1 | Up | 358.24 | Cinnamoyl-CoA reductase, CCR-7 [KOG1502] |
| TRINITY_DN8713_c0_g1_i1_4 | Up | 1.40 | Cinnamoyl-CoA reductase, CCR-8 [KOG1502] |
| TRINITY_DN10422_c0_g2_i3_1 | Up | 9.13 | Isoflavone reductase, IFR-1 [ENOG410Y0DY] |
| TRINITY_DN10909_c0_g5_i2_1 | Up | 1.07 | Isoflavone reductase, IFR-2 [ENOG4111WR8] |
| TRINITY_DN11735_c0_g1_i1_1 | Up | Inf | Isoflavone reductase, IFR-3 [ENOG411028Q] |
| TRINITY_DN8276_c0_g1_i5_4 | Up | 3.18 | Isoflavone reductase, IFR-4 [ENOG41129ZG] |
| TRINITY_DN8930_c0_g2_i1_1 | Up | 9.28 | Isoflavone reductase, IFR-5 [ENOG41129ZG] |
| TRINITY_DN10203_c1_g1_i1_4 | Down | -2.18 | Fungal hydrophobin [PF01185.18] |
| TRINITY_DN10848_c0_g1_i15_4 | Up | 2.89 | Metal ion binding [GO:0046872] |
| TRINITY_DN14260_c0_g1_i1_4 | Up | 61.40 | Conserved hypothetical, protein [ENOG410YFQA] |
| TRINITY_DN4052_c0_g2_i2_2 | Down | -2.14 | None |
| TRINITY_DN5032_c0_g1_i1_2 | Up | 2.41 | None |
| TRINITY_DN6004_c0_g1_i5_3 | Up | 2.11 | None |
| TRINITY_DN7270_c0_g1_i9_2 | Up | 2.30 | Fungal Zn(2)-Cys(6) binuclear cluster domain [PF00172.18] |
| TRINITY_DN10334_c0_g3_i1_4 | Down | -15.59 | None |
| TRINITY_DN12708_c0_g1_i1_1 | Up | Inf | Hydantoinase/oxoprolinase family protein [WP_142880650.1] |
| TRINITY_DN168_c0_g2_i1_3 | Up | Inf | Transporter [ENOG410XNQK] |
| TRINITY_DN2996_c0_g1_i1_2 | Down | -2.13 | None |
| TRINITY_DN4275_c0_g1_i1_3 | Down | -2.93 | None |
| TRINITY_DN5329_c0_g1_i1_4 | Down | -4.20 | Chromatin structure and dynamics [INFORMATION STORAGE AND PROCESSING] |
| TRINITY_DN5502_c0_g1_i8_1 | Down | -4.56 | None |
| TRINITY_DN9623_c0_g1_i32_3 | Down | -3.97 | Fe^2+^/Zn^2+^ regulated transporter [KOG1558] |
| TRINITY_DN4784_c0_g1_i2_4 | Up | 2.19 | Energy production and conversion [METABOLISM] |
| TRINITY_DN10797_c0_g1_i19_4 | Down | 2.60 | Cytochrome p450 [COG2124] |
| TRINITY_DN7552_c0_g1_i3_4 | Down | -4.70 | None |

**Table S23.** List of differential metabolites (DMs) related to polysaccharide and central carbon metabolism in *S. vaninii* MF5 induced by co-culture with *F. solani* MF20.

| **Metabolites** | **Up/Down** | **VIP** | **Fold Change** | **Formula** | **Class** |
| --- | --- | --- | --- | --- | --- |
| D-Glucose | Down | 1.64 | -21.45 |  | Organooxygen compounds |
| Glucose | Down | 1.28 | -8.11 |  | Organooxygen compounds |
| Fructose 6-Phosphate | Down | 1.16 | -3.66 | C6H13O9P | Organooxygen compounds |
| Malic Acid ((S)-Malate) | Up | 1.29 | 4.38 | C15H24N2O17P2 | Pyrimidine nucleotides |
| Fumaric Acid (Fumarate) | Up | 1.18 | 3.40 |  | Organooxygen compounds |
| Citric Acid (Citrate) | Down | 2.82 | -1.90 |  | Organooxygen compounds |
| Isocitric Acid (Isocitrate) | Down | 1.21 | -1.51 |  | Organooxygen compounds |
| Oxoglutaric Acid (2-Oxoglutarate) | Up | 2.17 | 55.32 |  | Unclassified |
| D-Lactic Acid (D-Lactate) | Down | 1.07 | -3.36 |  | Organooxygen compounds |
| Uridine Diphosphate Glucose (UDP-Glucose) | Down | 1.59 | -3.60 | C6H8O7 | Carboxylic acids and derivatives |
| Glucose 1-Phosphate | Down | 1.31 | -1.55 | C6H8O7 | Carboxylic acids and derivatives |
| Sucrose | Down | 1.64 | -9.53 |  | Keto acids and derivatives |
| D-Tagatose | Down | 1.03 | -2.55 |  | Carboxylic acids and derivatives |
| Galactitol | Down | 1.57 | -1.98 |  | Hydroxy acids and derivatives |
| Arbutin | Up | 1.73 | 18.50 |  | Organooxygen compounds |
| Gluconolactone  (D-Glucono-1,5-lactone) | Down | 1.24 | -5.18 |  | Organooxygen compounds |
| 6-Phosphogluconic Acid  (D-gluconate-6-P) | Down | 1.22 | -3.64 |  | Organooxygen compounds |
| D-(-)-Ribofuranose  (D-Ribose-5-P) | Up | 1.18 | 3.61 | C14H18N2O6 | Carboxylic acids and derivatives |
| D-Ribose | Down | 1.03 | -2.50 |  | Organooxygen compounds |
| Gamma-Glutamyltyrosine  (Ribose-1, 5-P) | Down | 1.04 | -2.31 | C5H12O5 | Organooxygen compounds |
| Deoxyribose | Down | 1.45 | -9.81 |  | Organooxygen compounds |
| Ribitol | Down | 7.18 | -24.47 |  | Organooxygen compounds |
| D-Arabitol | Down | 1.09 | -2.91 |  | Organooxygen compounds |
| Xylulose | Down | 1.48 | -6.76 | C6H14O6 | Organooxygen compounds |
| Xylitol | Down | 1.93 | -22.79 |  | Organooxygen compounds |
| Fructose 1-Phosphate | Down | 1.60 | -7.85 |  | Organooxygen compounds |
| Fructose | Down | 1.19 | -17.04 |  | Hydroxy acids and derivatives |
| D-Fructose | Down | 1.13 | -4.08 | C5H10O4 | Organooxygen compounds |
| D-Mannose 6-Phosphate | Down | 1.08 | -27.74 |  | Organic oxygen compounds |
| Beta-Lactose | Down | 1.90 | -22.50 |  | Organic oxygen compounds |
